# Author Correction: A mycorrhizae-like gene regulates stem cell and gametophore development in mosses

**DOI:** 10.1038/s41467-020-16421-3

**Published:** 2020-06-04

**Authors:** Shuanghua Wang, Yanlong Guan, Qia Wang, Jinjie Zhao, Guiling Sun, Xiangyang Hu, Mark P. Running, Hang Sun, Jinling Huang

**Affiliations:** 1 Key Laboratory for Plant Diversity and Biogeography of East Asia, Yunnan Key Laboratory for Fungal Diversity and Green Development, Kunming Institute of Botany, Chinese Academy of Sciences, Kunming, 650201 China; 2 University of Chinese Academy of Sciences, Beijing, 100049 China; 3 State Key Laboratory of Crop Stress Adaptation and Improvement, Key Laboratory of Plant Stress Biology, School of Life Sciences, Henan University, Kaifeng, 475001 China; 4 Shanghai Key Laboratory of Bio-Energy Crops, School of Life Sciences, Shanghai University, Shanghai, 200444 China; 5 Department of Biology, University of Louisville, Louisville, KY USA; 6 Department of Biology, East Carolina University, Greenville, NC USA

**Keywords:** Evolutionary genetics, Molecular evolution, Plant genetics, Plant evolution, Plant genetics

Correction to: *Nature Communications* 10.1038/s41467-020-15967-6, published online 24 April 2020.

The original version of this Article omitted a reference to previous work in ‘Leebens-Mack, J.H. et al. One thousand plant transcriptomes and the phylogenomics of green plants. *Nature* **574**, 679–685 (2019)’. This has been added as reference 28 in the second sentence of the “Mycorrhizae-like fungal origin of land plant macro2 gene” section of the “Results”: with the PpMACRO2 protein sequence (Genbank accession number: XP_024388278) as query, we performed a BLAST search of the NCBI non-redundant (nr) protein sequence database, the 1000 plants project (OneKP) and other resources, including the recently published genomes of hornworts (*Anthoceros angustus*), ferns (*Azolla filiculoides* and *Salvinia cucullata*) and charophytes (e.g., *Chara braunii*, *Spirogloea muscicola*, *Mesotaenium endlicherianum*, *Mesostigma viride*, and *Chlorokybus atmophyticus*)^23–28^. This has been corrected in the PDF and HTML versions of the Article.

