# Supplementary material for: A mycorrhizae-like gene regulates stem cell and gametophore development in mosses
Source: Nat Commun. 2020 Apr 24;11:2030. doi: 10.1038/s41467-020-15967-6 (PMC7181705; doi:10.1038/s41467-020-15967-6)
Supplement: Supplementary file 1 — Supplementary Information [file 41467_2020_15967_MOESM1_ESM.pdf]

Supplementary Information

for

A mycorrhizae-like gene regulates stem cell and gametophore development in mosses

Wang et al.

Taxonomy	Number of hits	Number of Organisms	Description
⊟root	7927	1856	
. ⊟cellular organisms	7916	1848	
. . ⊟Eukaryota	552	336	
. . . Physcomitrella patens	3	1	Physcomitrella patens hits
. . . ⊟Opisthokonta	547	334	
. . . . ⊟Fungi	515	316	
. . . . . ⊟Fungi incertae sedis	19	10	
. . . . . . ⊞Mucoromycota	17	8	
. . . . . . ⊞Zoopagomycota	2	2	
. . . . . ⊟Dikarya	496	306	
. . . . . . ⊞Agaricomycotina	107	53	
. . . . . . ⊞saccharomyceta	389	253	
. . . . ⊟Metazoa	32	18	
. . . . . ⊟Eumetazoa	30	17	
. . . . . . ⊞Bilateria	19	11	
. . . . . . ⊞Anthozoa	11	6	
. . . . . Amphimedon queenslandica	2	1	Amphimedon queenslandica hits
. . . Planoprotostelium fungivorum	2	1	Planoprotostelium fungivorum hits
. . ⊟Bacteria	1	1507	Bacteria hits
. . . ⊟Terrabacteria group	462	285	
. . . . ⊟Cyanobacteria/Melainabacteria group	116	73	
. . . . . ⊞Cyanobacteria	115	72	
. . . . . Candidatus Melainabacteria bacterium	1	1	Candidatus Melainabacteria bacterium hits
. . . . ⊞Chloroflexi	14	10	
. . . . ⊞Firmicutes	58	39	
. . . . ⊞Deinococcus	15	9	
. . . . ⊞Actinobacteria	255	153	
. . . . Capsulimonas corticalis	4	1	Capsulimonas corticalis hits
. . . ⊟Bacteroidetes/Chlorobi group	94	49	
. . . . ⊞Bacteroidetes	88	48	
. . . . bacterium 336/3	6	1	bacterium 336/3 hits
. . . ⊟Proteobacteria	6668	1111	
. . . . ⊞Gammaproteobacteria	6556	1047	
. . . . ⊞delta/epsilon subdivisions	75	41	
. . . . ⊞Alphaproteobacteria	30	21	
. . . . ⊞Burkholderiaceae	7	2	
. . . ⊟PVC group	122	52	
. . . . ⊞Planctomycetes	112	45	
. . . . ⊞Verrucomicrobia	9	6	
. . . . Candidatus Abyssubacteria bacterium SURF_17	1	1	Candidatus Abyssubacteria bacterium SURF_17 hit
. . . uncultured bacterium	2	1	uncultured bacterium hits
. . . ⊟Treponema	4	4	
. . . . ⊞Treponema saccharophilum	1	2	Treponema saccharophilum hits
. . . . ⊞Treponema primitia	1	2	Treponema primitia hits
. . . Chloracidobacterium sp. CP2_5A	1	1	Chloracidobacterium sp. CP2_5A hits
. . . ⊟unclassified Bacteria	4	3	
. . . . bacterium	2	1	bacterium hits
. . . . ⊞Parcubacteria group	2	2	
. . ⊟Archaea	6	5	
. . . Candidatus Woesearchaeota archaeon	1	1	Candidatus Woesearchaeota archaeon hits
. . . Candidatus Lokiarchaeota archaeon	1	1	Candidatus Lokiarchaeota archaeon hits
. . . ⊟Methanobacteriales	4	3	
. . . . ⊞Methanothermus	2	2	
. . . . Methanothermobacter tenebrarum	2	1	Methanothermobacter tenebrarum hits
. ⊟Viruses	11	8	
. . Phage Gifsy-1	1	1	Phage Gifsy-1 hits
. . ⊟Myoviridae	8	5	
. . . ⊞unclassified Peduovirus	2	2	
. . . Pseudomonas phage Noxifer	2	1	Pseudomonas phage Noxifer hits
. . . Pseudomonas phage PhiPA3	2	1	Pseudomonas phage PhiPA3 hits
. . . Ralstonia phage phiRSL1	2	1	Ralstonia phage phiRSL1 hits
. . Marseillevirus LCMAC201	1	1	Marseillevirus LCMAC201 hits
. . Catovirus CTV1	1	1	Catovirus CTV1 hits

Supplementary Fig. 1. Taxonomic distribution of hits generated from BLASTP search of NCBI non-redundant (nr) protein sequence database. PpMACRO2 (accession number: XP_024388278) was used as query and E-value cutoff=1e-6.

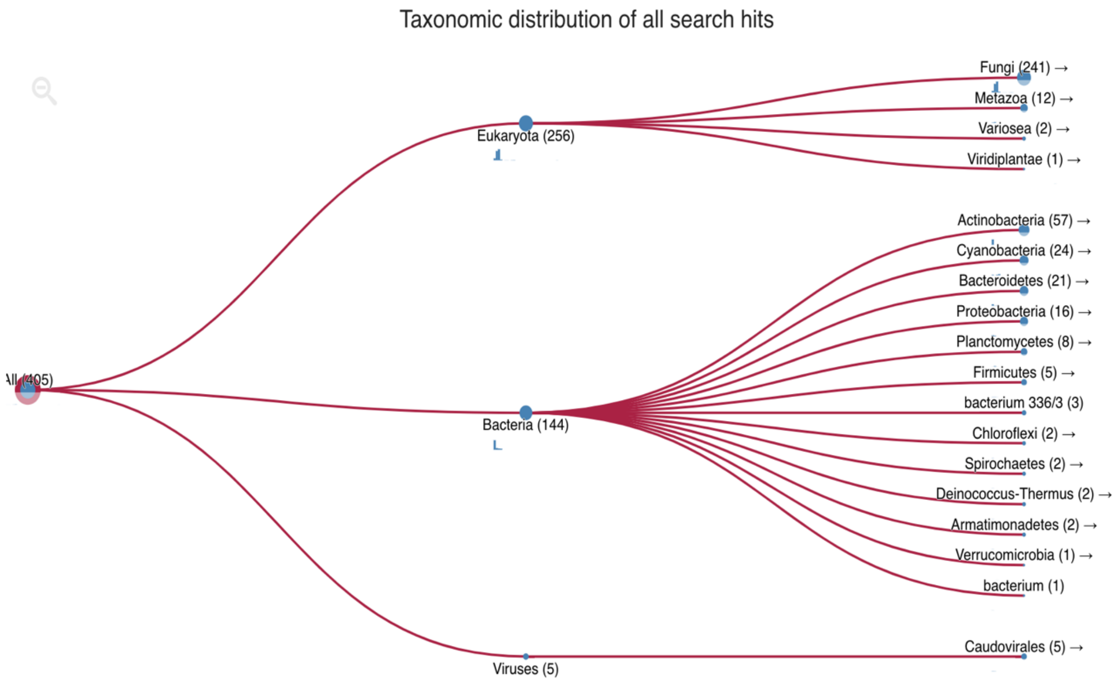

Supplementary Fig. 2. Taxonomic distribution of hits generated from pHMMER search of Reference Proteomes. PpMACRO2 (NCBI accession number: XP_024388278) was used as query and E-value cutoff=1e-6.

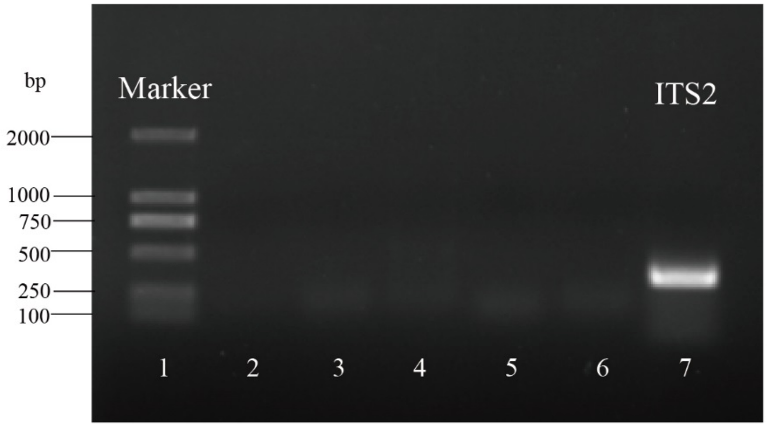

Supplementary Fig. 3. Genomic PCR for *PpMACRO2* homologs in the charophyte alga *Spirogyra sp*. Internal Transcribed Spacer 2 (ITS2) could be amplified from *Spirogyra sp.*, and the fragment length of genomic PCR is in accordance with expectation. Subsequent sequencing result confirmed that this species belongs to the genus *Spirogyra*. However, no sequence could be amplified, using two pairs of primers designed from *S. pratensis* hits obtained from NCBI ESTs (lanes 2-3) and three pairs of primers designed from various other green algal hits (lanes 4-6). This evidence, combined with the absence of *PpMACRO2* homologs from any complete green algal genome generated from axenic cultures, suggests that the green algal hits from OneKP and NCBI ESTs might be due to contamination.

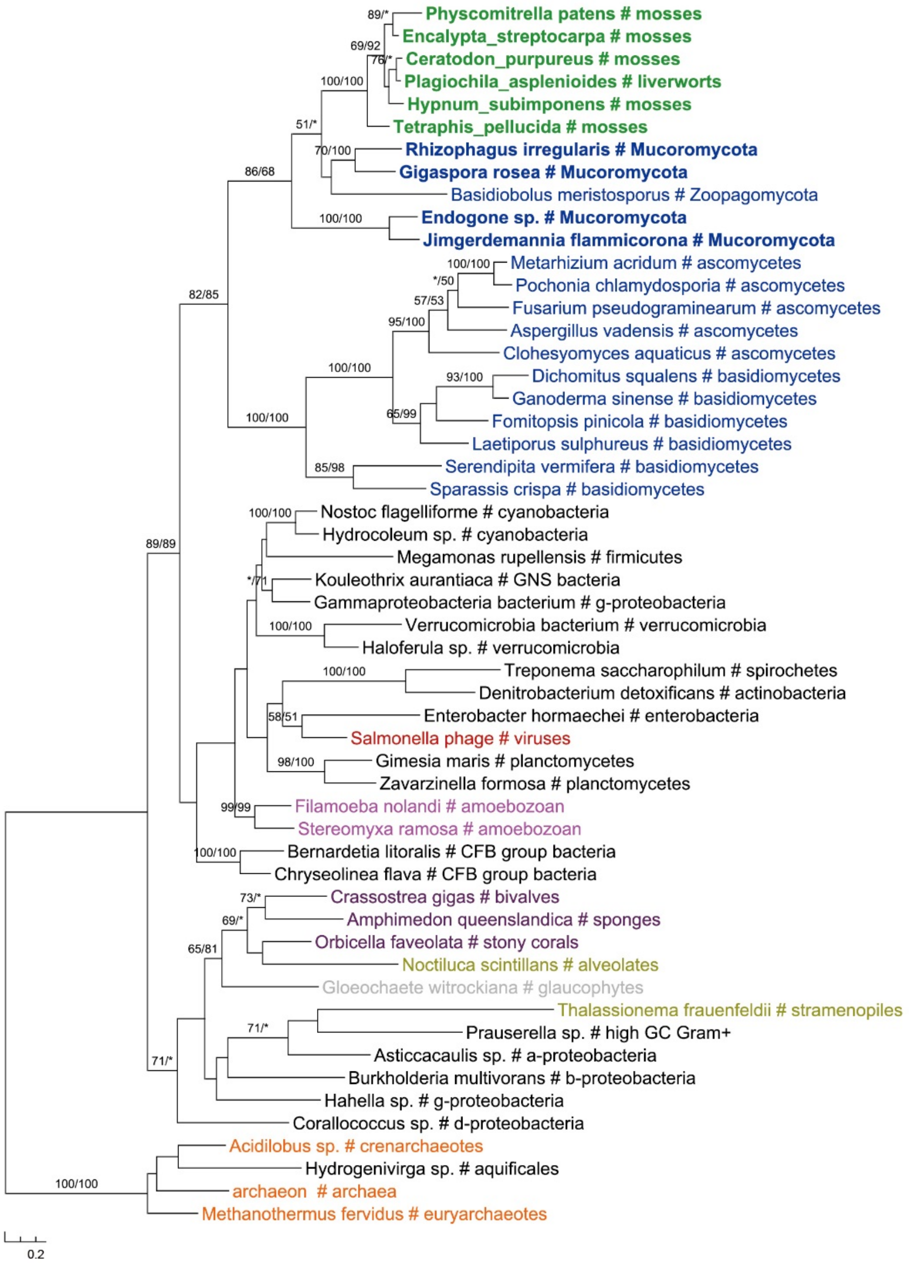

Supplementary Fig. 4. Relationships of PpMACRO2 and homologs. Numbers above branches show bootstrap support values from maximum likelihood and distance analyses, respectively. Asterisks show values lower than 50%. Data do not include green algal sequences of which the identity cannot be confirmed. Please see **Supplementary Fig. 5** for gene tree that includes green algal sequences.

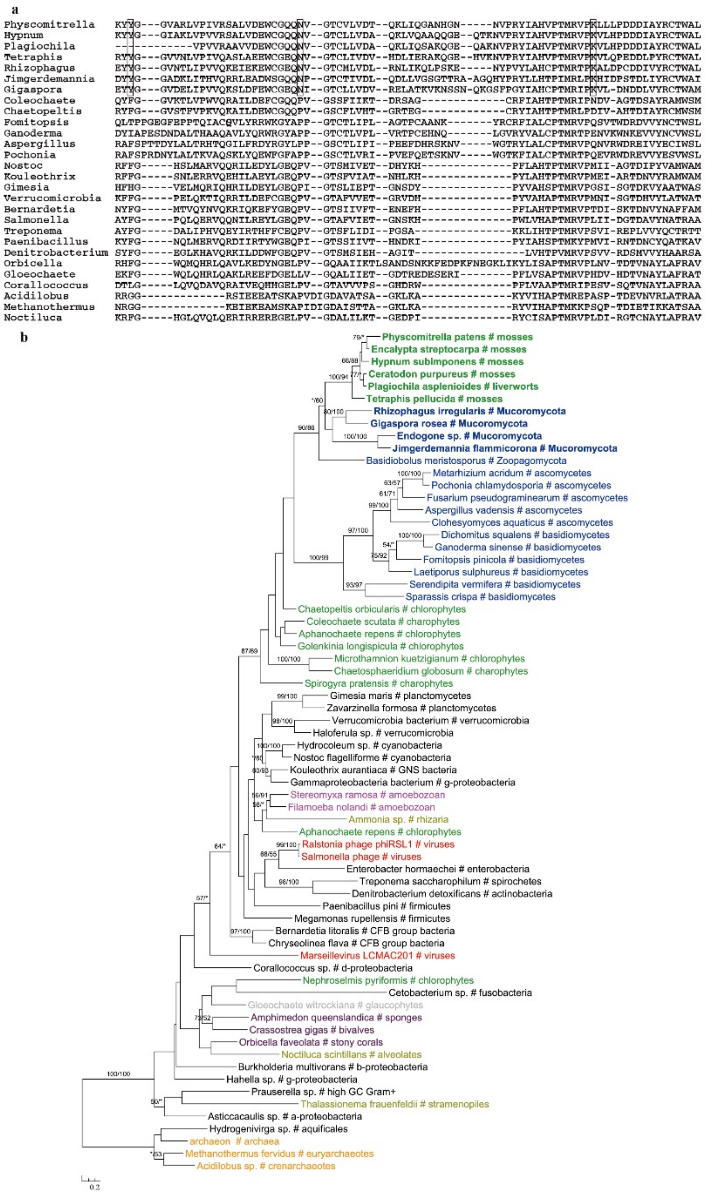

Supplementary Fig. 5. Relationships of PpMACRO2 and homologs, shown in multiple sequence alignment (**a**) and molecular phylogeny with green algal hits (**b**). Boxes in the alignment show amino acids uniquely shared by bryophytes and Mucoromycota fungi. Numbers above branches in the molecular phylogeny show bootstrap support values from maximum likelihood and distance analyses, respectively. Asterisks show values lower than 50%. Note again that the green algal sequences were identified from OneKP and NCBI EST datasets, but no hits could be identified from the many complete genomes of green algae in NCBI, Phytozome and other resources. Please see Supplementary **Fig. 4** for gene tree without green algal sequences.

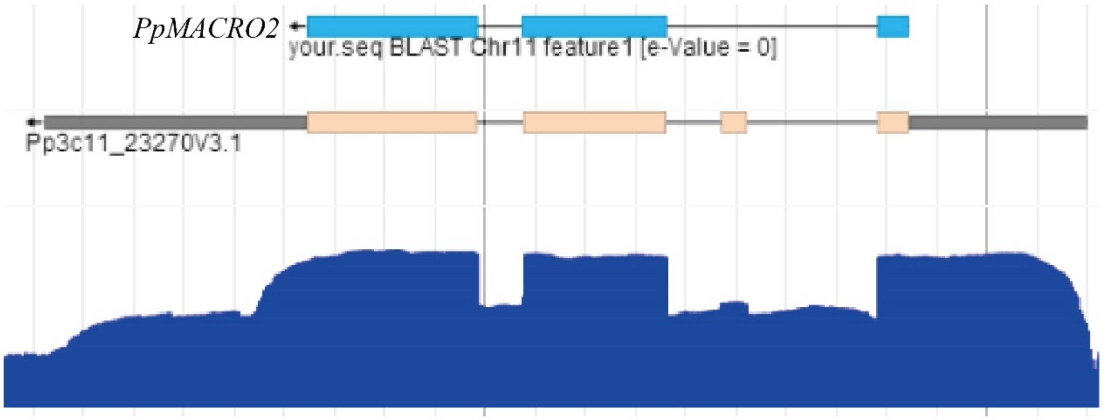

Supplementary Fig. 6. Coding sequence of *PpMACRO2*. Light blue boxes show the complete coding region of *PpMACRO2* that was amplified from *Physcomitrella patens* using RT-PCR. The sequencing result matched exons 1, 3 and 4 of *P. patens* genome annotation v3.3 in Phytozome (peach boxes), but exon 2 annotated by Phytozome was missing from our RT-PCR amplification.

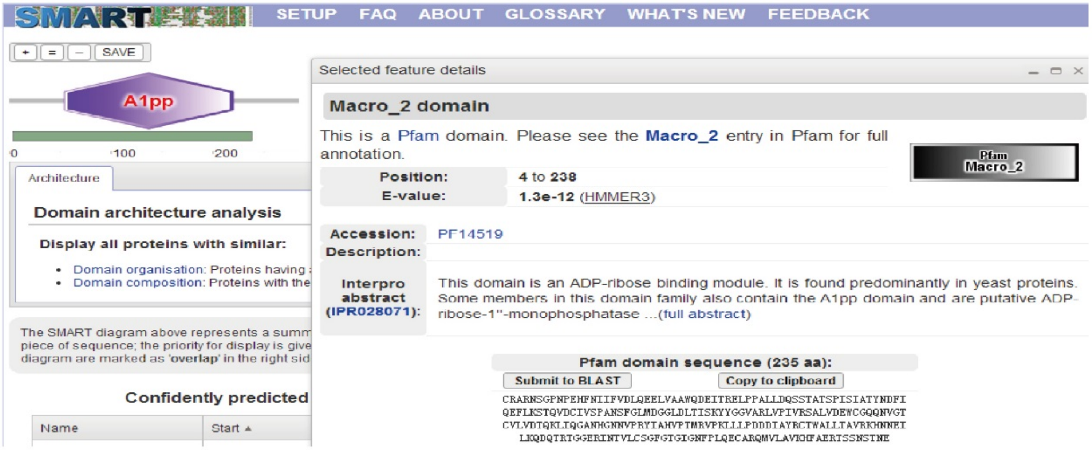

Supplementary Fig. 7. PpMACRO2 is predicted to be functionally related to ADP-ribosylation. PpMACRO2 sequence matchs the macro2 domain from the SMART database. The macro2 domain is annotated as containing an ADP-ribose binding module.

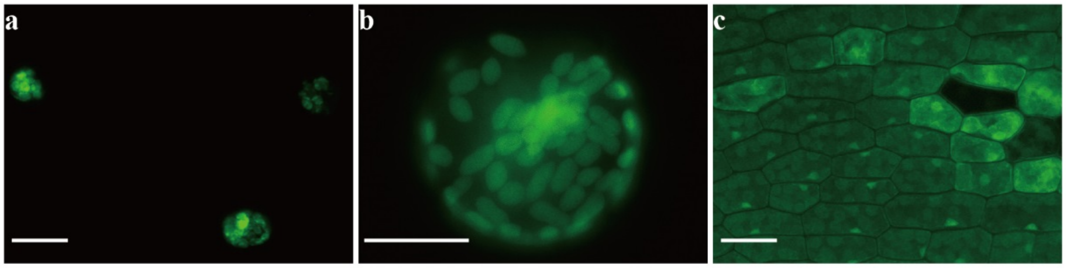

Supplementary Fig. 8. Subcellular localization of PpMACRO2. **a-b.** GFP signal of transient protoplast transformation. Enhanced green fluorescent protein (EGFP) was fused to *PpMACRO2* using pM999 vector for transient expression. GFP signal was observed in the nucleus and cytoplasm of protoplast. **c.** GFP signal of detached leaves. Detached leaves of *PpMACRO2*pro*:PpMACRO2-EGFP-GUS* lines were cultured on BCD medium for 48 hours, and GFP signal was observed in the nucleus and cytoplasm of detached leaves, indicating that PpMACRO2 is localized in both the nucleus and cytoplasm. Micrograph images shown were observed from at least three biological replicates. Scale bar: 100 µm in **a**, **c**; 25 µm in **b**.

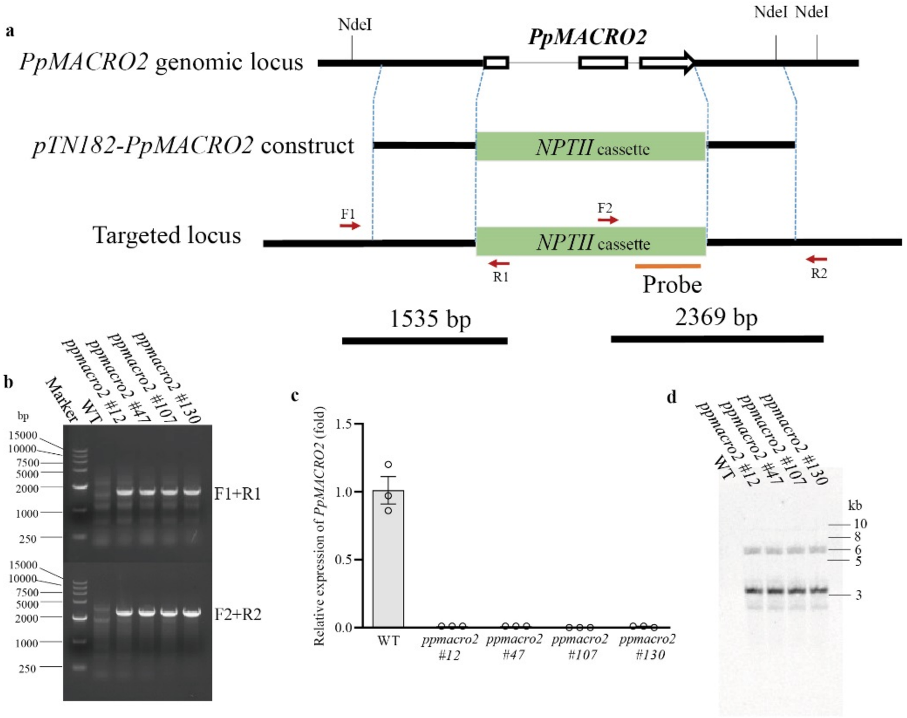

Supplementary Fig. 9. Generation and molecular identification of *PpMACRO2 ko* lines. **a.** Schematic diagram of *PpMACRO2 ko* construction and primers used for genotyping. White boxes denote the exons of *PpMACRO2*. The coding sequence of *PpMACRO2* was replaced by the *NPTII* cassette using homologous recombination. Red arrows indicate the positions of primers used for genomic PCR. Orange line indicates the position of probe used for Southern blotting analysis in *ko* plants. **b.** Results of genomic PCR for four *PpMACRO2 ko* lines using primers shown in the schematic diagram. The fragment length of genomic PCR is in line with expectation. **c.** Quantitative RT-PCR was performed to characterize four *PpMACRO2 ko* mutants. *PpMACRO2* transcription level was evaluated for the wild type and four *PpMACRO2 ko* lines using qRT-PCR. No expression of *PpMACRO2* was detected in the four *ko* mutants compared to the wild type. Three biological replications were performed, and *PpEF1a* was used as reference gene for normalization. Data show means ± s.e.m. **d.** Southern blotting analysis of *PpMACRO2 ko* lines. Genomic DNA of WT and *ko* (*#12*, *#47*, *#107* and *#130*) lines was digested with NdeI, and DIG-labeled fragment containing the *NPTII* gene was used as the probe. The result of Southern blotting indicates that *ko* lines resulted from single integration (~ 6 kb).

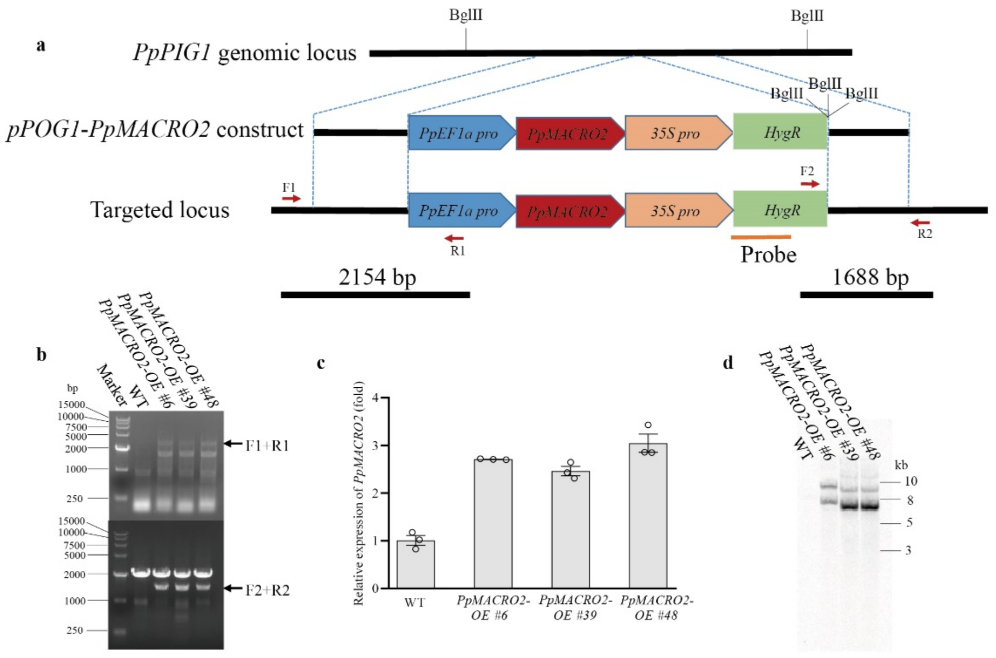

Supplementary Fig. 10. Generation and molecular identification of *PpMACRO2 OE* lines. **a.** Schematic diagram of *PpMACRO2 OE* construction and primers used for genotyping. Promoter *PpEF1a* was used for enhancing *PpMACRO2* expression in *PpPIG1* genomic locus, and hygromycin was used as selection marker and driven by 35S promoter. Red arrows indicate the positions of primers used for genomic PCR. Orange line indicates the position of probe used for Southern blotting analysis in *OE* plants. **b.** Genomic PCR confirmation of three *PpMACRO2 OE* lines using primers shown in the schematic diagram. The fragment length of genomic PCR is in accordance with expectation. **c.** Quantitative RT-PCR was used to characterize three *PpMACRO2 OE* lines. *PpMACRO2* transcription level was evaluated for the wild type and three *PpMACRO2 OE* lines using qRT-PCR. The expression level of *PpMACRO2* is over two-fold higher in *PpMACRO2 OE* lines than in the wild type. Three biological replications were performed and *PpEF1a* was used as reference gene for normalization. Data show means ± s.e.m. **d.** Southern blotting analysis of *PpMACRO2 OE* lines. Genomic DNA of WT and *OE* (*#6*, *#39* and *#48*) lines was digested with BglII, and DIG-labeled fragment containing the *HygR* gene was used as the probe. The result of Southern blotting indicates that *OE* lines resulted from single integration (~ 8 kb in *#6*, ~ 7.3 kb in *#39* and *#48*).

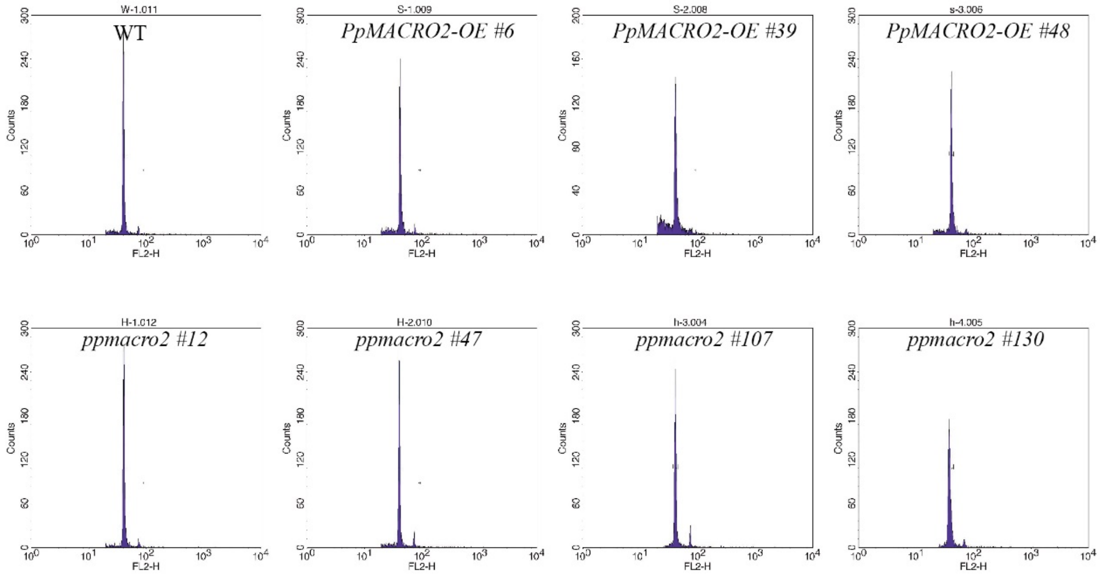

Supplementary Fig. 11. Chromosome ploidy analyses. WT, *ko* and *OE* plants were grown for one week on BCDAT medium, and their chromosome ploidy levels were determined using flow cytometry. The results indicate that chromosome ploidy levels of *ko* and *OE* plants are consistent with WT plants.

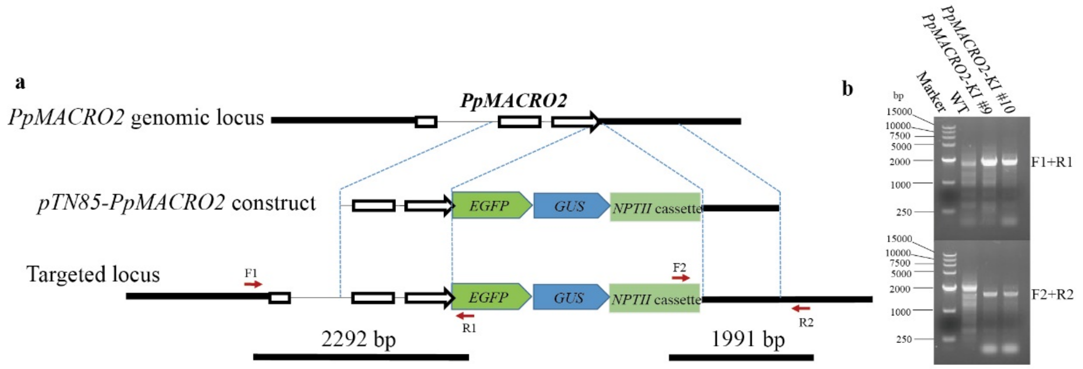

Supplementary Fig. 12. Schematic and genotyping for *PpMACRO2* knockin double tag lines. **a.** Construction of *PpMACRO2* knockin double tag lines. White boxes denote the exons of *PpMACRO2*, and red arrows indicate the positions of primers used for genomic PCR. **b.** Genomic PCR for *PpMACRO2* knockin double tag lines. The fragment length of genomic PCR is consistent with expectation as shown in the schematic.

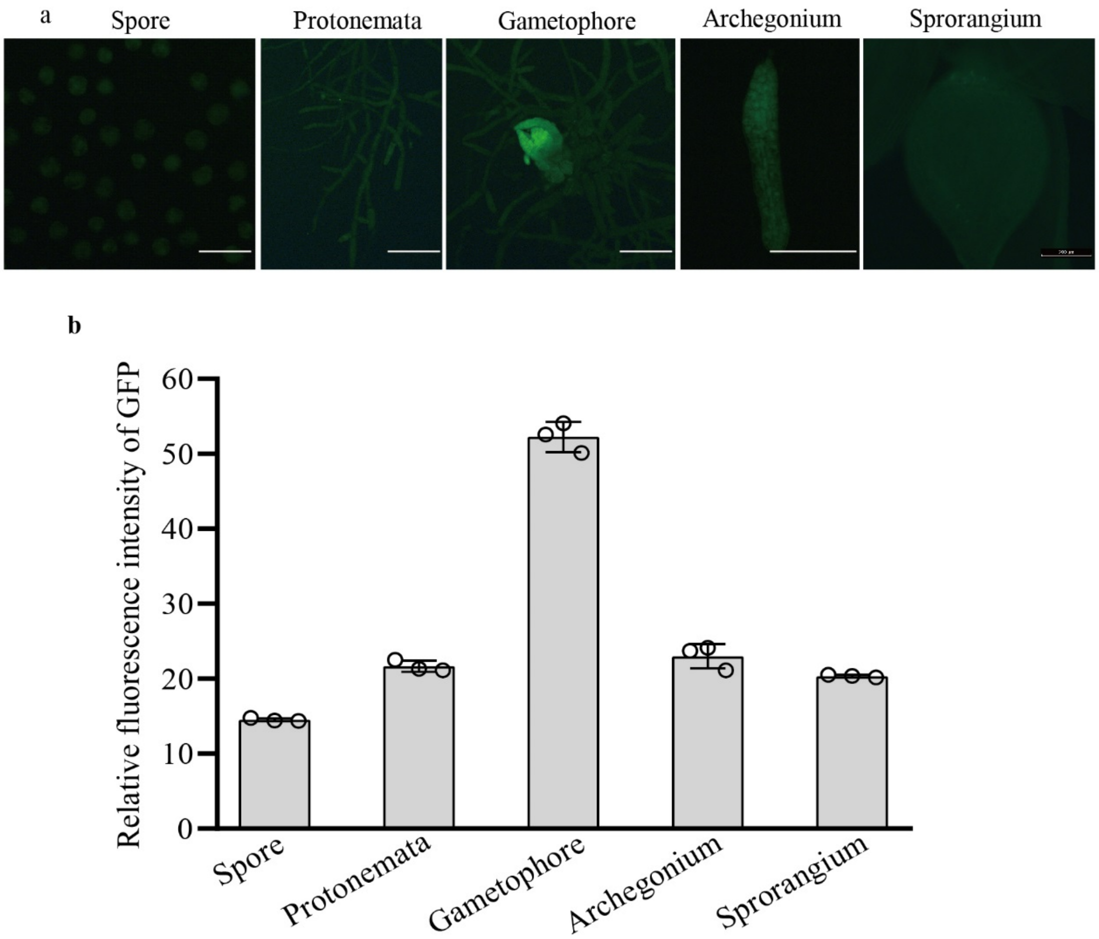

Supplementary Fig. 13. GFP signal in the life cycle of *P. patens*. **a.** Fluorescent signal was observed in spores, protonemata, gametophores, archegonia, and sporangia. **b.** Quantification of GFP fluorescence intensity. ImageJ was used for analyzing the relative intensity of GFP signal (in panel **a**). Micrograph images provided were observed from three biological replicates. Data show means ± s.e.m. of three biological replications. Scale bars: 200 µm.

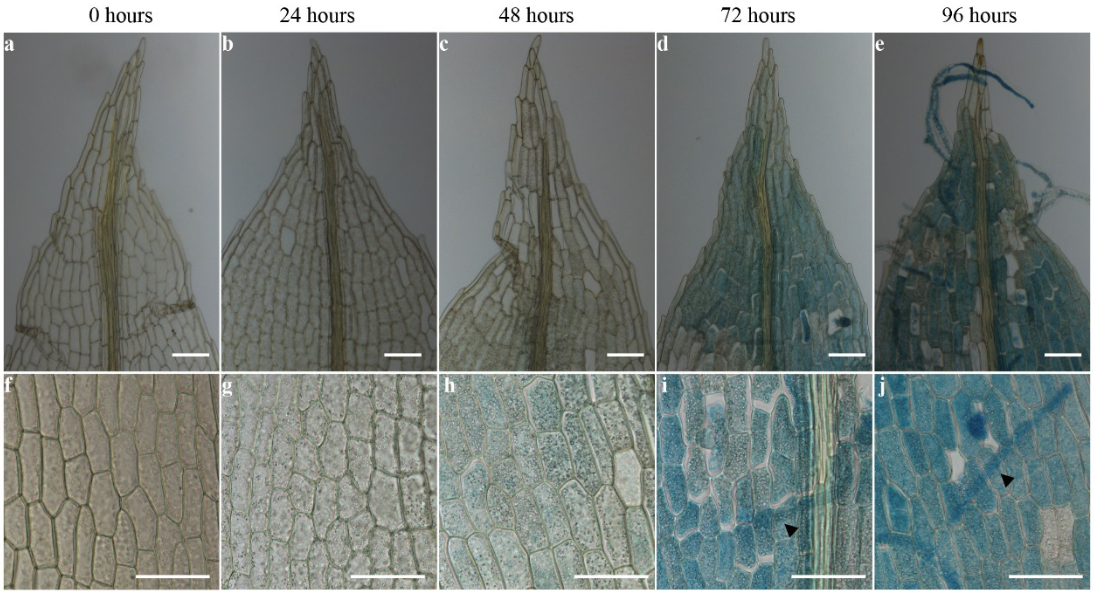

Supplementary Fig. 14. *PpMACRO2* expression was induced during tissue regeneration in detached leaves. GUS staining was examined after detached leaves were incubated on BCD medium for 24, 48, 72, and 96 hours, respectively. **a-e**. GUS staining of gametophore leaves. **f-j**. Local magnification for corresponding leaves. Arrowheads denote differentiated protonema in **i** and **j**. Micrograph images given were observed from ten biological replicates. Scale bar: 200 μm.

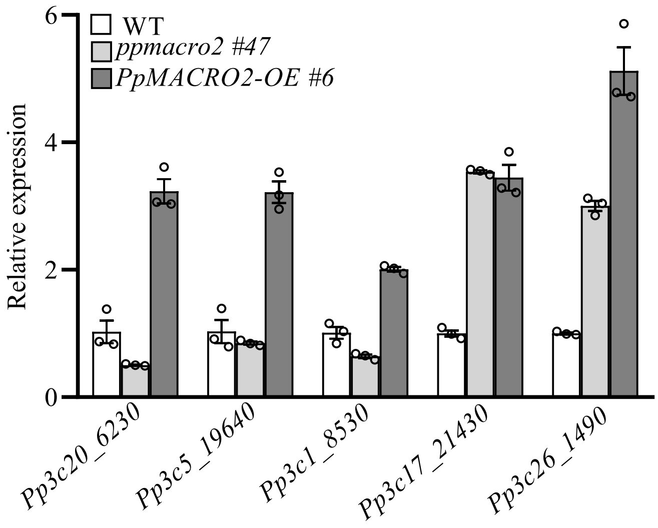

Supplementary Fig. 15. Expression level of *Pp3c20_6230*, *Pp3c5_19640*, *Pp3c1_8530*, *Pp3c17_21430* and *Pp3c26_1490* in WT, *ko* and *OE* plants. *Pp3c20_6230* encodes Sin-associated protein 30 (SAP30) that regulates histone deacetylation. The product of *Pp3c5_19640* is a homolog of methyl-CpG binding domain-containing protein 9 (MBD9) that modulates development by modifying chromatin structure. *Pp3c1_8530* encodes a SET domain protein that regulates histone methylation. The product of *Pp3c17_21430* is a basic-leucine zipper (bZIP) transcription factor that is involved in DNA binding. The expressed protein of *Pp3c26_1490* is related to Dof domain, including a zinc finger DNA-binding domain. Transcript abundance of these genes was confirmed through qRT-PCR with three independent biological replicates, normalized to *PpEF1a*. Data show means ± s.e.m.

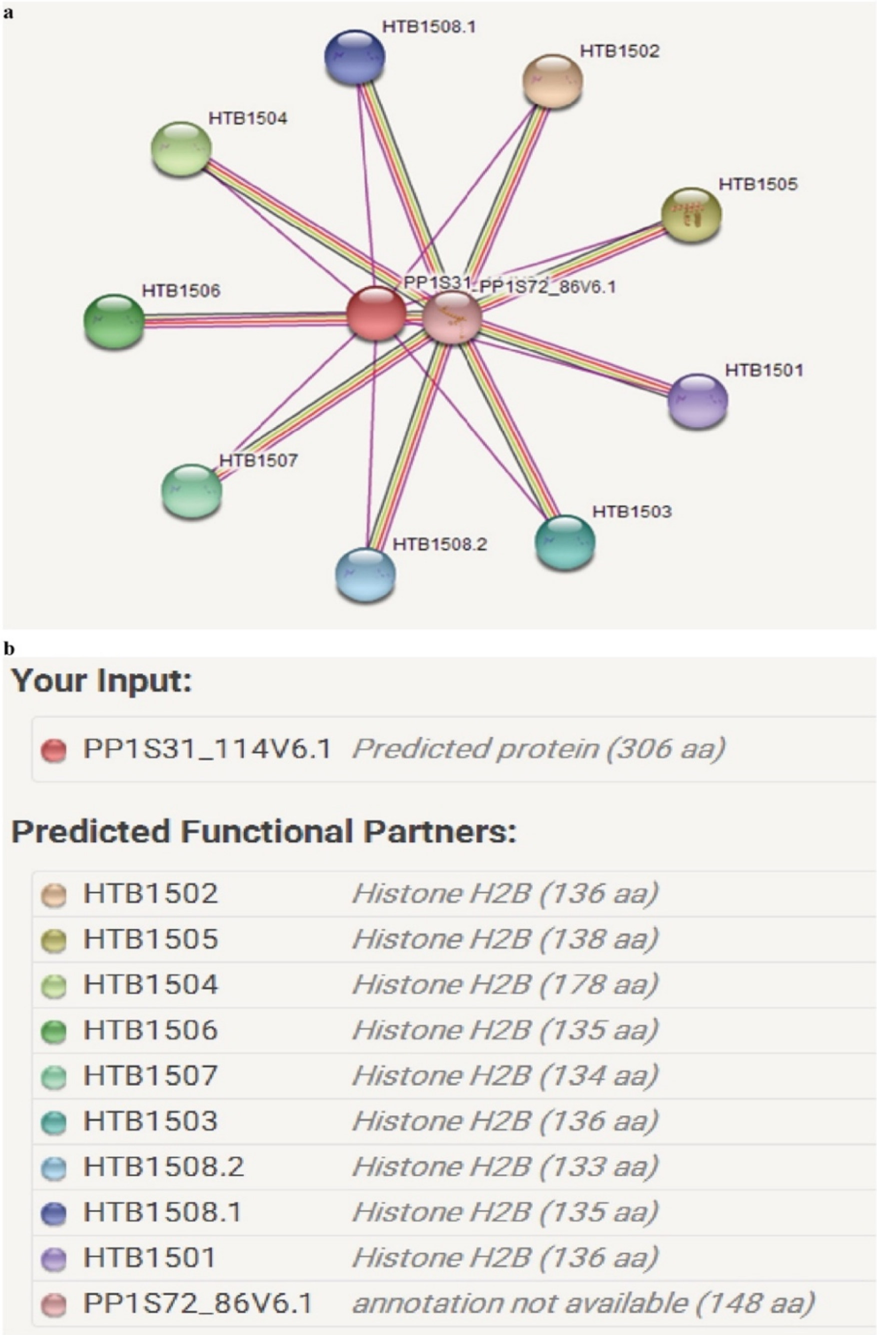

Supplementary Fig. 16. The interaction proteins of PpMACRO2. PpMACRO2 is predicted to interact with histones H2A and H2B according to the STRING database. PP1S31_114V6.1 indicates PpMACRO2, PP1S72_86V6.1 represents histones H2A, and other HTBs refer to histones H2B.

Supplementary Table 1. Primers used for genomic PCR of *PpMACRO2* homologous sequences and ITS2 in *Spirogyra sp*.

Primer	Sequence (5'-3')
S. pratensis-F	CATCCCAAAGTCGAGCTCGTT
S. pratensis-R1	ACATGCATTATTAGGGTGGG
S. pratensis-R2	GCCATTAATCTTGCAGCATC
PpMACRO2 Homolog-F1	CGTTTGGACTCATGGATGGG
PpMACRO2 Homolog-R1	GCGAACTGCAGCCAACAT
PpMACRO2 Homolog-F2	ATGTTGGCTGCAGTTCGC
PpMACRO2 Homolog-R2	ATGCTTAAATGCAAGCGCCAT
PpMACRO2 Homolog-F3	TTTGACTGCATCGTGAGCCC
PpMACRO2 Homolog-R3	TTGAAATGGCCAAATCAATGCC
ITS2-F	TGCACTCTGCGCAAGCGGAGTAT
ITS2-R	GGCCTTGTCTGATCTGAGGTC

Supplementary Table 2. Genes related to cell wall formation and cell division are often down-regulated in *ko* and *OE* plants. Data were generated from RNA-seq data and verified through qRT-PCR.

Gene ID	*ppmacro2 #47*			*PpMACRO2-OE #6*			Gene description
Pp3c9_3880	-2.53	-5.48	-1.52	-1.09	-1.02	-0.51	PF06955:Xyloglucan endo-transglycosylase (XET) C-terminus|PF00722:Glycosyl hydrolases family 16
Pp3c24_13310	-1.64	-2.12	-0.12	-0.47	-0.51	-0.10	PF00722:Glycosyl hydrolases family 16|PF06955:Xyloglucan endo-transglycosylase (XET) C-terminus
Pp3c6_480	-1.96	-6.86	-2.14	-1.82	-1.68	-1.06	PF06955:Xyloglucan endo-transglycosylase (XET) C-terminus|PF00722:Glycosyl hydrolases family 16
Pp3c26_9030	-2.71	-3.07	-2.00	-1.43	-1.12	-0.82	PF14543:Xylanase inhibitor N-terminal|PF14541:Xylanase inhibitor C-terminal
Pp3c5_23400	-2.86	-3.49	-2.01	-1.29	-1.05	-0.89	PF01095:Pectinesterase
Pp3c25_6620	-3.34	-2.48	-1.94	-1.42	-1.32	-0.83	PF03016:Exostosin family
Pp3c13_12000	-5.07	-5.21	-1.92	-3.13	1.14	0.04	PF00150:Cellulase (glycosyl hydrolase family 5)
Pp3c8_9790	-4.87	-7.45	-7.50	-3.32	-6.54	-3.39	Encodes a tetratricopeptide repeat protein required for cell cycle exit after meiosis II.
Pp3c24_8590	-0.56	-0.50	0.27	0.41	0.13	-0.17	PF12214:Cell cycle regulated microtubule associated protein
Pp3c24_19380	-0.60	-0.72	-0.70	-0.77	-0.75	-1.37	PF00307:Calponin homology (CH) domain|PF16796:Microtubule binding|PF00225:Kinesin motor domain
Pp3c6_7500	-0.92	-1.04	-1.15	-0.61	-0.75	-1.09	PF03953:Tubulin C-terminal domain|PF00091:Tubulin/FtsZ family, GTPase domain
Pp3c7_15580	-1.75	-2.02	-0.31	-1.61	-1.59	-1.22	PF03999:Microtubule associated protein (MAP65/ASE1 family)
Pp3c21_8330	-3.26	-1.39	-3.43	1.08	-0.12	-1.26	PTHR22844//PTHR22844:SF125 - F-box and WD40 domain protein
Pp3c9_7690	-1.98	-2.85	-1.94	-0.35	-0.14	0.00	PF00646//PF01344 - F-box domain (F-box) // Kelch motif (Kelch_1)

Supplementary Table 3. Additional gene information for Fig. 6 of the main text. Gene identifiers are from Phytozome. These genes are related to epigenetic modification and developmental transcription factors.

Gene ID	Gene description
Pp3c20_13850	PF08241:Methyltransferase domain
Pp3c3_26550	PF00850:Histone deacetylase domain
Pp3c17_14770	PF00856:SET domain protein |TPR repeat-containing protein
Pp3c13_4470	PF00856:SET domain protein |Histone-lysine N-methyltransferase
Pp3c13_19810	PF00856:SET domain protein |Histone-lysine N-methyltransferase ATX4-related
Pp3c7_2300	PF00847:AP2 domain |Any process that modulates the frequency, rate or extent of cellular DNA-templated transcription
Pp3c10_20000	PF00847:AP2 domain |Ethylene-responsive transcription factor 15-related
Pp3c7_10780	PF00847:AP2 domain |Any process that modulates the frequency, rate or extent of cellular DNA-templated transcription
Pp3c4_2530	PF00847:AP2 domain |Any process that modulates the frequency, rate or extent of cellular DNA-templated transcription
Pp3c3_6830	PF00847:AP2 domain |Any process that modulates the frequency, rate or extent of cellular DNA-templated transcription
Pp3c14_3280	PF00847:AP2 domain |Ethylene-responsive transcription factor CRF1-related
Pp3c3_6420	PF00847:AP2 domain |Any process that modulates the frequency, rate or extent of cellular DNA-templated transcription
Pp3c22_20520	PF00847:AP2 domain |Any process that modulates the frequency, rate or extent of cellular DNA-templated transcription
Pp3c1_14230	PF00847:AP2 domain |Any process that modulates the frequency, rate or extent of cellular DNA-templated transcription
Pp3c8_7340	PF00847:AP2 domain |Any process that modulates the frequency, rate or extent of cellular DNA-templated transcription
Pp3c1_5010	PF00847:AP2 domain |Any process that modulates the frequency, rate or extent of cellular DNA-templated transcription
Pp3c11_10660	PF00847:AP2 domain |Any process that modulates the frequency, rate or extent of cellular DNA-templated transcription
Pp3c15_3620	PF00847:AP2 domain |Any process that modulates the frequency, rate or extent of cellular DNA-templated transcription
Pp3c17_18130	PF0046:Homeobox domain |Homeobox associated leucine zipper
Pp3c6_2730	PF0046:Homeobox domain |Transcription factor HEX, contains HOX and HALZ domains
Pp3c1_24020	PF0046:Homeobox domain |Homeobox-leucine zipper protein HDG2
Pp3c12_21760	PF0046:Homeobox domain |Homeobox associated leucine zipper
Pp3c15_13310	PF0046:Homeobox domain|SF290
Pp3c3_37710	PF0046:Homeobox domain| Transcription factor PHOX2/ARIX, contains HOX domain

Supplementary Table 4. Primers used for vector construction of transient expression, *KI*, *ko* and *OE* of *PpMACRO2*.

Primer	Sequence (5'-3')
pM999-PpMACRO2-F(EcoRI)	TCCTGAAACTCCCTCGAATTCATGAAGTTGAATGTCGTGC
pM999-PpMACRO2-R(SacI)	ATCTCCTTGGGCATCGAGCTCCTTCCCGAGGACCTCGAA
pTN85-PpMACRO2-KI-5F(KpnI)	GAACAAAAGCTGGGTACCTTTCCAGAACCCTTCGTAGAGT
pTN85-PpMACRO2-KI-5R(XhoI)	GCTCACGTCGACCTCGAGGACCTCGAAAGTACACGGAGA
pTN85-PpMACRO2-KI-3F(BmaHI)	CGGGGATCGGGGGGATCCTCCTGGACATTTGATTCACTGG
pTN85-PpMACRO2-KI-3R(XbaI)	GGTGGCGGCCGCTCTAGAAAGGGGAGAGCACTTCTACTATC
pTN182-PpMACRO2-ko-5F(SalI)	CCCCCCCTCGAGGTCGAC AACTTGCAATGACGACGAG
pTN182-PpMACRO2-ko-5R(HindIII)	GAATTCGATATCAAGCTT ACAGCCAATTGAACAAACTCC
pTN182-PpMACRO2-ko-3F(SmaI)	GGGATCGCATGCCCGGGGCTCGGTCAGGTTAATTTCTG
pTN182-PpMACRO2-ko-3R(BmaHI)	CGGCCGCTCTAGGATCC AGTTCCAATTTTGAAAGCTGG
pPOG1-PpMACRO2-OE-F(NotI)	TCCAGTCACTATGGCGGCCGCATGAAGTTGAATGTCGTGC
pPOG1-PpMACRO2-OE-R(SalI)	TATCCAGTCACTATGGTCGACCTTCCCGAGGACCTCGAA

Supplementary Table 5. Primers used for genotyping of *KI*, *ko* and *OE* plants of *PpMACRO2*.

Primer	Sequence (5'-3')
PpMACRO2-KI-F1	CGCACTGGAGTATCTCGTTC
PpMACRO2-KI-R1	ATGCCGTTCTTCTGCTTGTC
PpMACRO2-KI-F2	ACGAGACGACTAAACCTGGA
PpMACRO2-KI-R2	TGAGAGATTACGGGAGCACT
PpMACRO2-ko-F1	GTGGCAAGAGAGAAGGCTAA
PpMACRO2-ko-R1	TGTCTGTTGTGCCCAGTCAT
PpMACRO2-ko-F2	CTTGGGTGGAGAGGCTATTC
PpMACRO2-ko-R2	GAAGGTTGAGAGATTACGGG
PpMACRO2-OE-F1	CTCCTCCAAGCATCCACCCTA
PpMACRO2-OE-R1	CTGTCATGTCCCCTCGATAT
PpMACRO2-OE-F2	CCTATACCCCCTAATAACCCC
PpMACRO2-OE-R2	CTTCACTCATCCACATCCAA

Supplementary Table 6. Primers used for qRT-PCR of *PpMACRO2* and genes related to epigenetic modification, AP2 domain and homeobox.

Gene ID	Primer-F (5'-3')	Primer-R (5'-3')
PpMACRO2	TCAAGAGGAATTAGTGGCAGC	AGTCGCTATTGAGATGGGCG
Pp3c20_13850	CTTGGACGACCTCAAGCAGT	AGAGGCTCGCAACCATTCAA
Pp3c3_26550	CCTAATGTAAGCCTCGCGGT	CTGCTGGAATGGTGGTGGAT
Pp3c17_14770	GTACGGAAGCAGTTGAGCCT	GGTTGCCTAGCACTCTCAGG
Pp3c1_8530	CAGCAAGGAAGTCAGGAGGG	GCGACAGGATAAGATGCGGA
Pp3c13_4470	GATCTCCGGGTGGAAATGGA	ACTGATGCTGTCTTGCACCT
Pp3c13_19810	CTCTTGCGAACCATTCCGAT	TAATCCCTTCTTCTGCCGCC
Pp3c17_21430	CCGAGATGGATGGTGTAGCTC	ACGCAATTGGGCAGTCAAAA
Pp3c26_1490	TTGTCATCGAGAGTGCCACC	GCGCCGTTGAAGTAACCAAG
Pp3c3_6830	GTGAATGCAATGGTGGCGAG	TGGGGACTGCAACATTCACA
Pp3c7_2300	TGCCCAGTTCCGTGAATCAA	TCCTGATCGAACCCTGTCCT
Pp3c10_20000	TAGACCAACCACAAGGCCAC	AGGTCTGCGATTTCCACGAG
Pp3c7_10780	TGGCTTGTGATACGCAGGTG	ATTTTTCGTGTGGGACGGCA
Pp3c3_6420	TCAGCTAGCAGTTTCAGCCC	AGATGCATCGACCTGGAACC
Pp3c4_2530	AAAGATCCCGAGCACACGTT	GGTCTGCGGTAGAGGATTCG
Pp3c14_3280	ACTTCATGATGGACTGGGCG	TCCCCCAAAAACTCCAACCC
Pp3c1_14230	ACTCACTCACCATGTCGTGC	TGTCTTGAATGTCCCCAGCC
Pp3c22_20520	CAGGGGTTGCTTCTACCGAG	TCCTCTCCCCTTCCTGACTG
Pp3c8_7340	AAACTCTGGTACTTCGGCGG	CCAGGCCAATCCCACATCTT
Pp3c12_21760	CCCGAACCACCGATAGTAGC	TTCACGGTGACGTCGTGTAG
Pp3c6_2730	CAATGTCACGCTTGTGCGAA	GGTCACTGCATCCTCTAGGC
Pp3c17_18130	CAAGGATCACCACCACACGA	CCTGAGCTTGTCGACGTCTT
Pp3c1_24020	CGGAGGAGGAGGAGCAAAG	TGAGGAACGCTCAAGACCAC
Pp3c3_37710	TTCGTTTGCGGCAATGATCG	CTGTCCACCTCCTTGCGATT
Pp3c15_13310	TGAGCTTGCGGAGAACAAGA	TGGGCTTGTGTACGCTCAAA

Supplementary Table 7. Primers used for probe amplification to detect single integration in *ko* and *OE* lines.

Primer	Sequence (5'-3')
PpMACRO2-ko-probe-F	GCCGAGAAAGTATCCATCAT
PpMACRO2-ko-probe-R	TCAGAAGAACTCGTCAAGAA
PpMACRO2-OE-probe-F	AGGGCGAAGAATCTCGTGCT
PpMACRO2-OE-probe-R	TTGGCGACCTCGTATTGGGA
