# Supplementary material for: A mycorrhizae-like gene regulates stem cell and gametophore development in mosses
Source: Nat Commun. 2020 Apr 24;11:2030. doi: 10.1038/s41467-020-15967-6 (PMC7181705; doi:10.1038/s41467-020-15967-6)
Supplement: Supplementary file 4 — Source Data [file 41467_2020_15967_MOESM4_ESM.zip › Raw data/Uncropped blot and gel images.docx]

Uncropped gel and southern blot images for corresponding Supplementary Figures.





Uncropped gel image for Supplementary Figure 3.





Uncropped gel image for Supplementary Figure 9b.


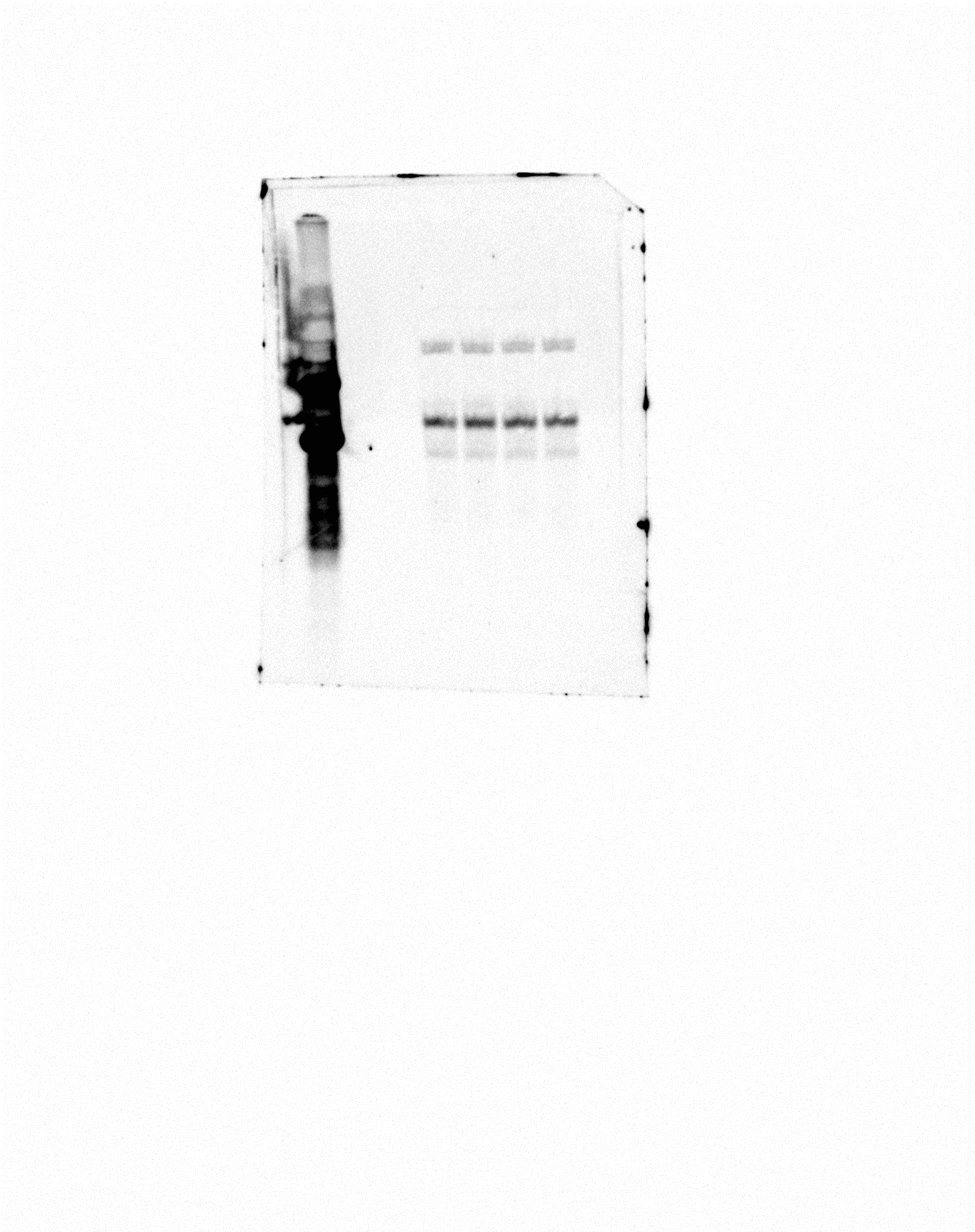


Uncropped southern blot image for Supplementary Figure 9d.





Uncropped gel image for Supplementary Figure 10b.





Uncropped gel image for Supplementary Figure 10b.


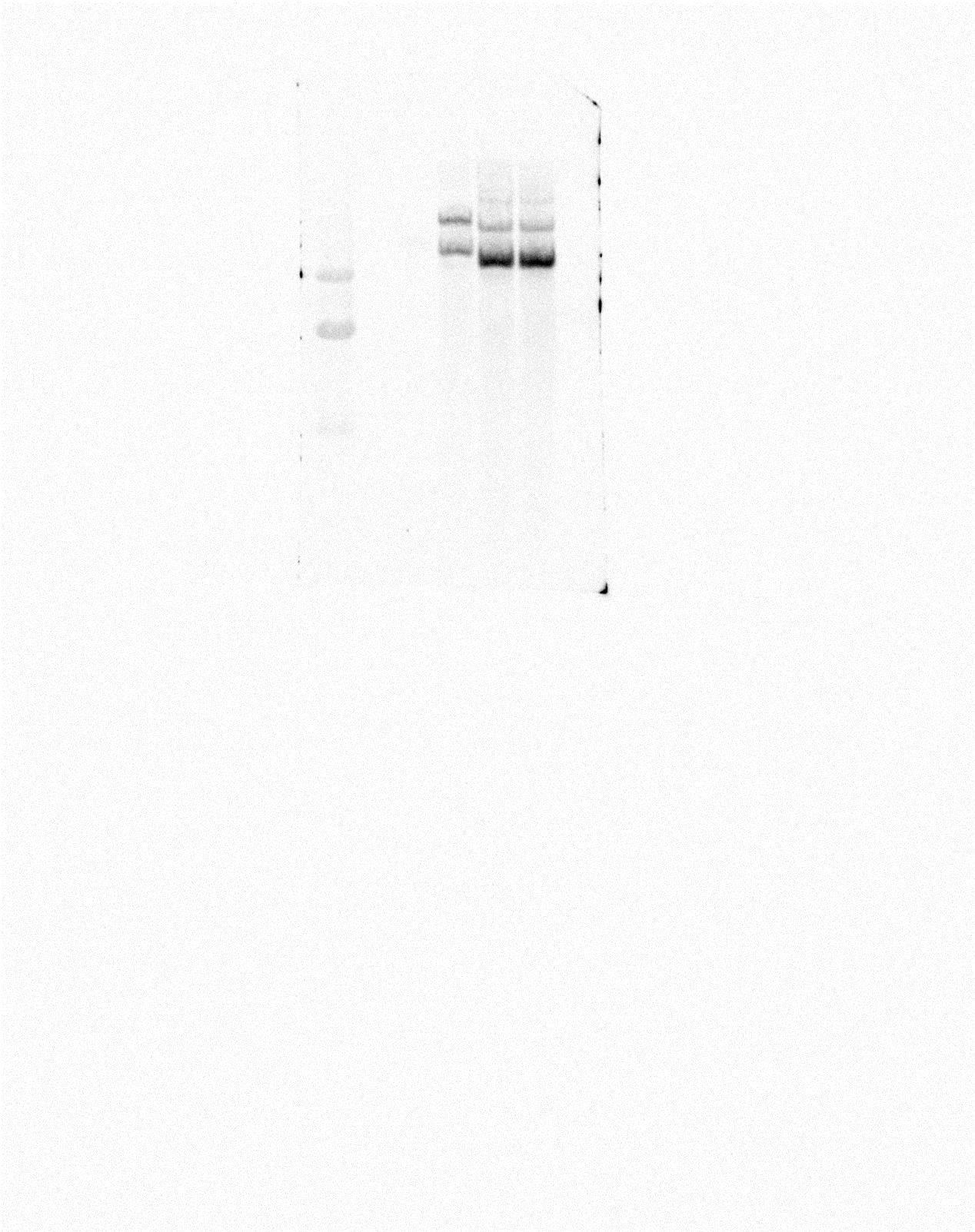


Uncropped southern blot image for Supplementary Figure 10d.





Uncropped gel image for Supplementary Figure 12b.
