# Supplementary figures and images for: A mycorrhizae-like gene regulates stem cell and gametophore development in mosses
Source: Nat Commun. 2020 Apr 24;11:2030. doi: 10.1038/s41467-020-15967-6 (PMC7181705; doi:10.1038/s41467-020-15967-6)

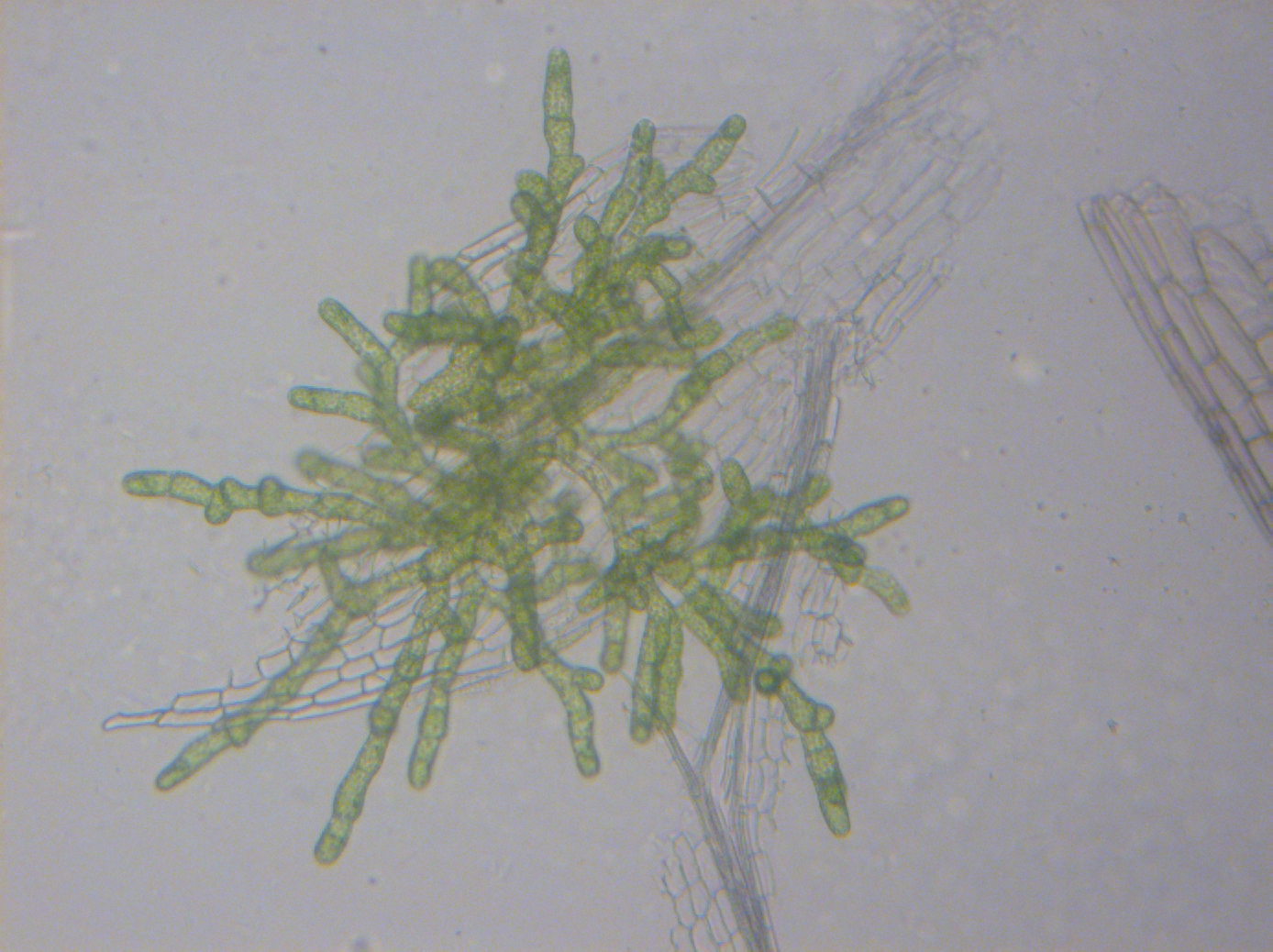

Supplement: Supplementary file 4 — Source Data [file 41467_2020_15967_MOESM4_ESM.zip › Raw data/Raw data for Figures/Figure 2a PpMACRO2-OE #6 19d.jpg]

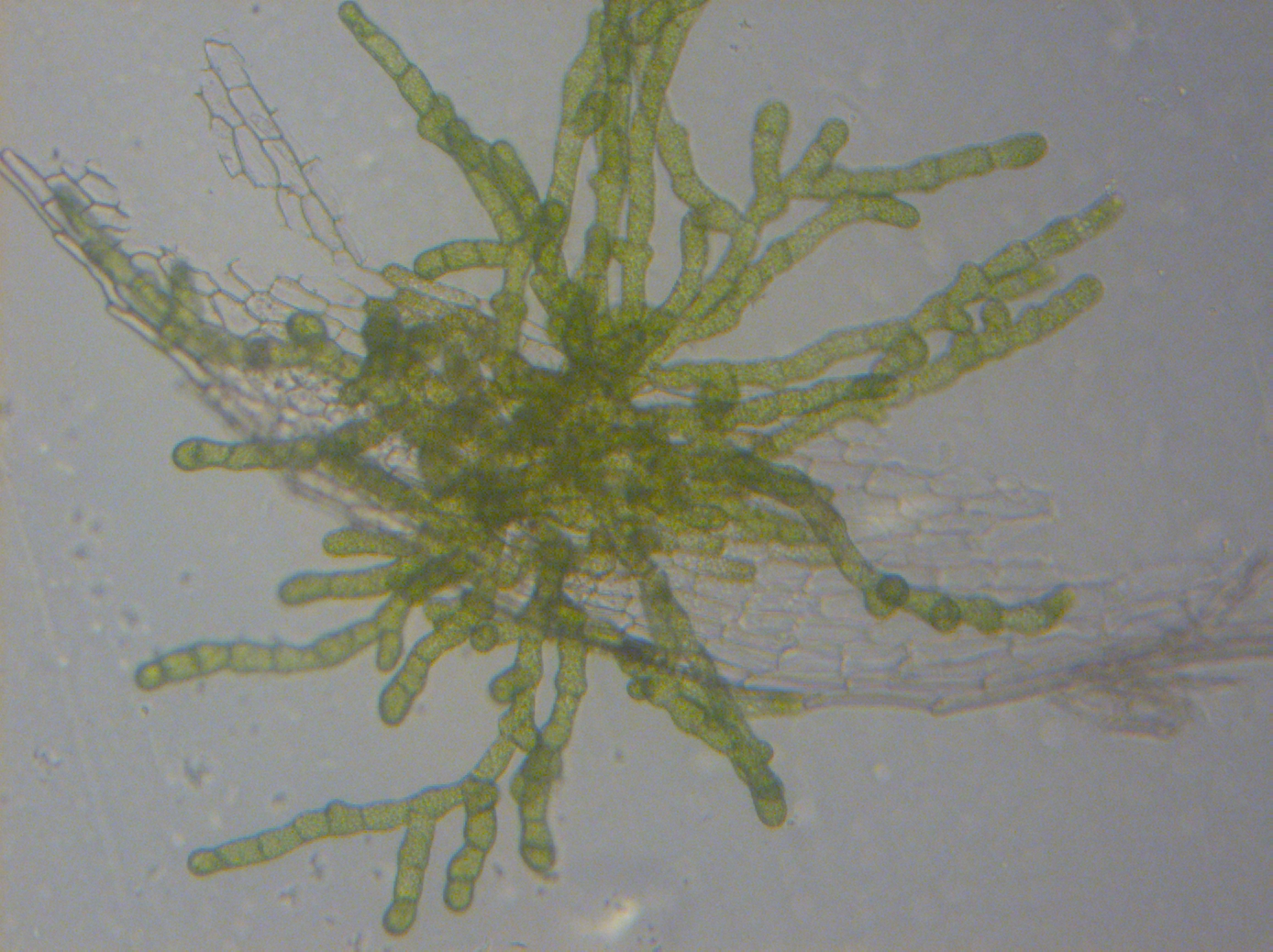

Supplement: Supplementary file 4 — Source Data [file 41467_2020_15967_MOESM4_ESM.zip › Raw data/Raw data for Figures/Figure 2a PpMACRO2-OE #6 30d.jpg]

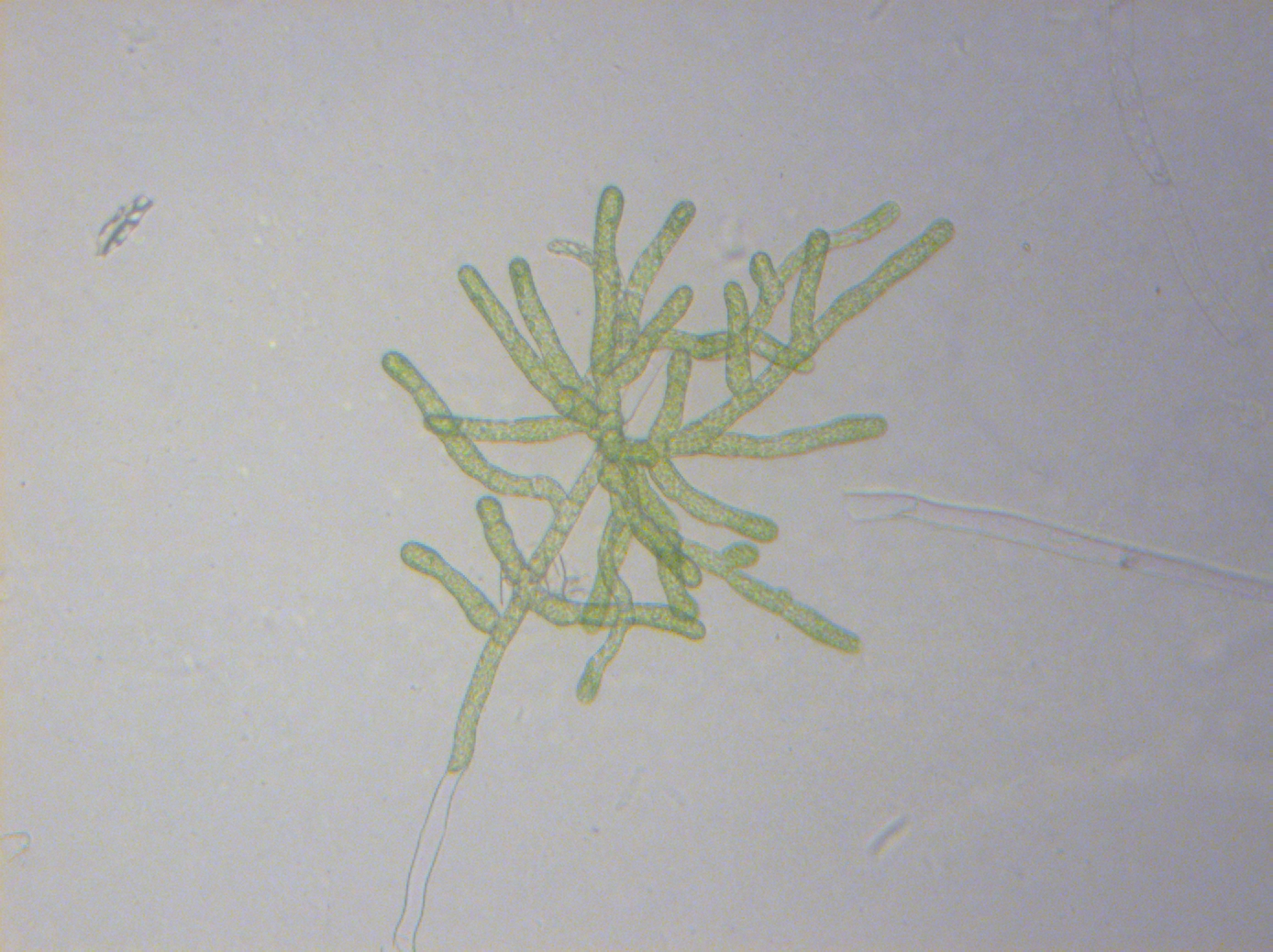

Supplement: Supplementary file 4 — Source Data [file 41467_2020_15967_MOESM4_ESM.zip › Raw data/Raw data for Figures/Figure 2a WT 19d.jpg]

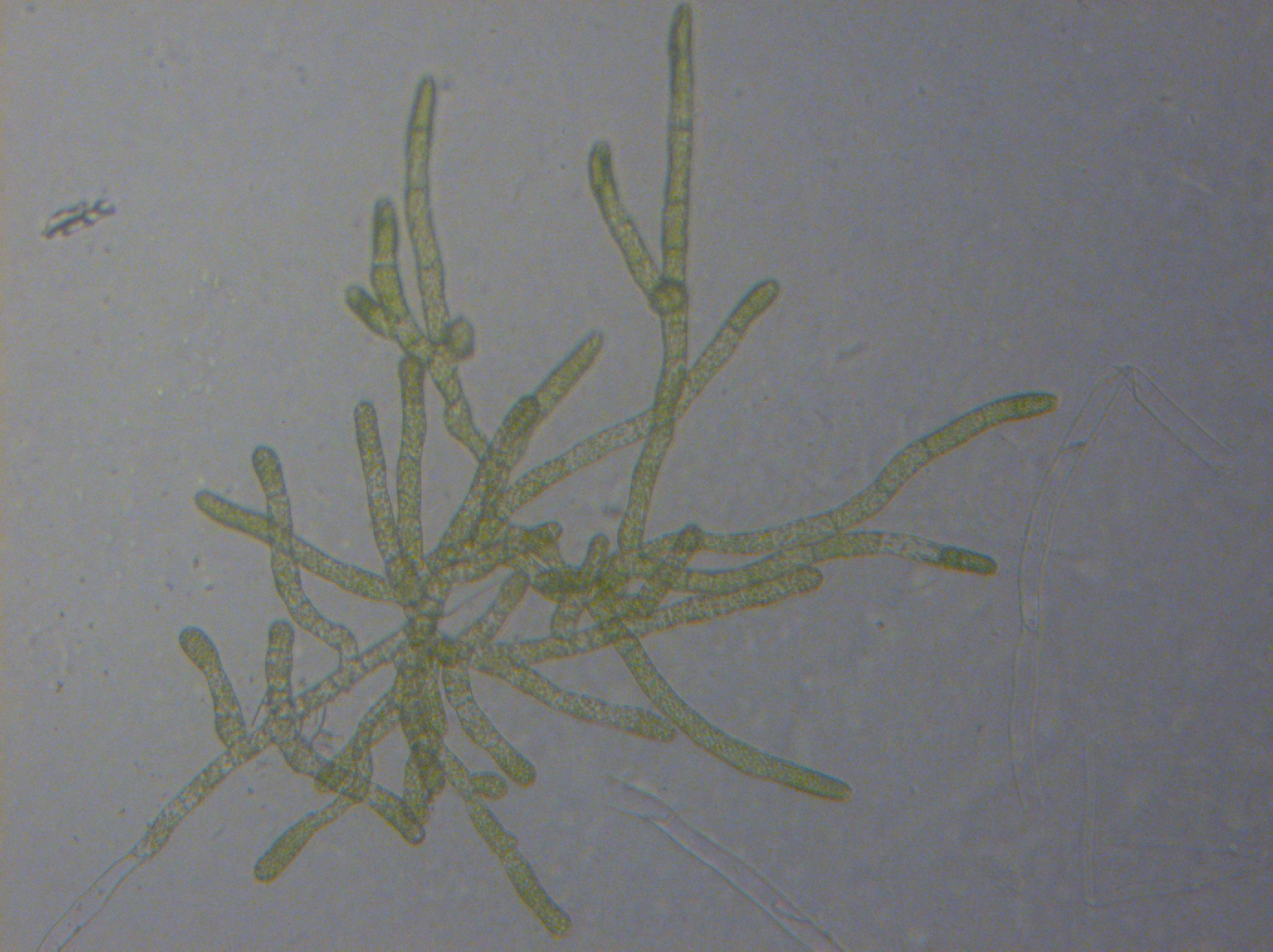

Supplement: Supplementary file 4 — Source Data [file 41467_2020_15967_MOESM4_ESM.zip › Raw data/Raw data for Figures/Figure 2a WT 30d.jpg]

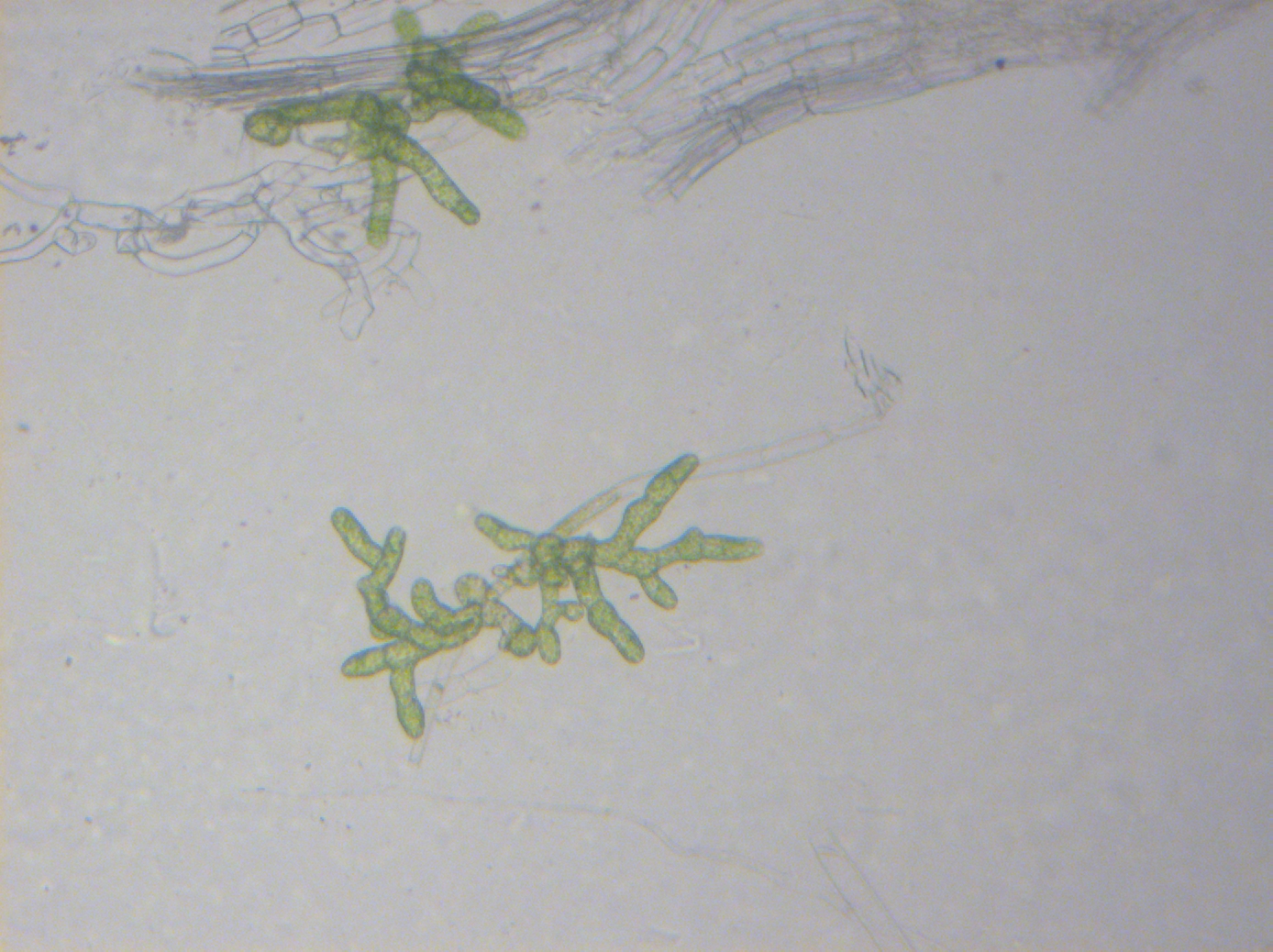

Supplement: Supplementary file 4 — Source Data [file 41467_2020_15967_MOESM4_ESM.zip › Raw data/Raw data for Figures/Figure 2a ppmacro2 #47 19d.jpg]

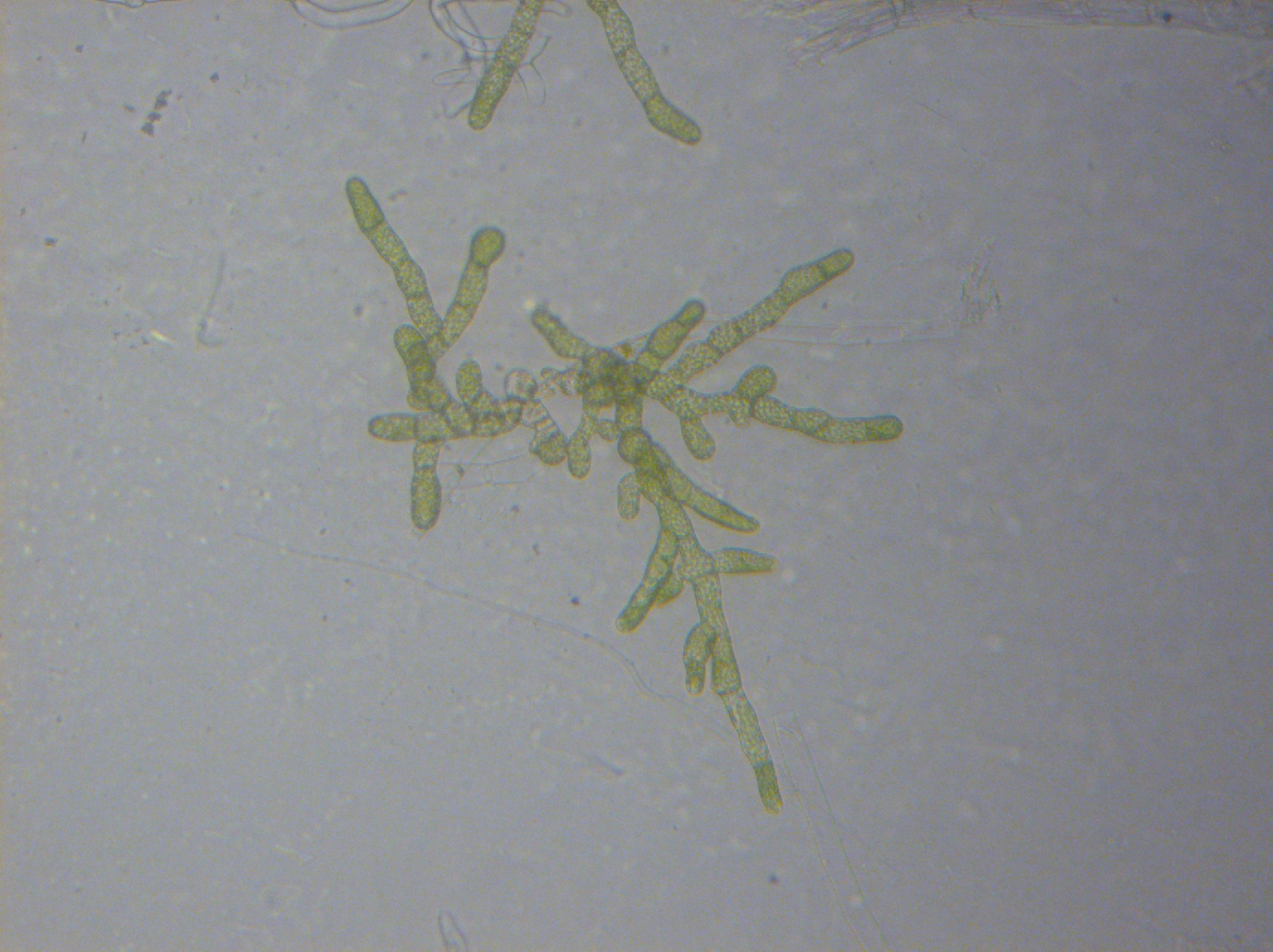

Supplement: Supplementary file 4 — Source Data [file 41467_2020_15967_MOESM4_ESM.zip › Raw data/Raw data for Figures/Figure 2a ppmacro2 #47 30d.jpg]

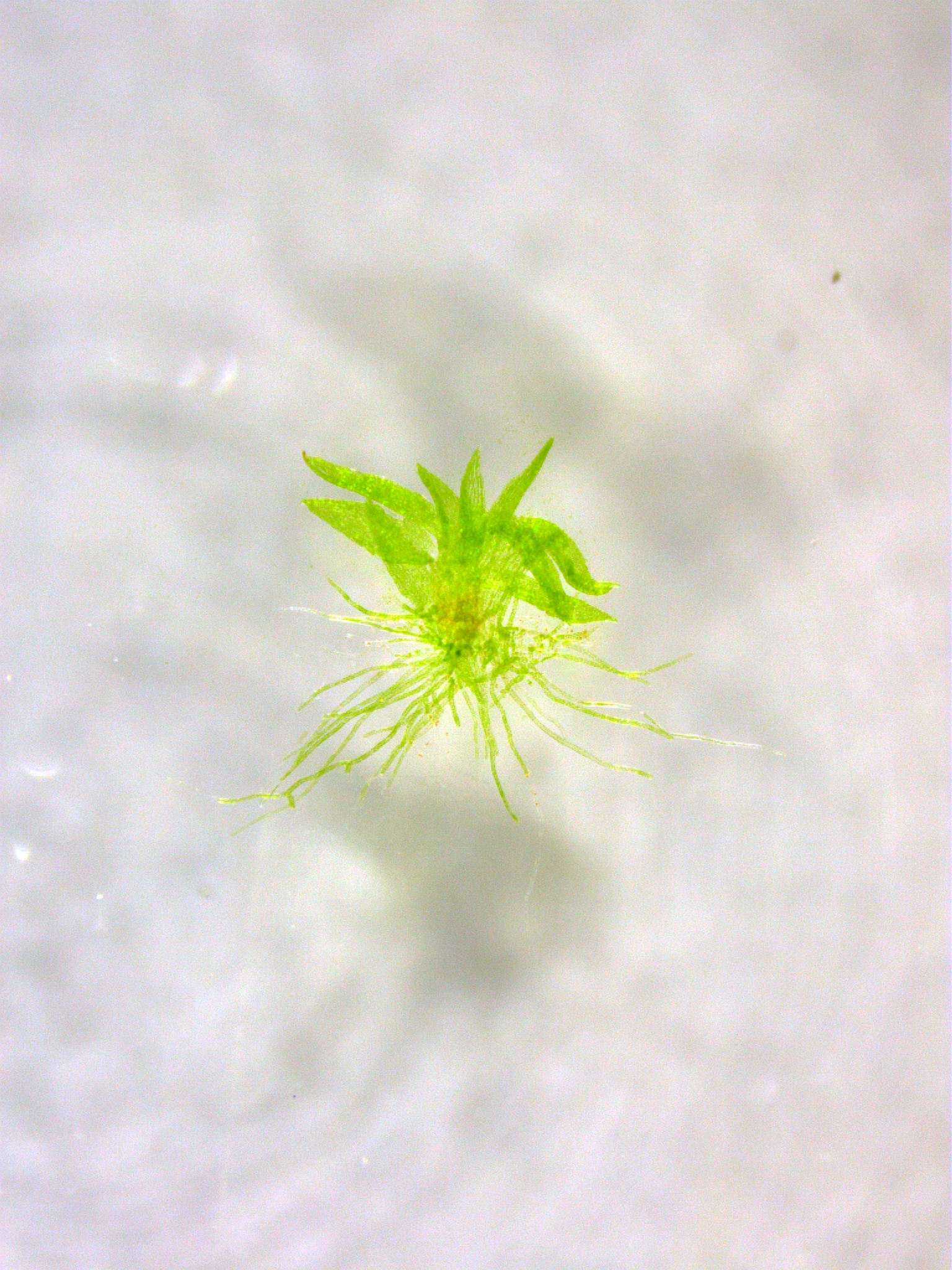

Supplement: Supplementary file 4 — Source Data [file 41467_2020_15967_MOESM4_ESM.zip › Raw data/Raw data for Figures/Figure 2c PpMACRO2-OE #6.tif]

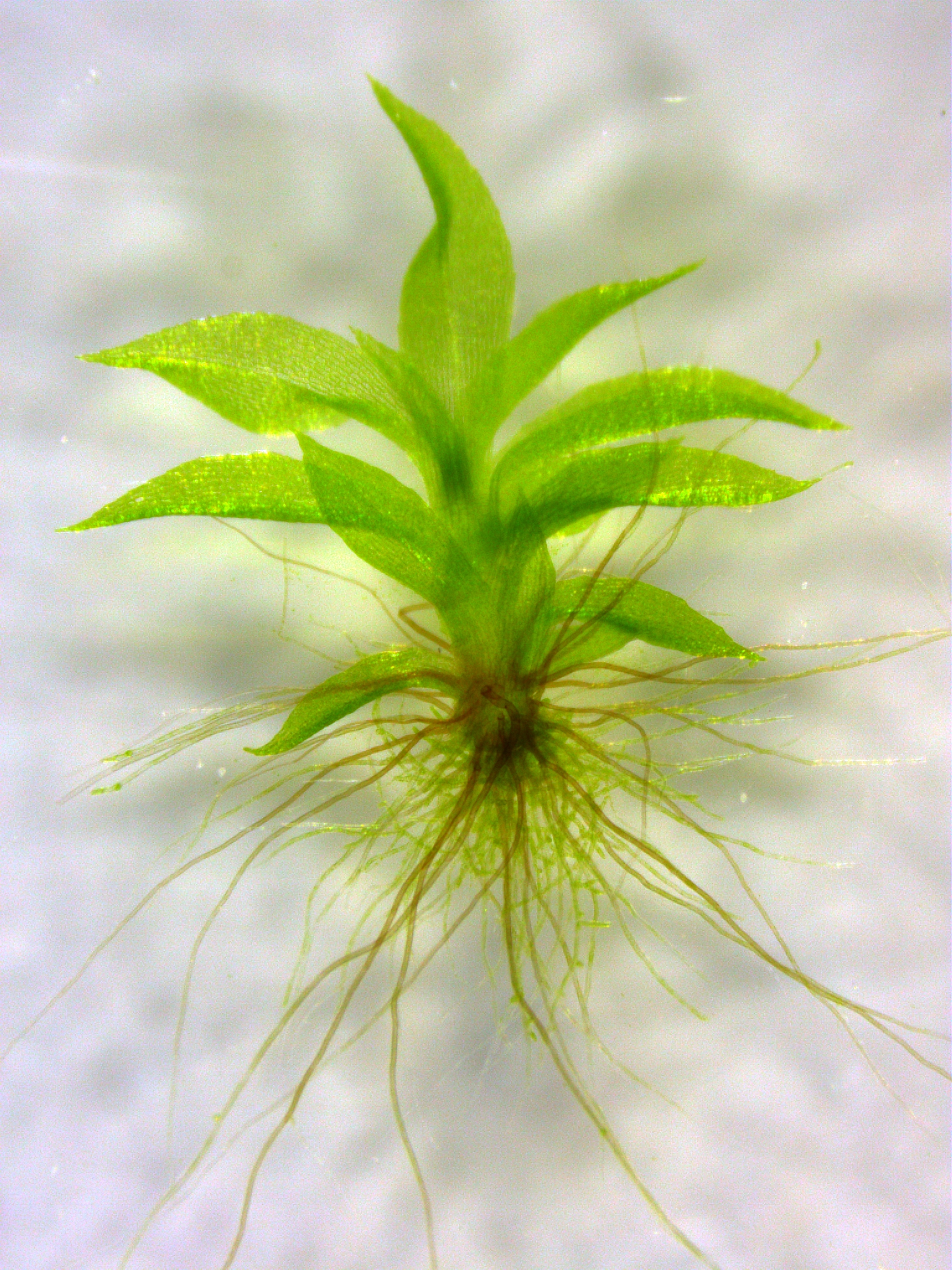

Supplement: Supplementary file 4 — Source Data [file 41467_2020_15967_MOESM4_ESM.zip › Raw data/Raw data for Figures/Figure 2c WT.tif]

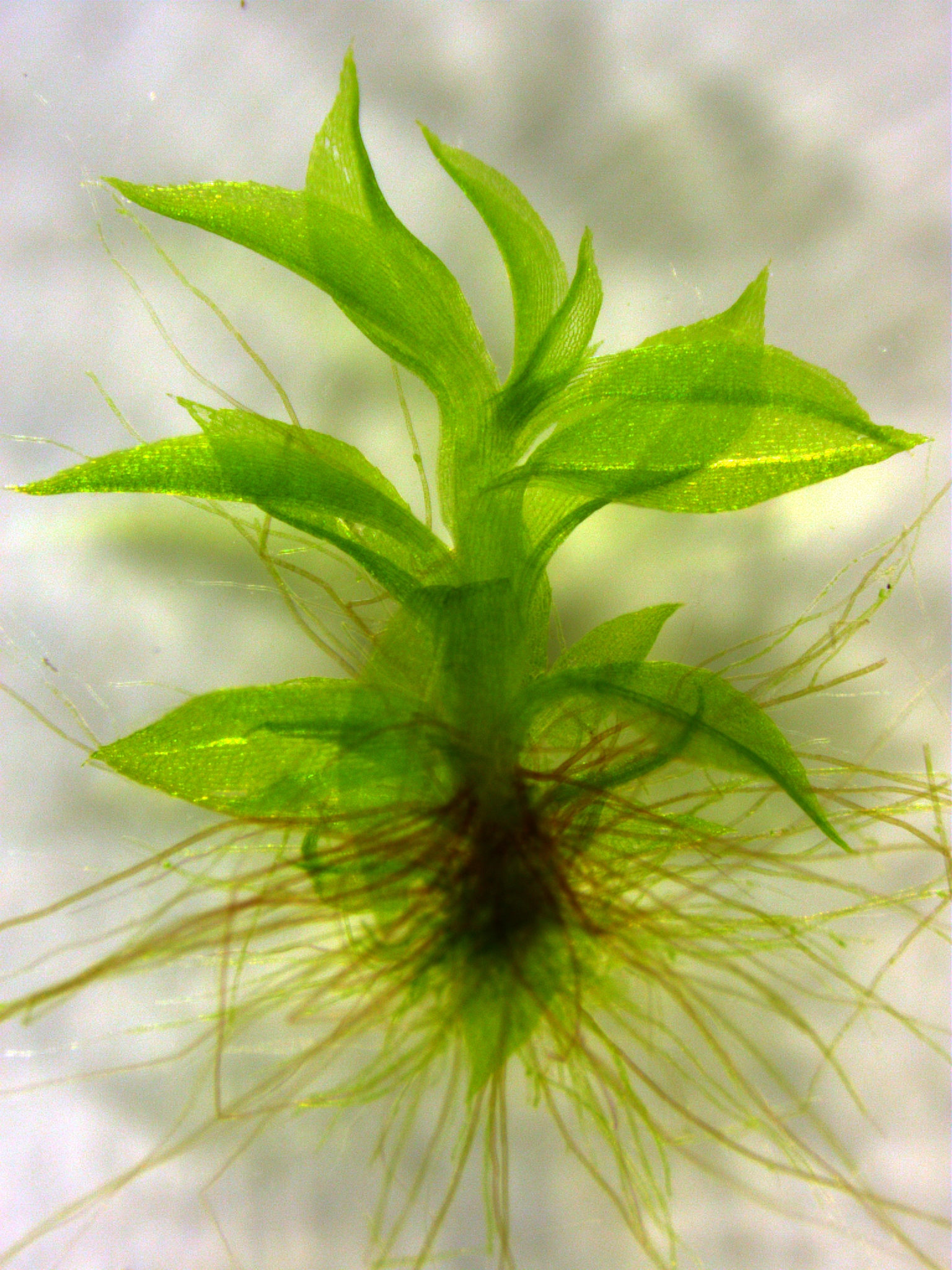

Supplement: Supplementary file 4 — Source Data [file 41467_2020_15967_MOESM4_ESM.zip › Raw data/Raw data for Figures/Figure 2c ppmacro2 #47.tif]

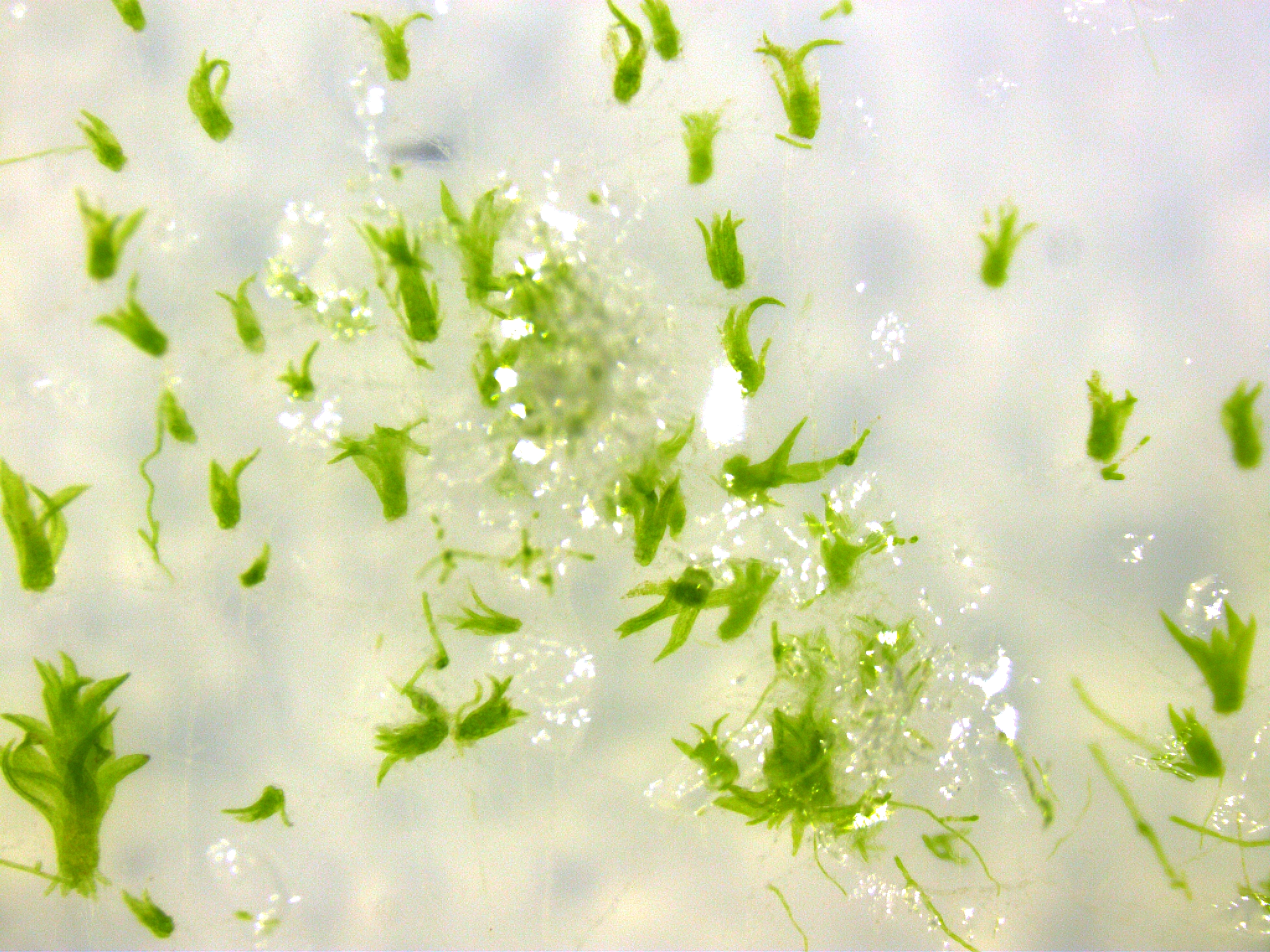

Supplement: Supplementary file 4 — Source Data [file 41467_2020_15967_MOESM4_ESM.zip › Raw data/Raw data for Figures/Figure 3a PpMACRO2-OE #48.tif]

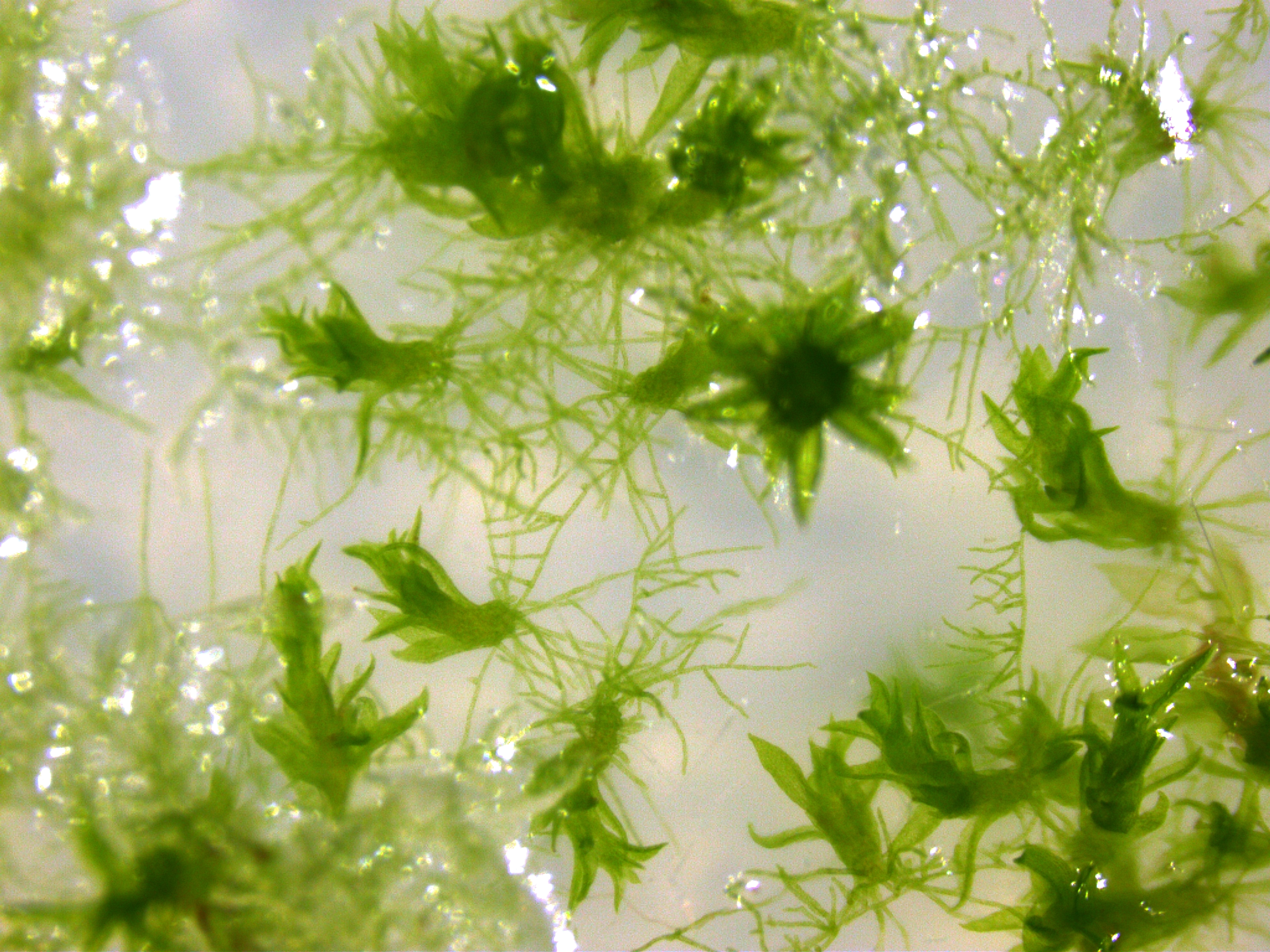

Supplement: Supplementary file 4 — Source Data [file 41467_2020_15967_MOESM4_ESM.zip › Raw data/Raw data for Figures/Figure 3a WT.tif]

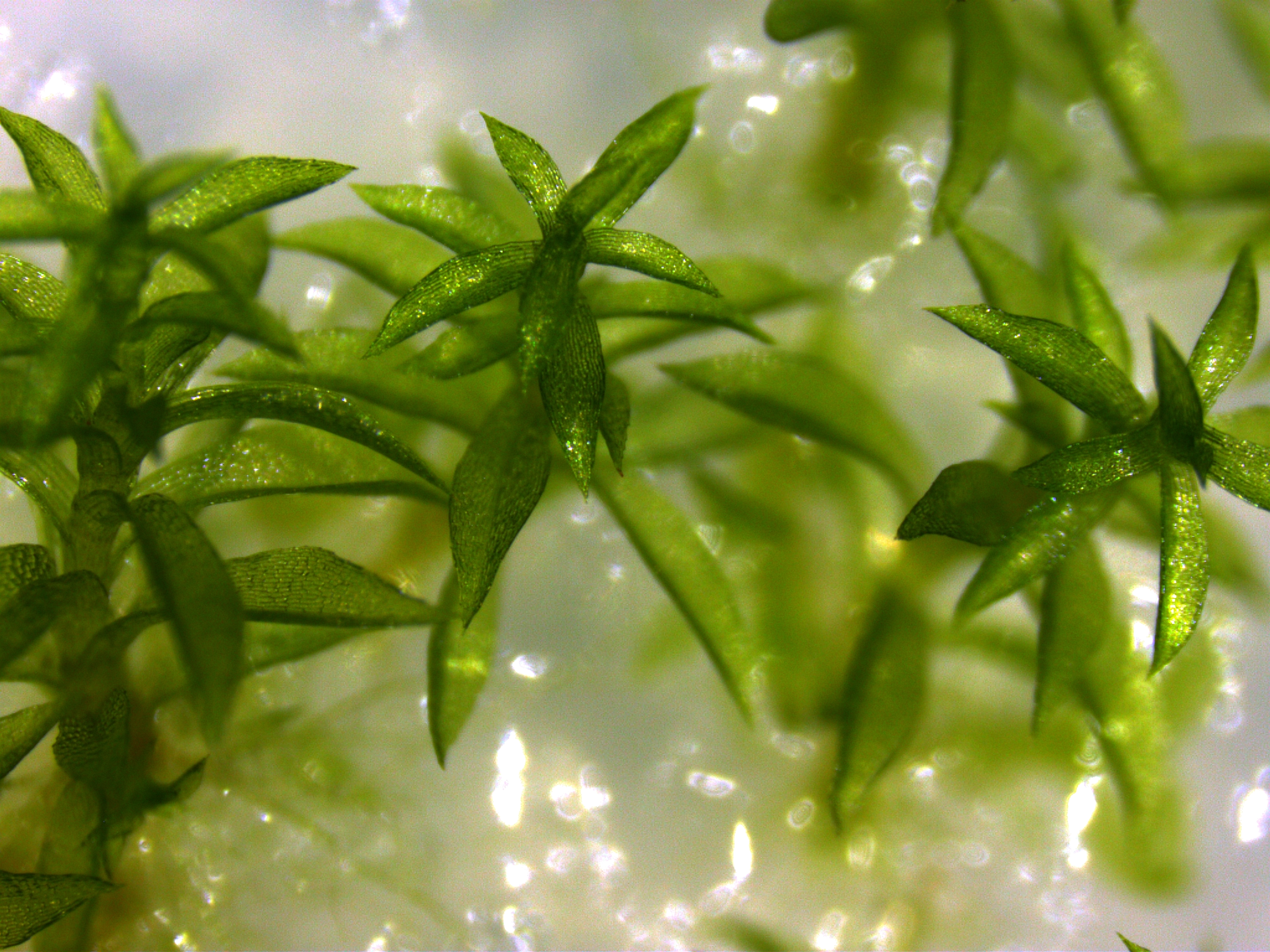

Supplement: Supplementary file 4 — Source Data [file 41467_2020_15967_MOESM4_ESM.zip › Raw data/Raw data for Figures/Figure 3a ppmacro2 #107.tif]

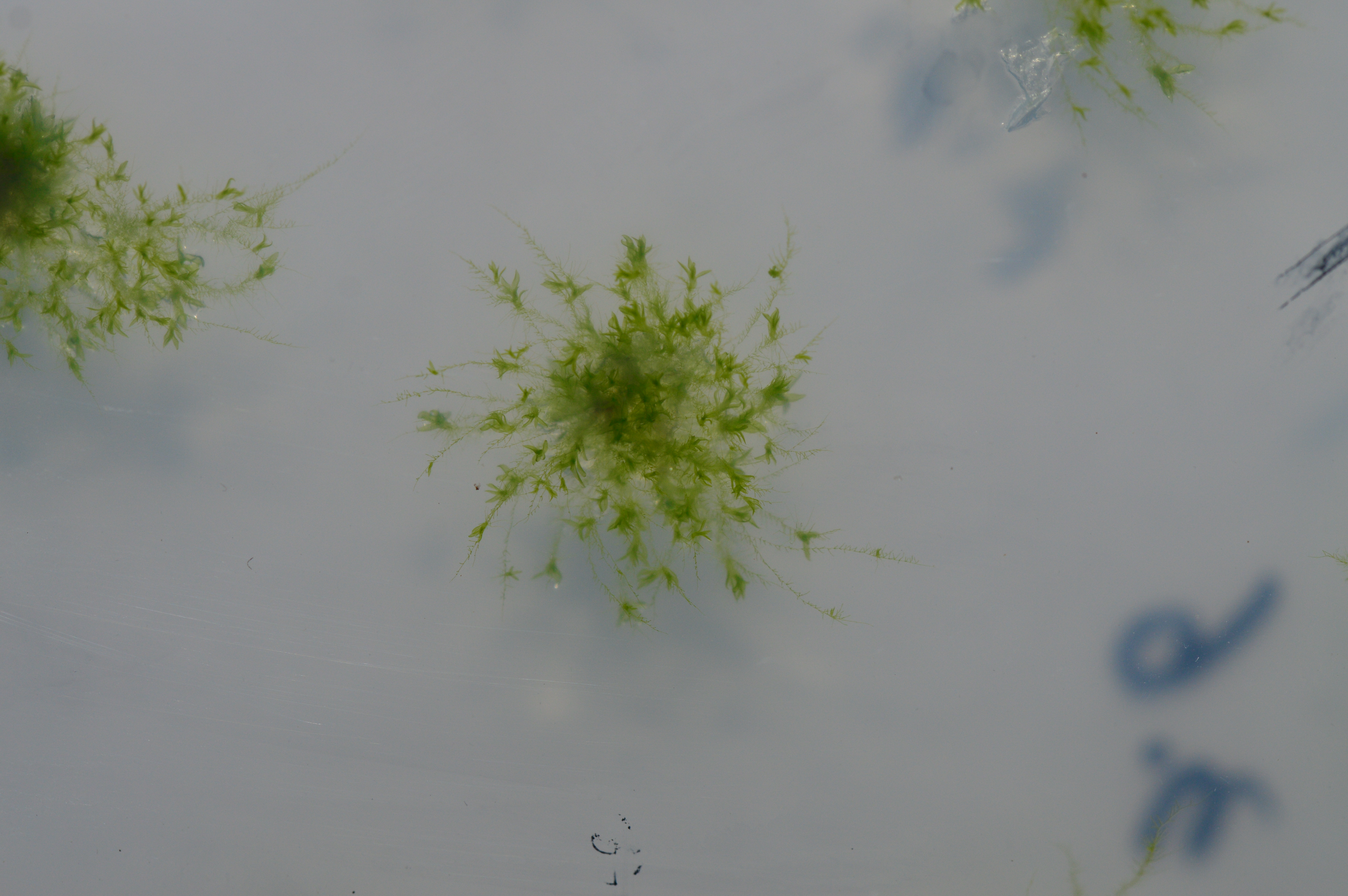

Supplement: Supplementary file 4 — Source Data [file 41467_2020_15967_MOESM4_ESM.zip › Raw data/Raw data for Figures/Figure 3b WT Clone.JPG]

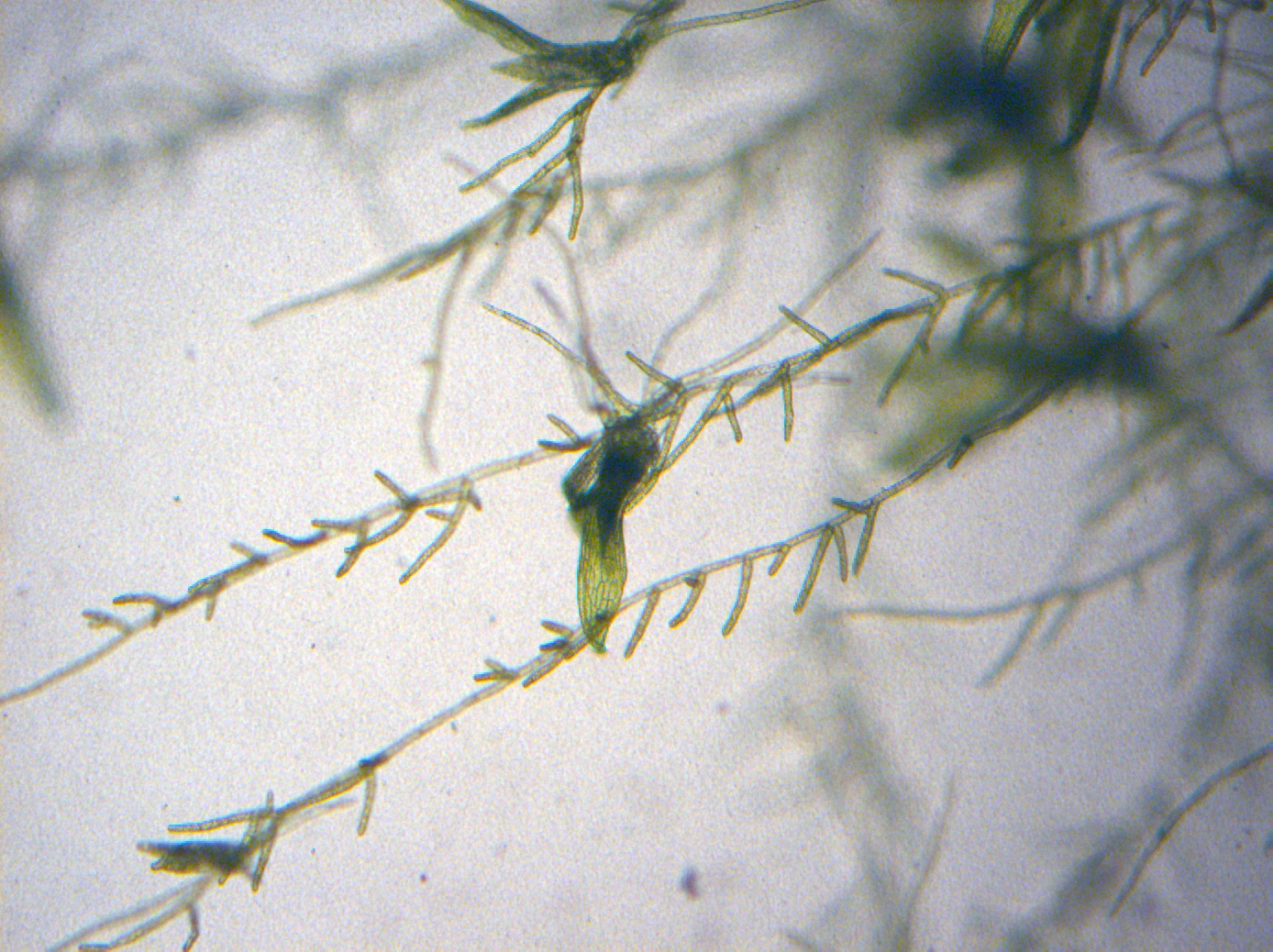

Supplement: Supplementary file 4 — Source Data [file 41467_2020_15967_MOESM4_ESM.zip › Raw data/Raw data for Figures/Figure 3b WT Filamenta.jpg]

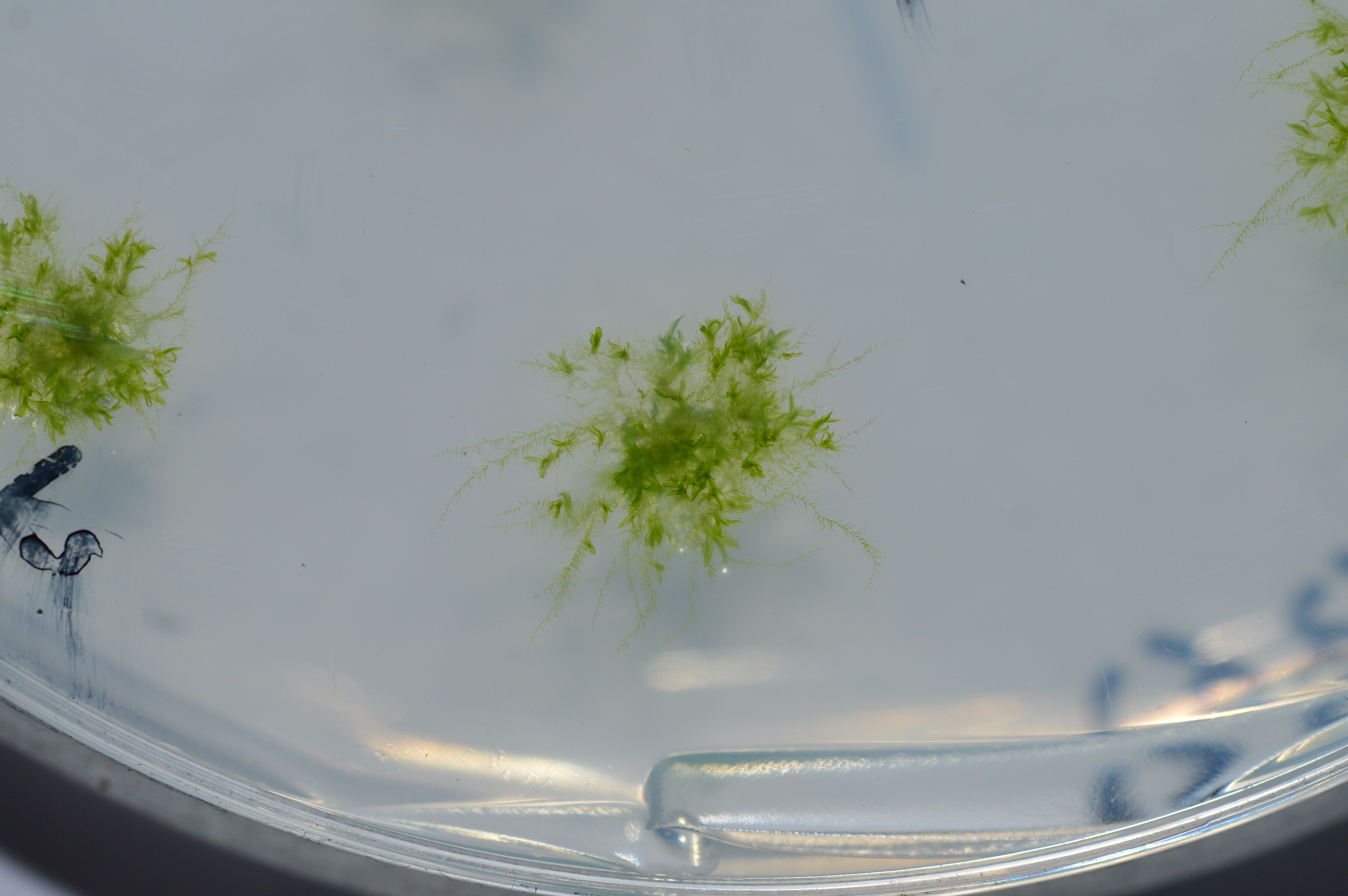

Supplement: Supplementary file 4 — Source Data [file 41467_2020_15967_MOESM4_ESM.zip › Raw data/Raw data for Figures/Figure 3b ppmacro2 #47 Clone.JPG]

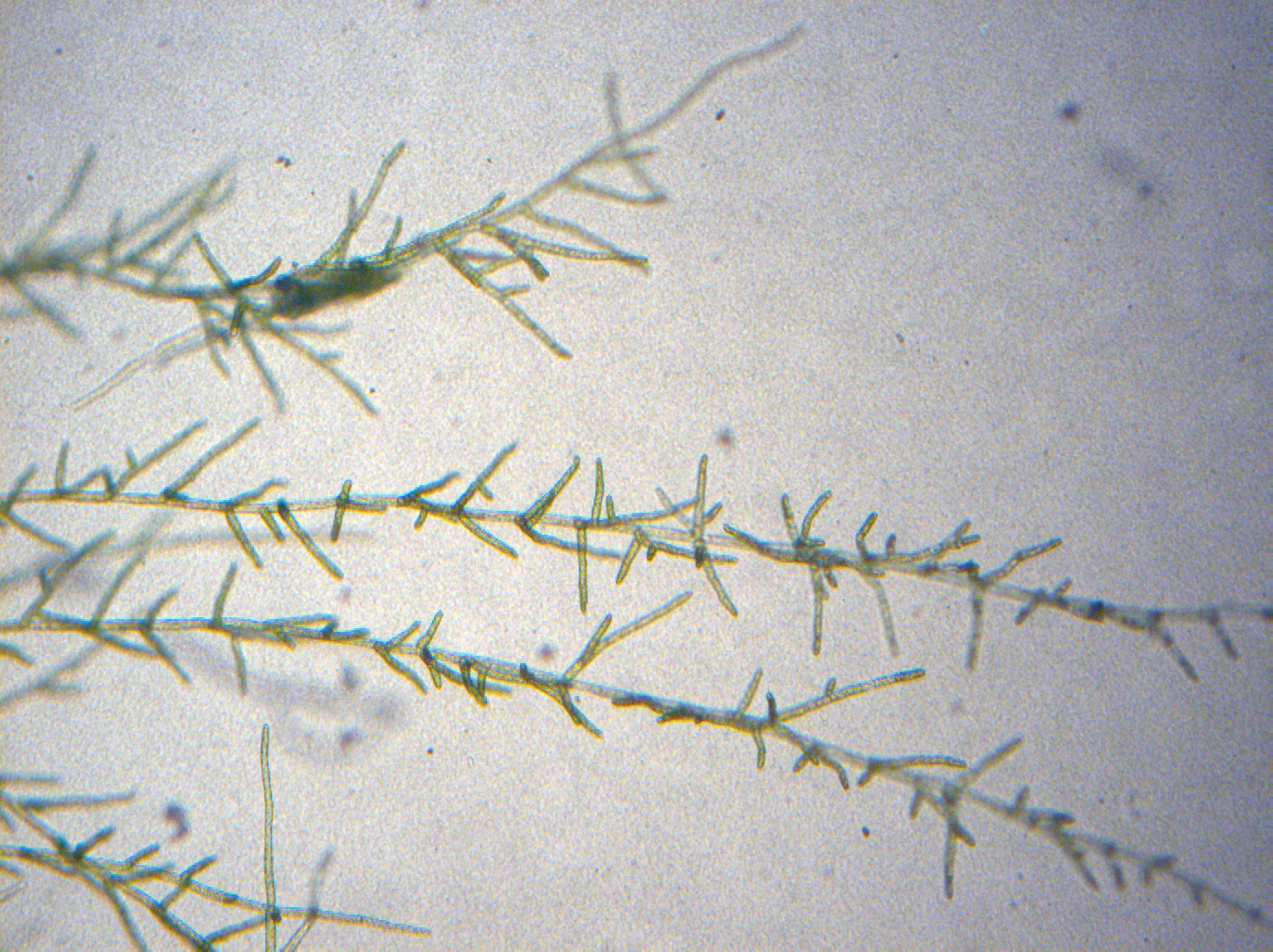

Supplement: Supplementary file 4 — Source Data [file 41467_2020_15967_MOESM4_ESM.zip › Raw data/Raw data for Figures/Figure 3b ppmacro2 #47 Filamenta.jpg]

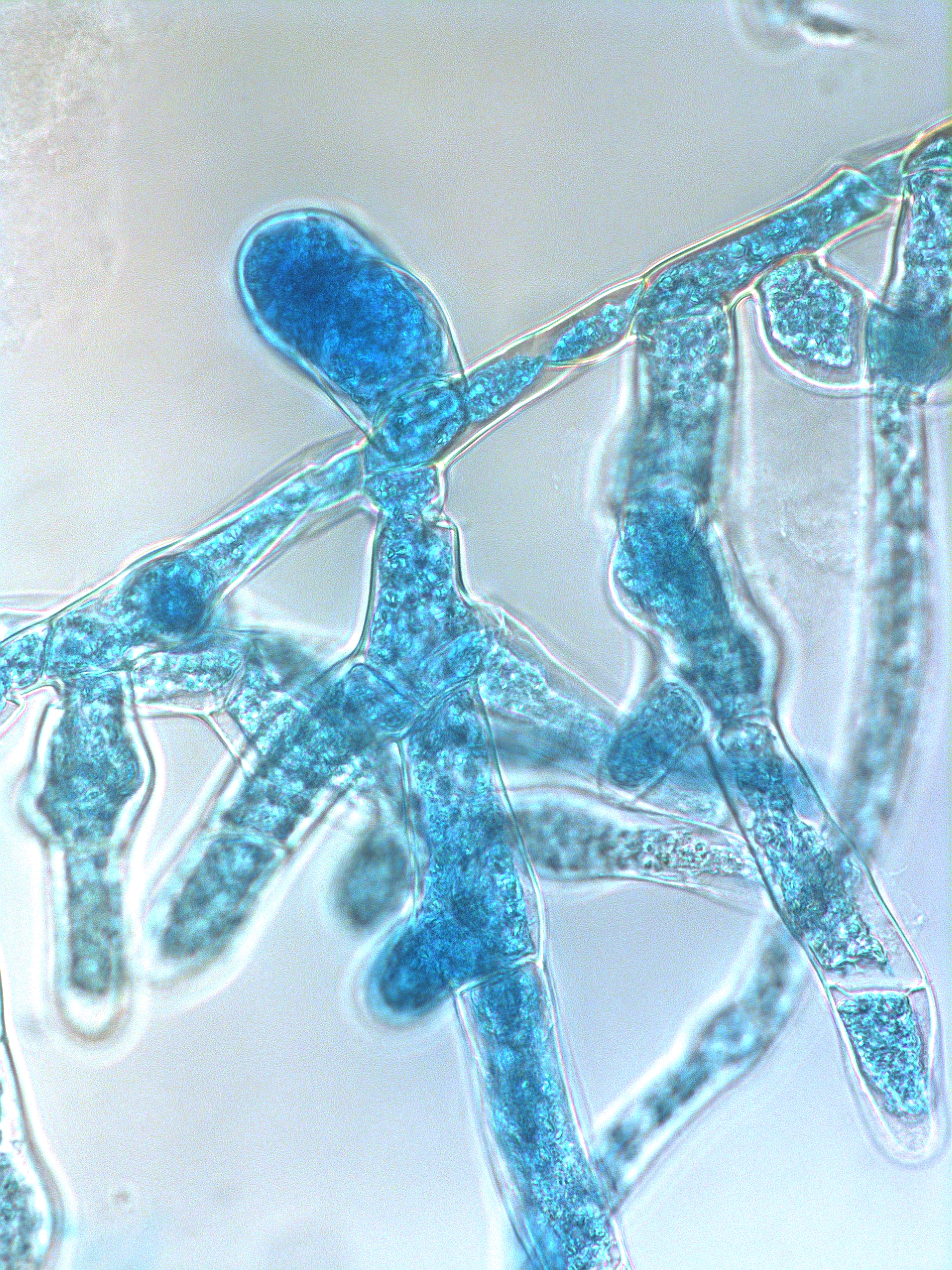

Supplement: Supplementary file 4 — Source Data [file 41467_2020_15967_MOESM4_ESM.zip › Raw data/Raw data for Figures/Figure 3e -1.tif]

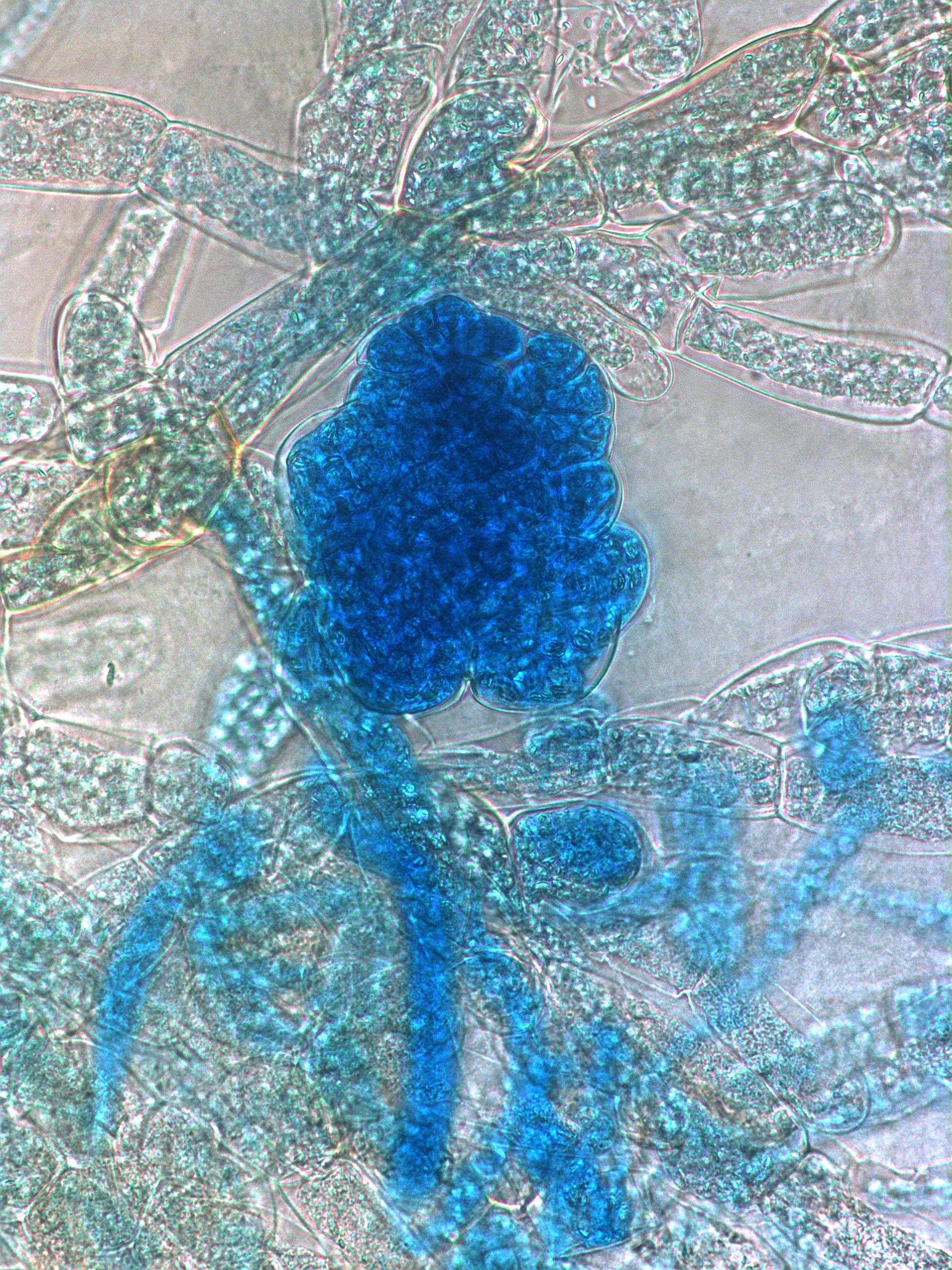

Supplement: Supplementary file 4 — Source Data [file 41467_2020_15967_MOESM4_ESM.zip › Raw data/Raw data for Figures/Figure 3e -2.tif]

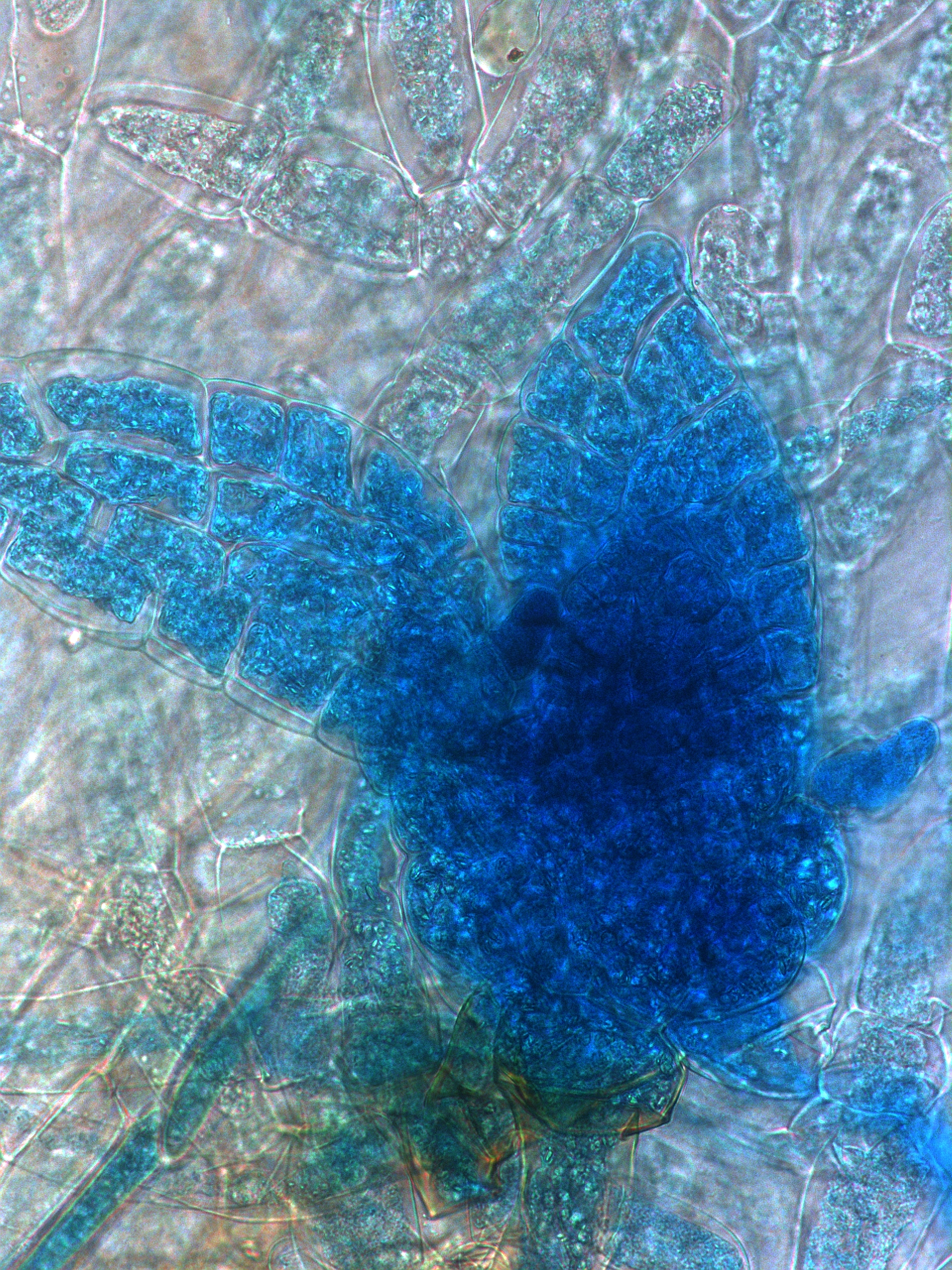

Supplement: Supplementary file 4 — Source Data [file 41467_2020_15967_MOESM4_ESM.zip › Raw data/Raw data for Figures/Figure 3e -3.tif]

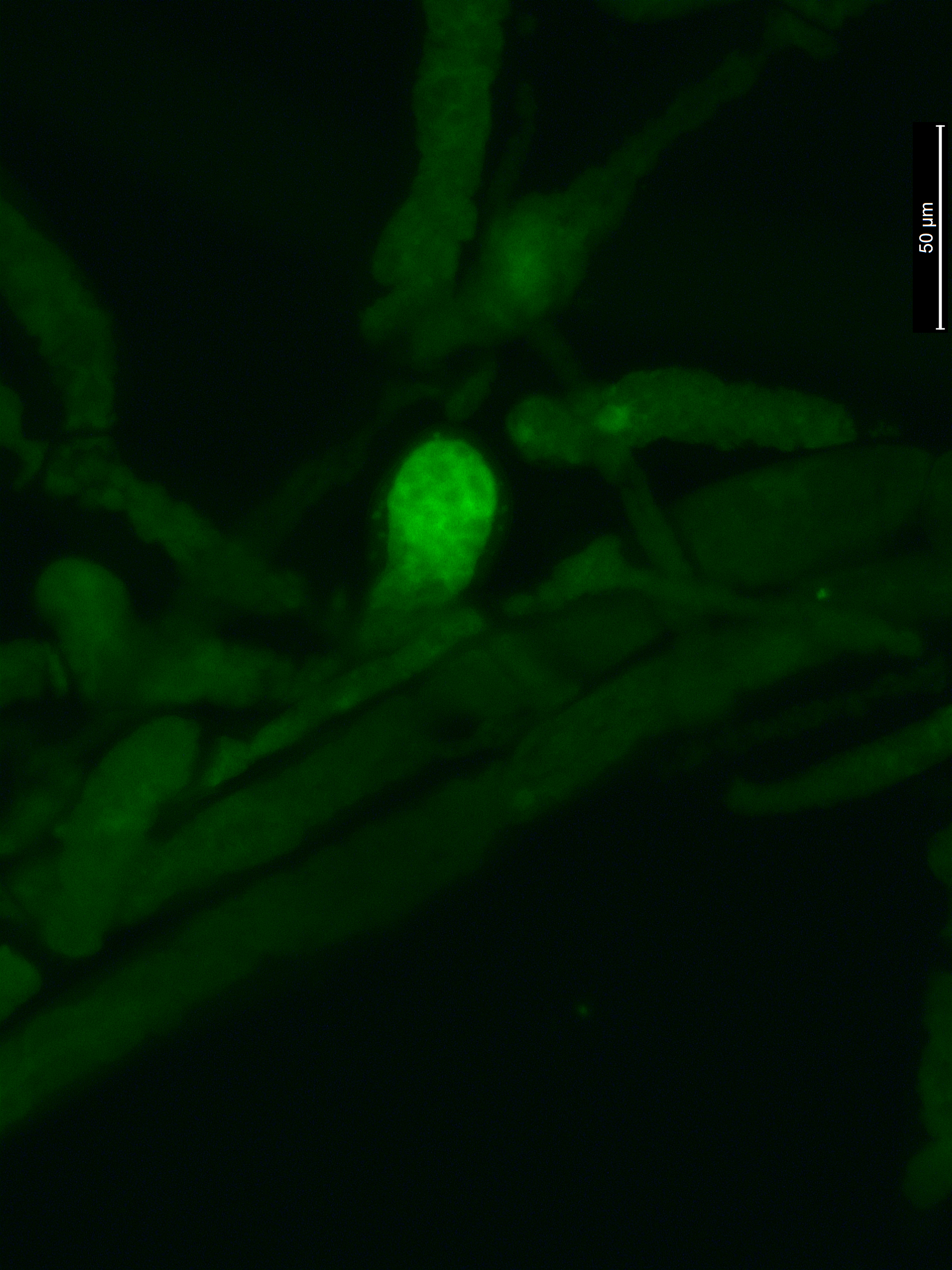

Supplement: Supplementary file 4 — Source Data [file 41467_2020_15967_MOESM4_ESM.zip › Raw data/Raw data for Figures/Figure 3f -1.tif]

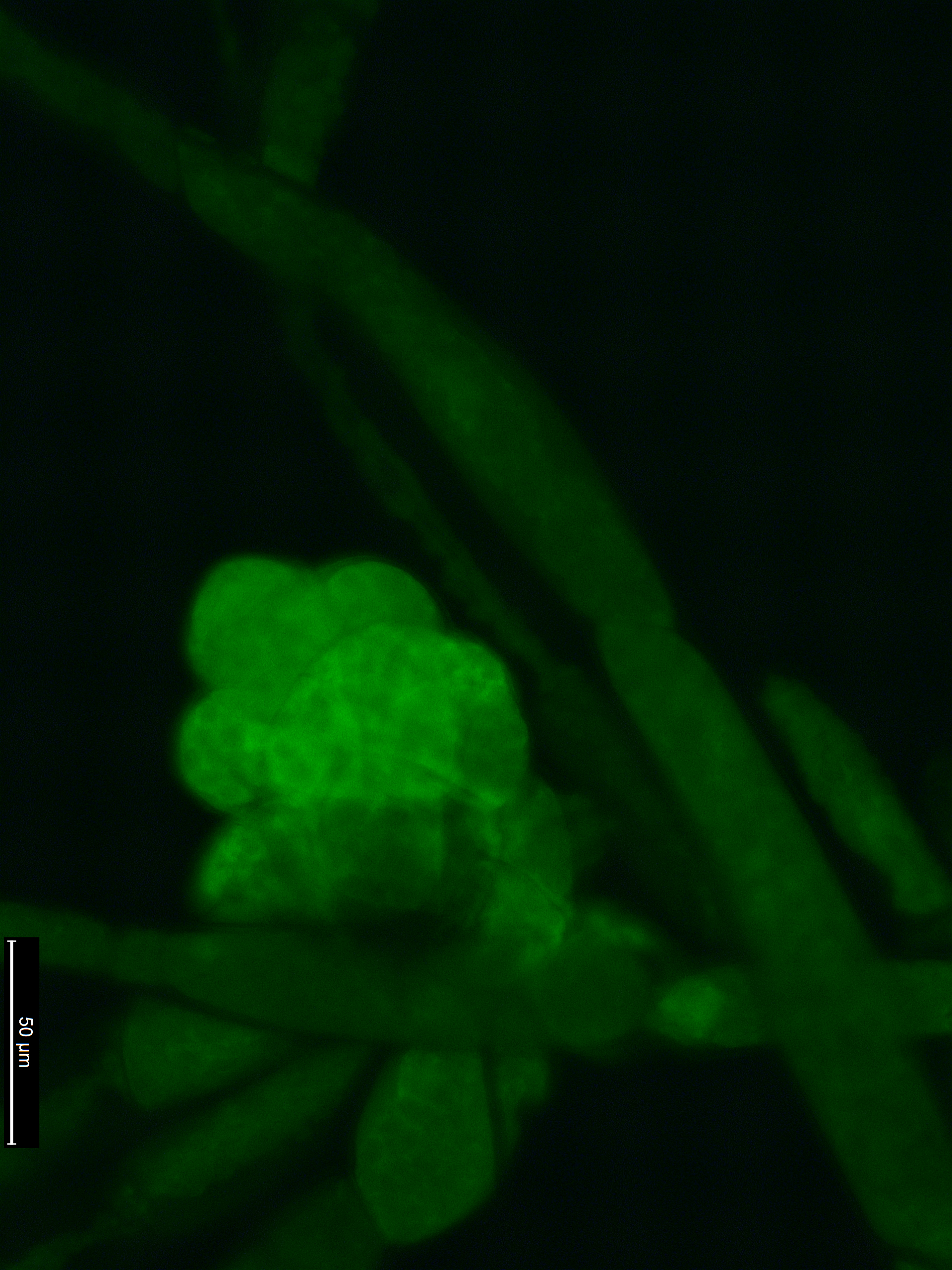

Supplement: Supplementary file 4 — Source Data [file 41467_2020_15967_MOESM4_ESM.zip › Raw data/Raw data for Figures/Figure 3f -2.tif]

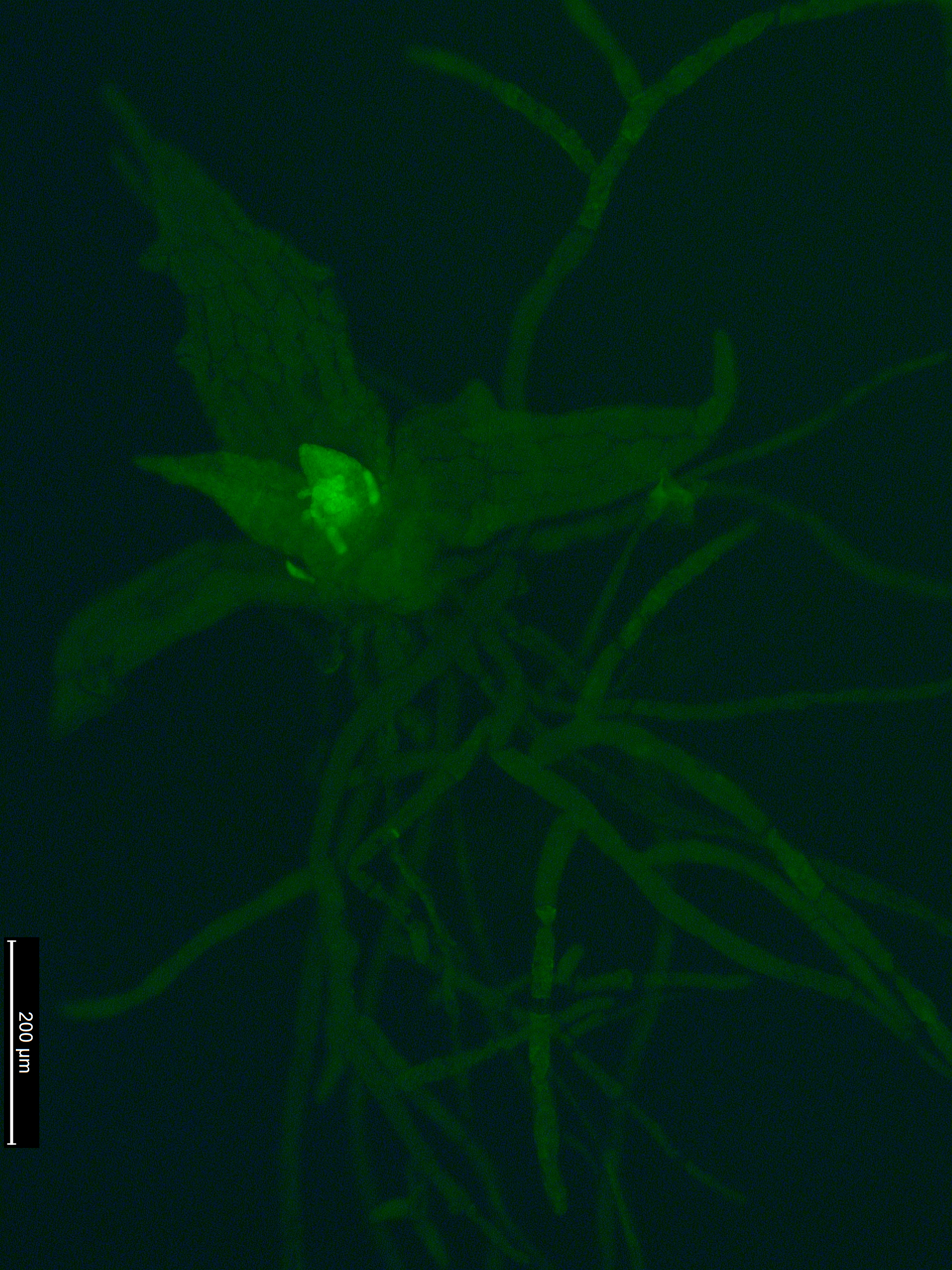

Supplement: Supplementary file 4 — Source Data [file 41467_2020_15967_MOESM4_ESM.zip › Raw data/Raw data for Figures/Figure 3f -3.tif]

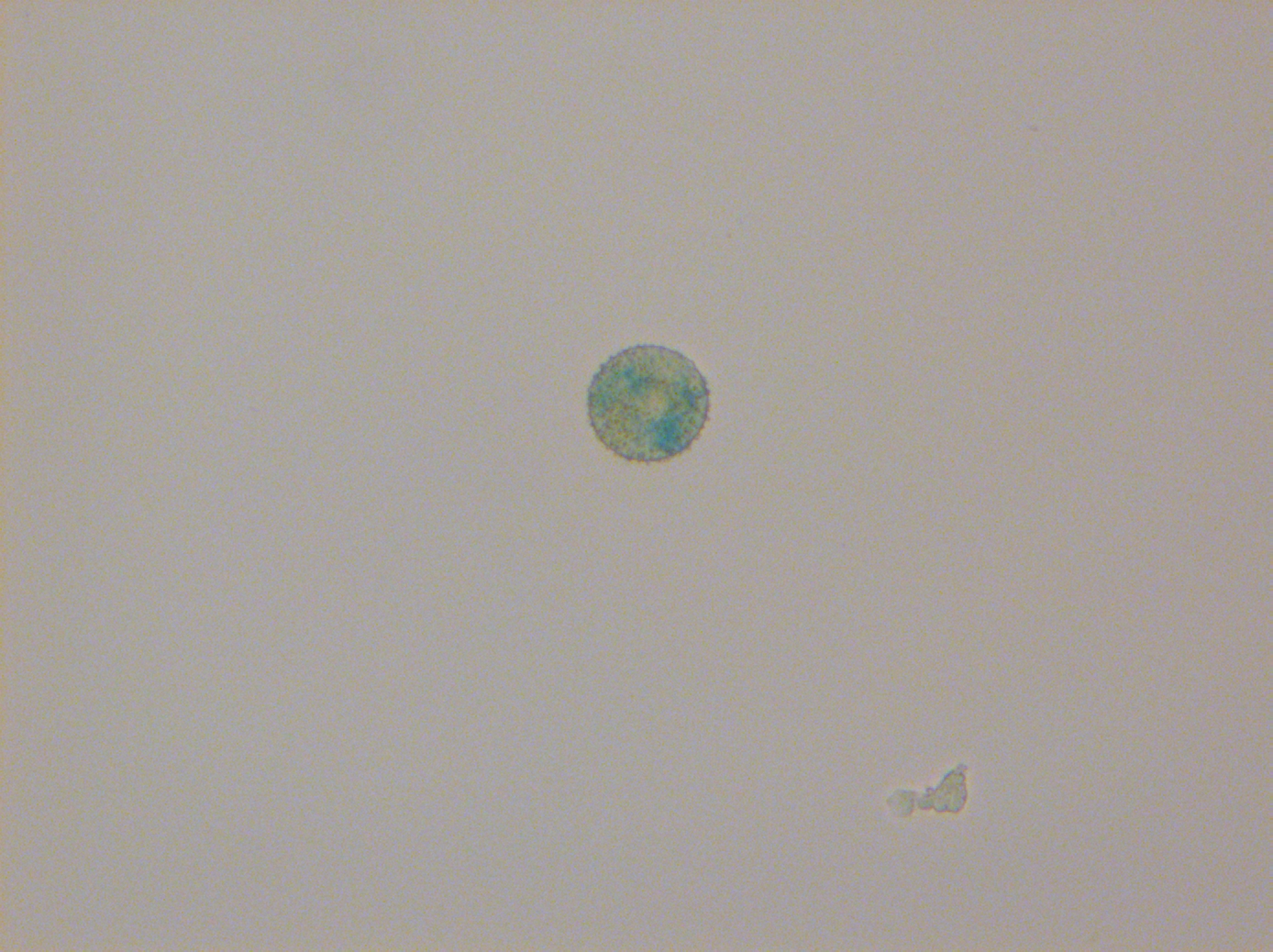

Supplement: Supplementary file 4 — Source Data [file 41467_2020_15967_MOESM4_ESM.zip › Raw data/Raw data for Figures/Figure 4a.jpg]

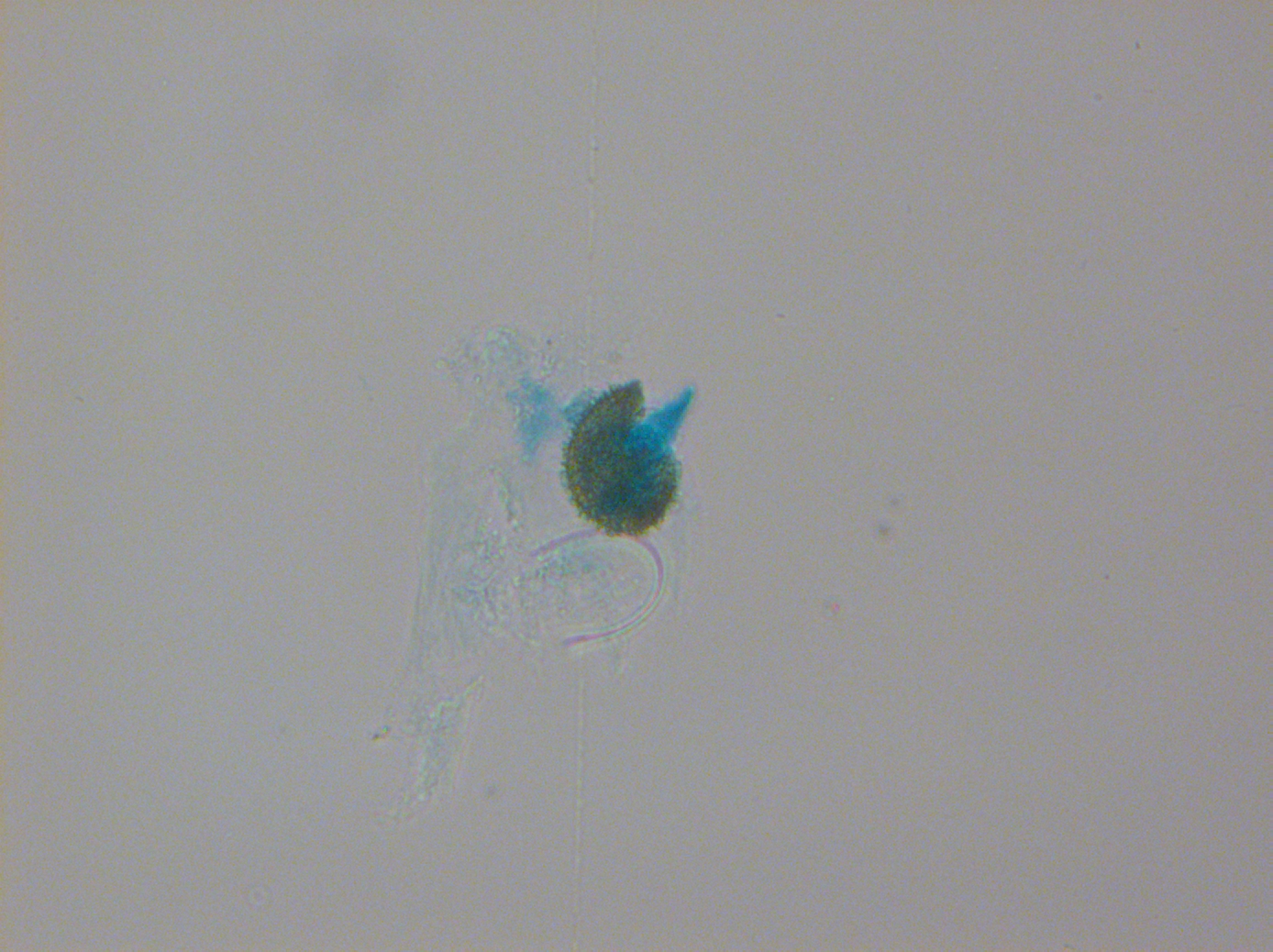

Supplement: Supplementary file 4 — Source Data [file 41467_2020_15967_MOESM4_ESM.zip › Raw data/Raw data for Figures/Figure 4b.jpg]

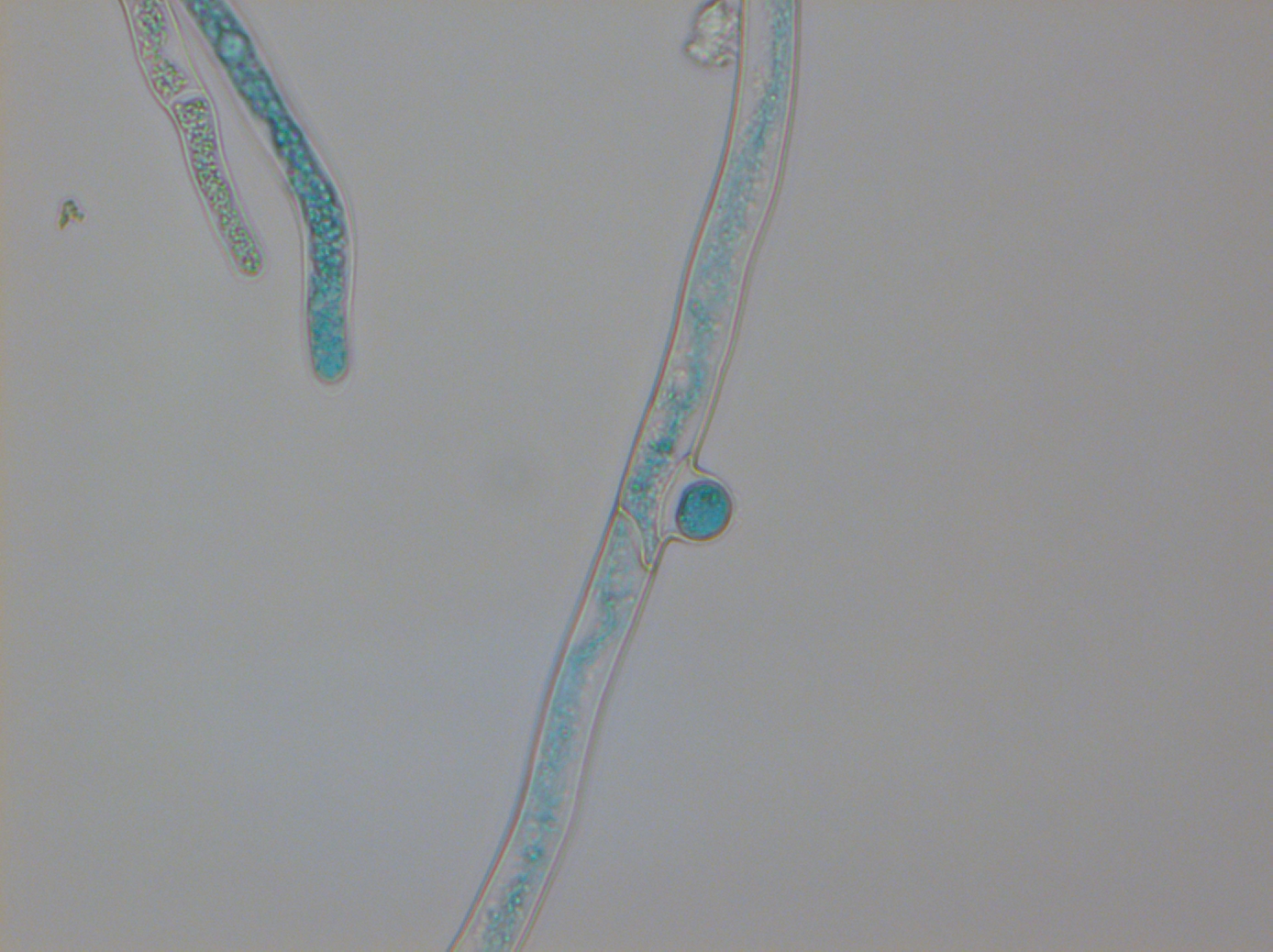

Supplement: Supplementary file 4 — Source Data [file 41467_2020_15967_MOESM4_ESM.zip › Raw data/Raw data for Figures/Figure 4c.jpg]

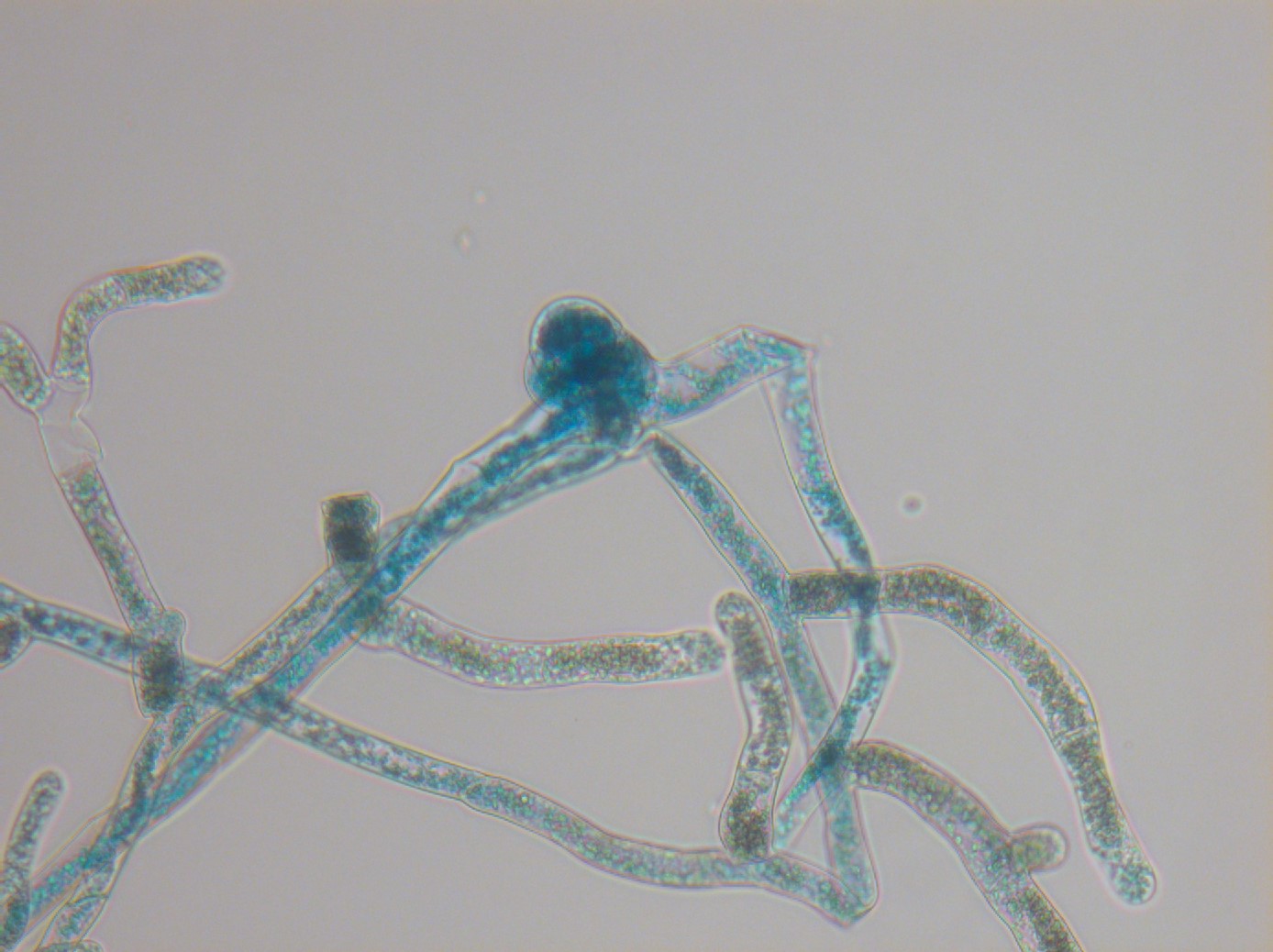

Supplement: Supplementary file 4 — Source Data [file 41467_2020_15967_MOESM4_ESM.zip › Raw data/Raw data for Figures/Figure 4d.jpg]

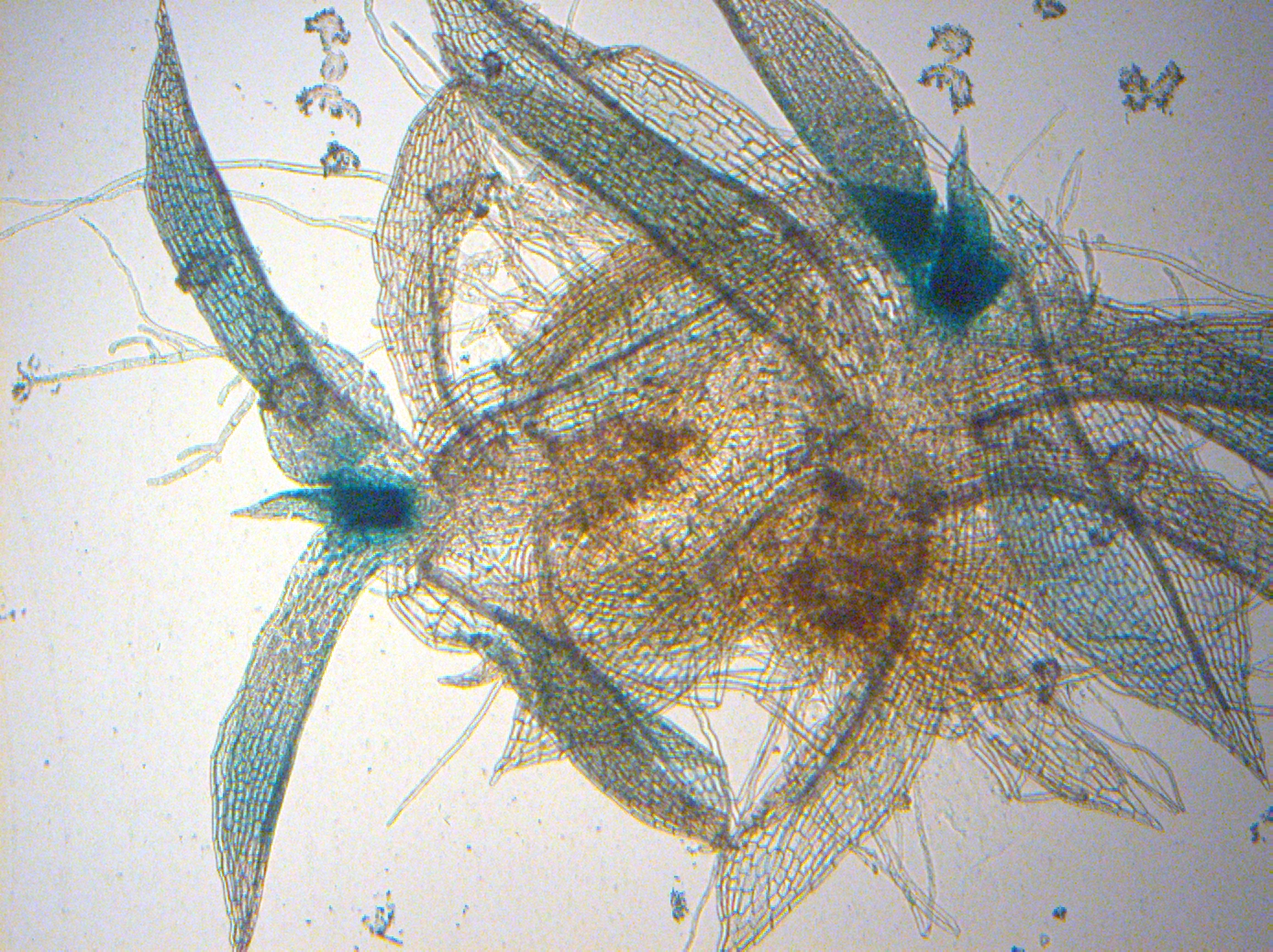

Supplement: Supplementary file 4 — Source Data [file 41467_2020_15967_MOESM4_ESM.zip › Raw data/Raw data for Figures/Figure 4e.jpg]

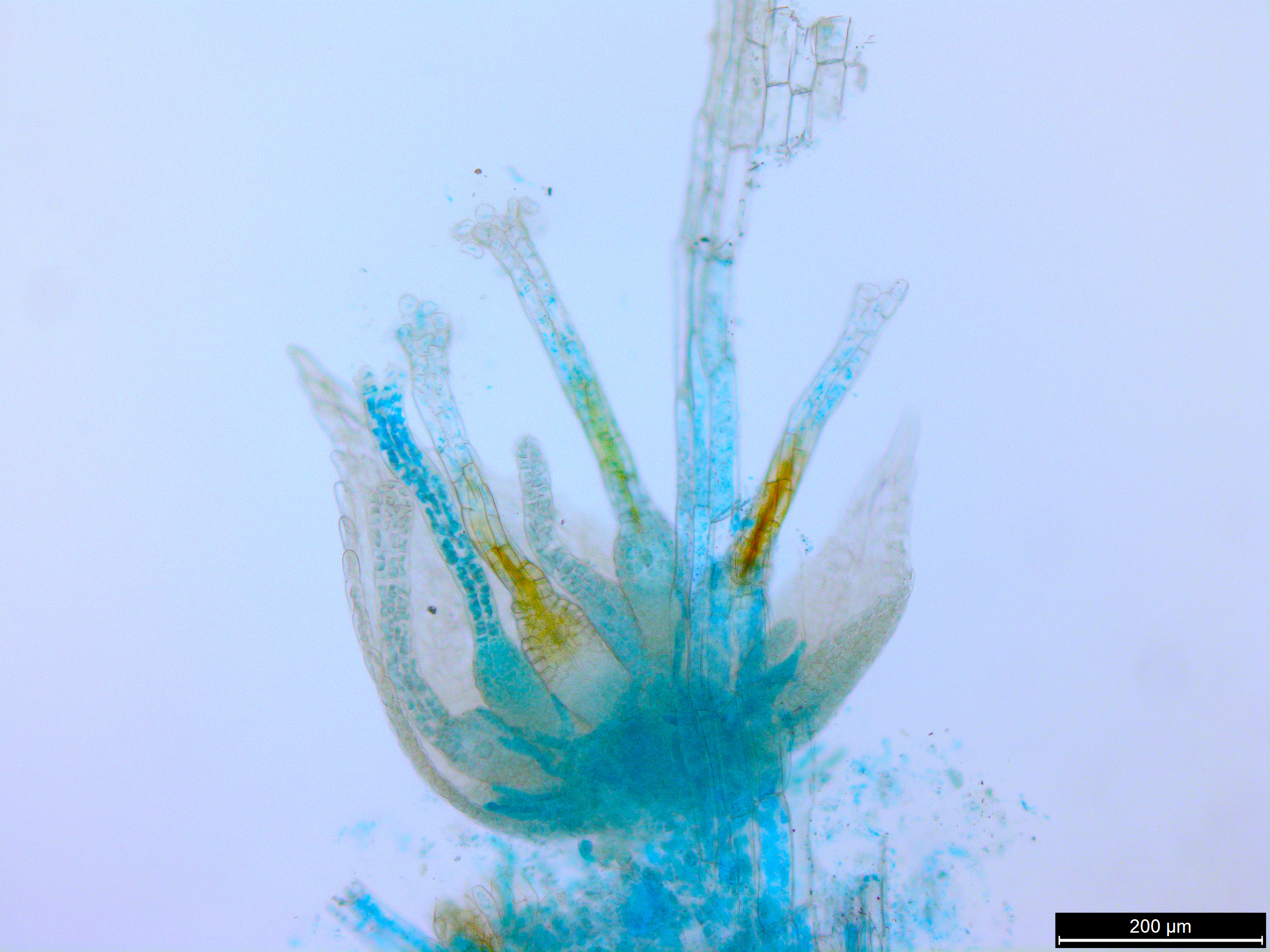

Supplement: Supplementary file 4 — Source Data [file 41467_2020_15967_MOESM4_ESM.zip › Raw data/Raw data for Figures/Figure 4f.tif]

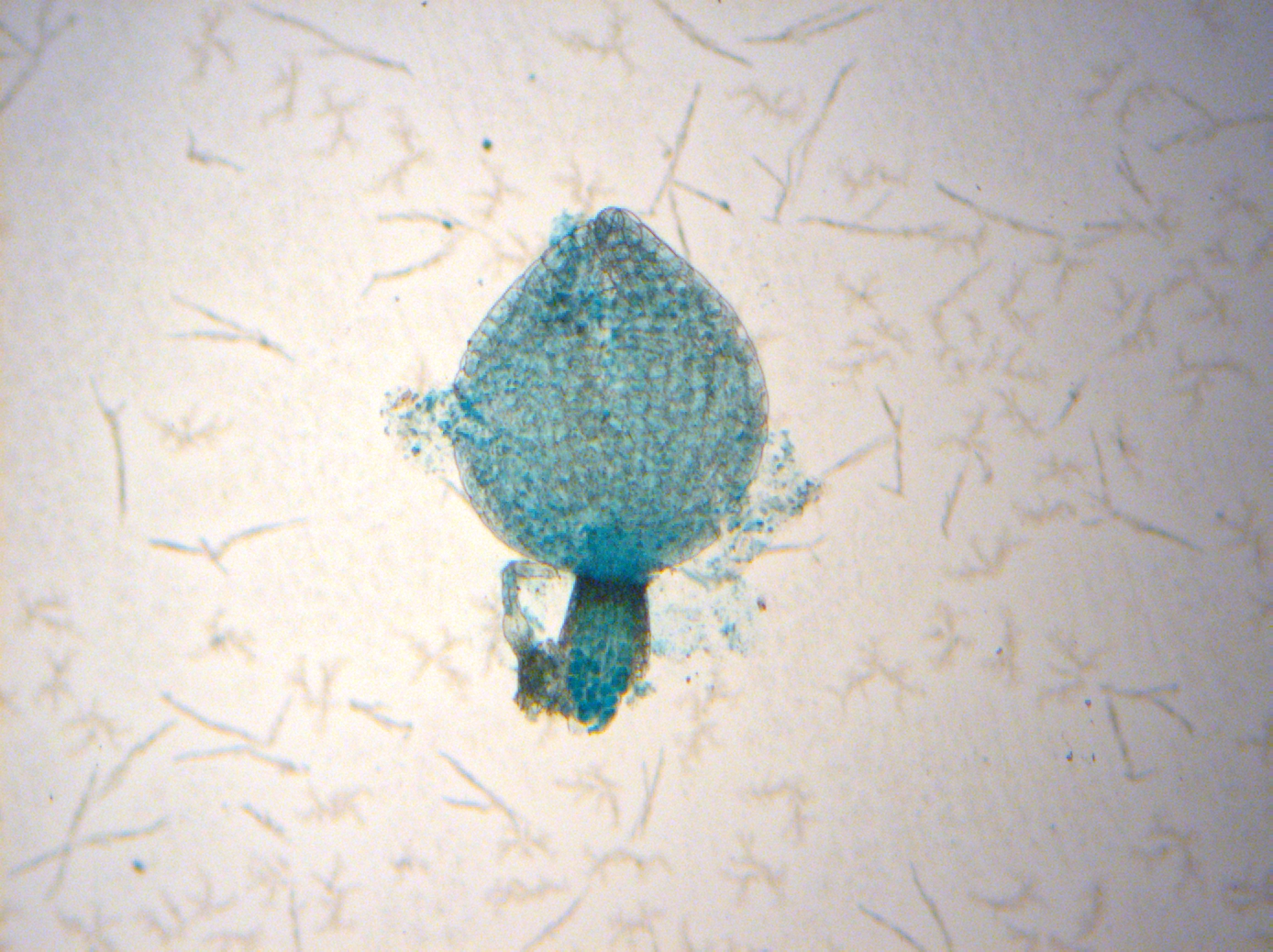

Supplement: Supplementary file 4 — Source Data [file 41467_2020_15967_MOESM4_ESM.zip › Raw data/Raw data for Figures/Figure 4g.jpg]

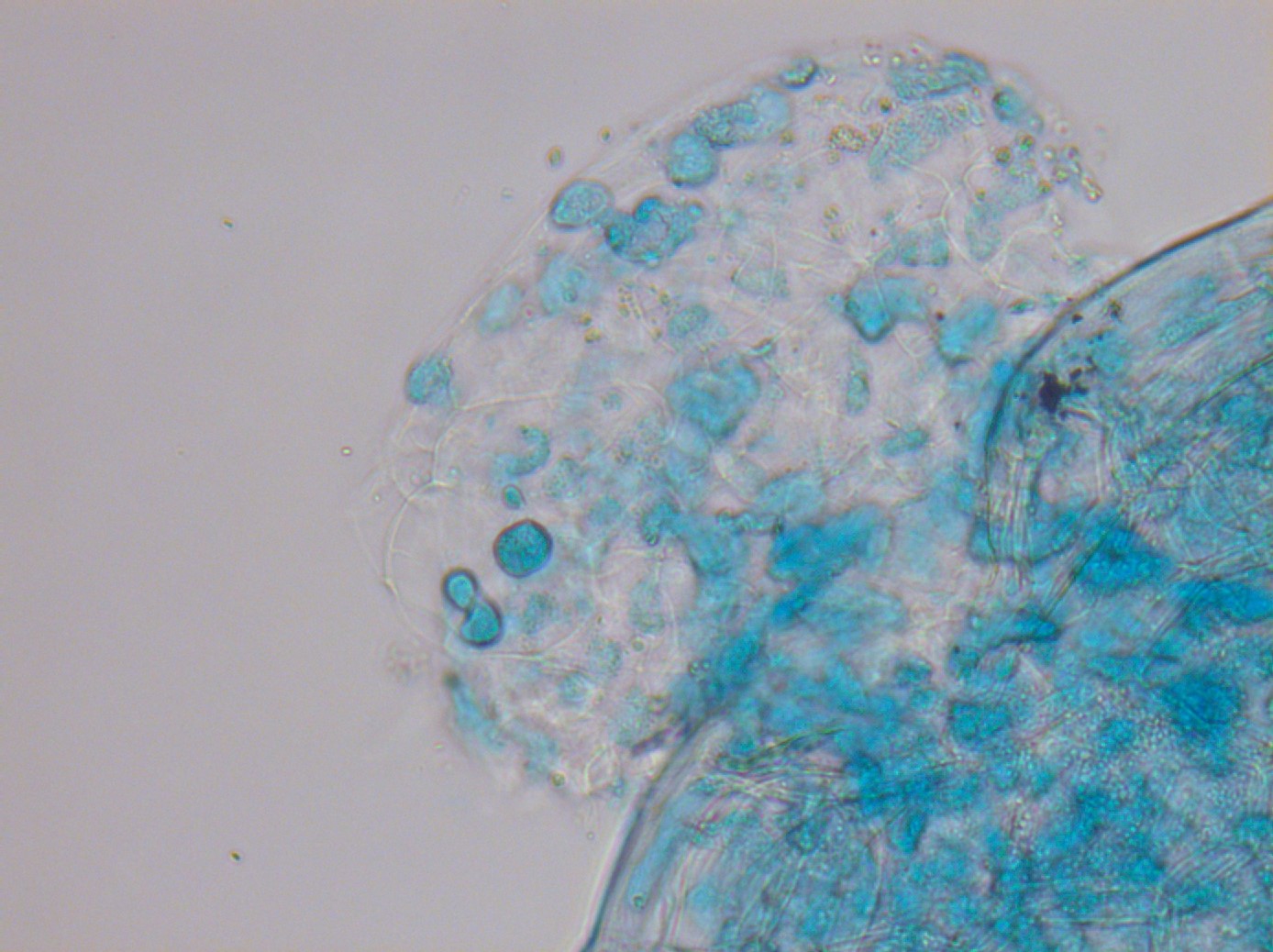

Supplement: Supplementary file 4 — Source Data [file 41467_2020_15967_MOESM4_ESM.zip › Raw data/Raw data for Figures/Figure 4h.jpg]

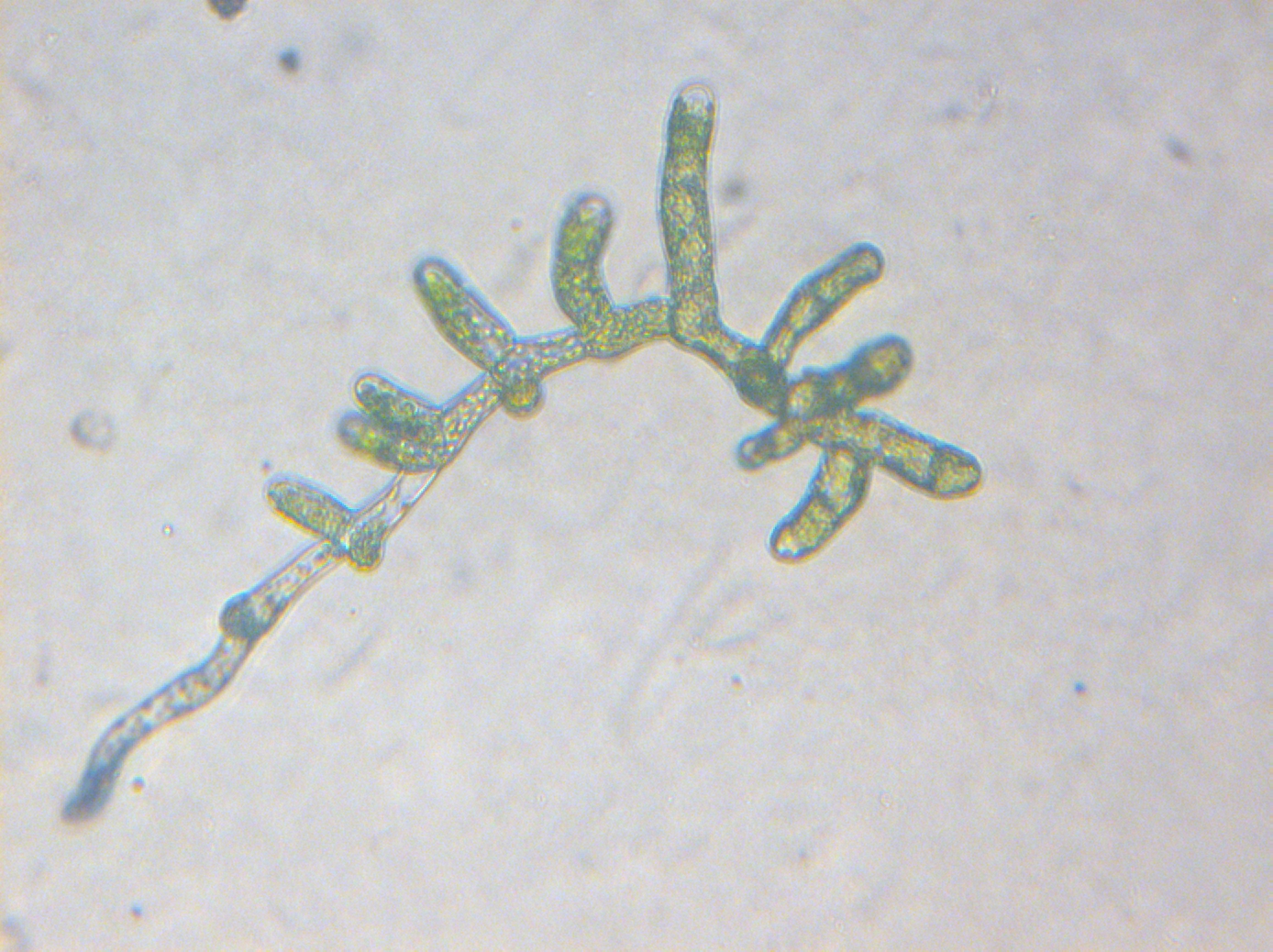

Supplement: Supplementary file 4 — Source Data [file 41467_2020_15967_MOESM4_ESM.zip › Raw data/Raw data for Figures/Figure 5a PpMACRO2-OE #48 10d.jpg]

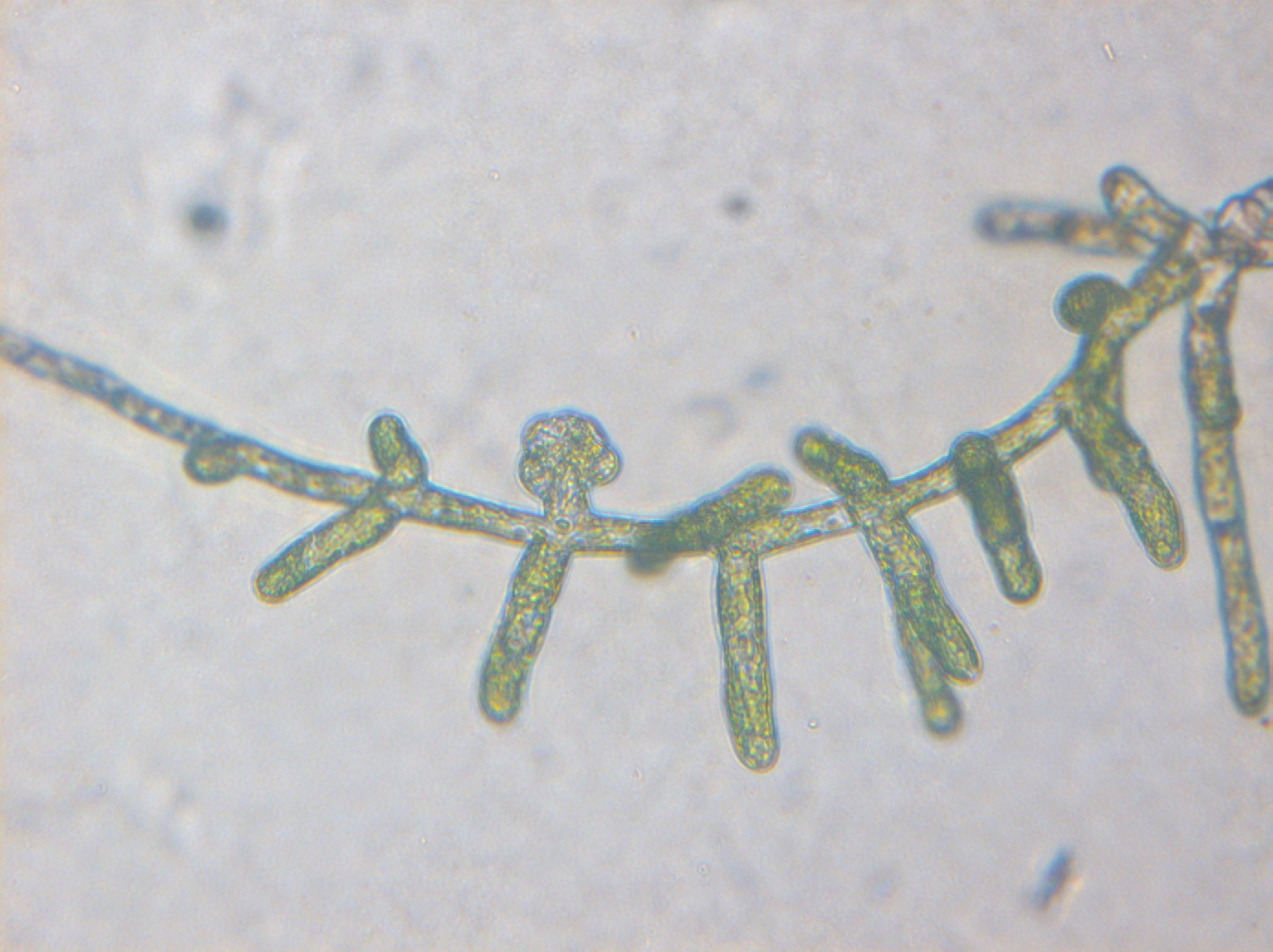

Supplement: Supplementary file 4 — Source Data [file 41467_2020_15967_MOESM4_ESM.zip › Raw data/Raw data for Figures/Figure 5a PpMACRO2-OE #48 13d.jpg]

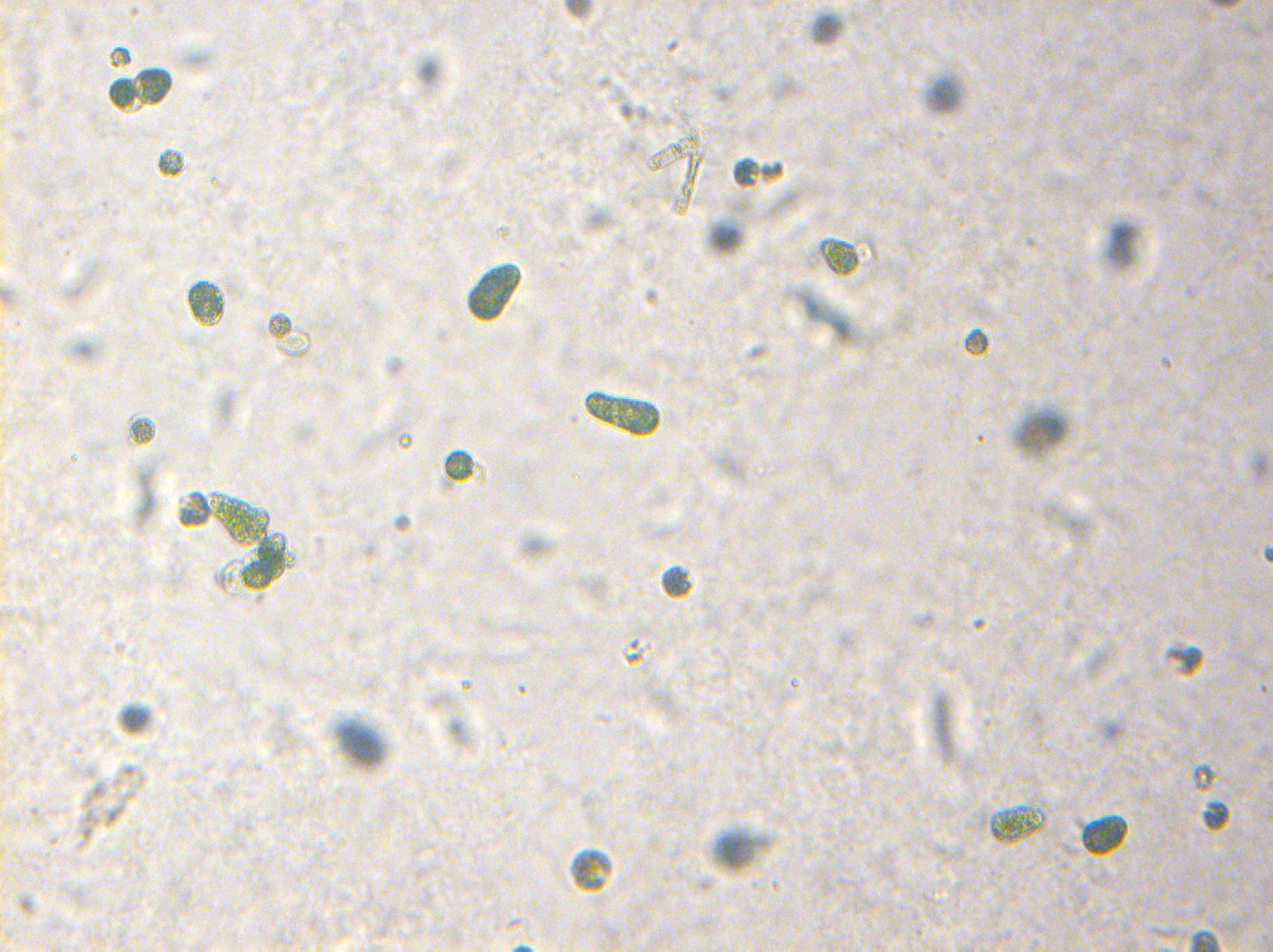

Supplement: Supplementary file 4 — Source Data [file 41467_2020_15967_MOESM4_ESM.zip › Raw data/Raw data for Figures/Figure 5a PpMACRO2-OE #48 2d.jpg]

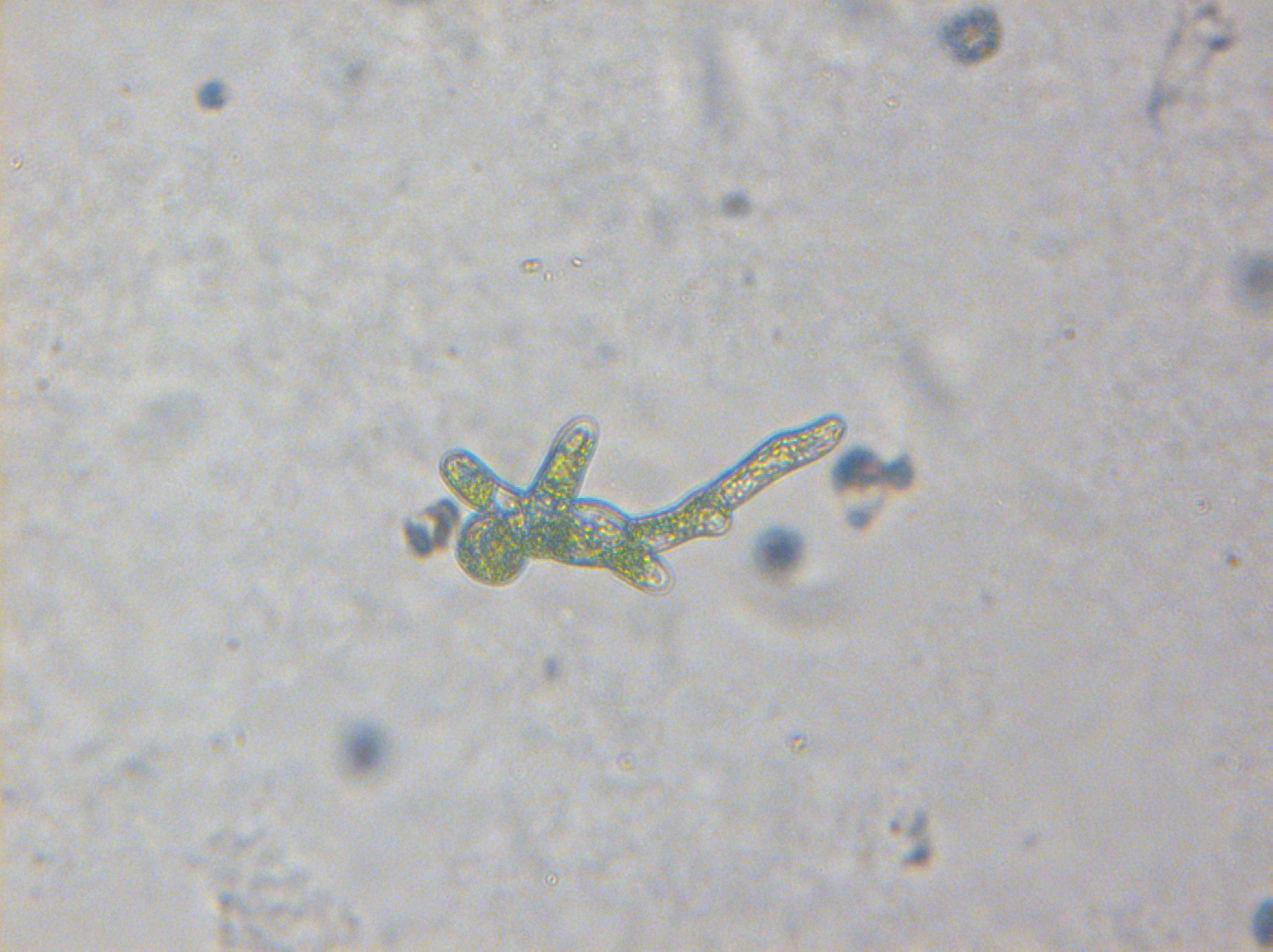

Supplement: Supplementary file 4 — Source Data [file 41467_2020_15967_MOESM4_ESM.zip › Raw data/Raw data for Figures/Figure 5a PpMACRO2-OE #48 5d.jpg]

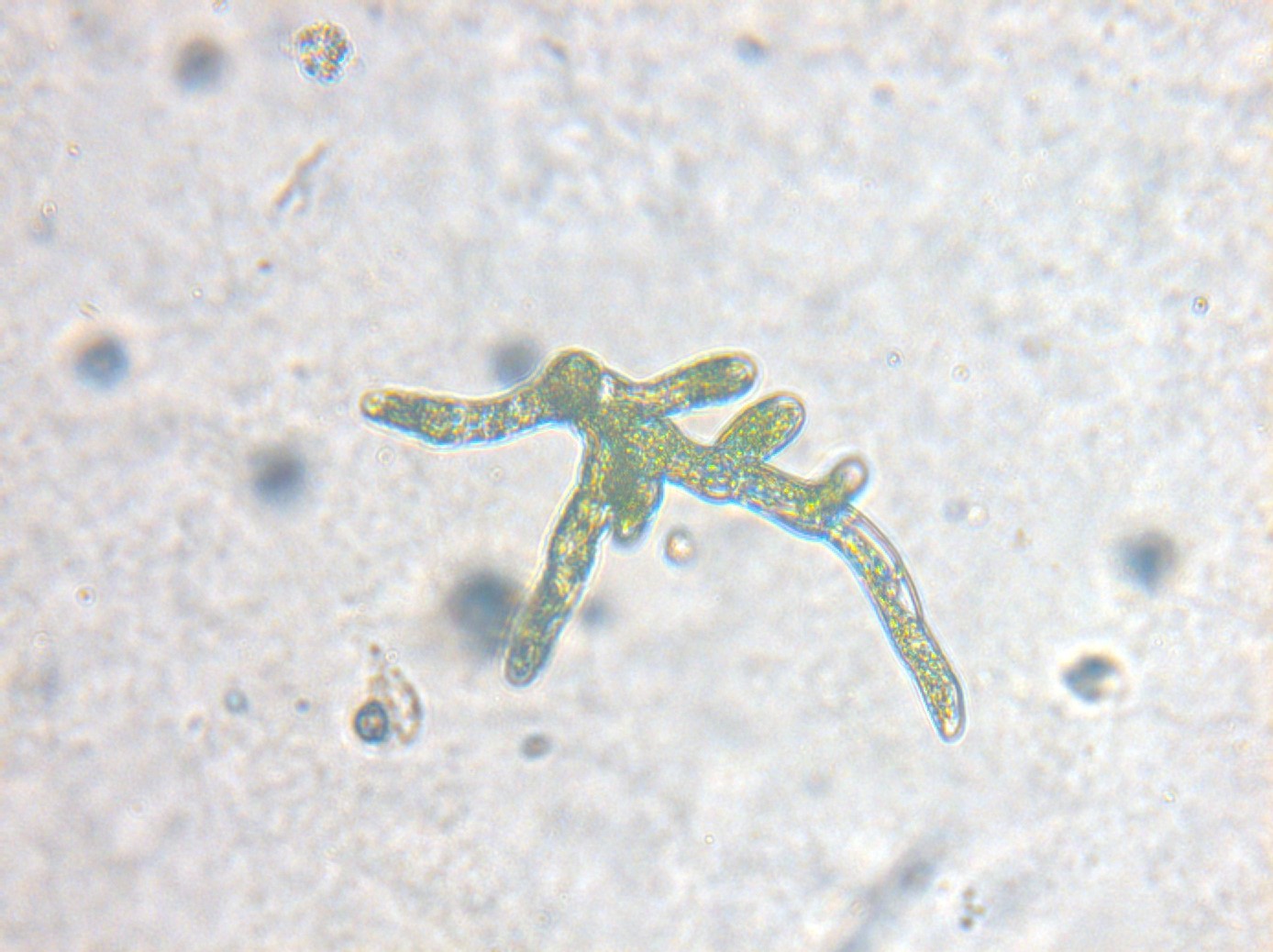

Supplement: Supplementary file 4 — Source Data [file 41467_2020_15967_MOESM4_ESM.zip › Raw data/Raw data for Figures/Figure 5a PpMACRO2-OE #48 7d.jpg]

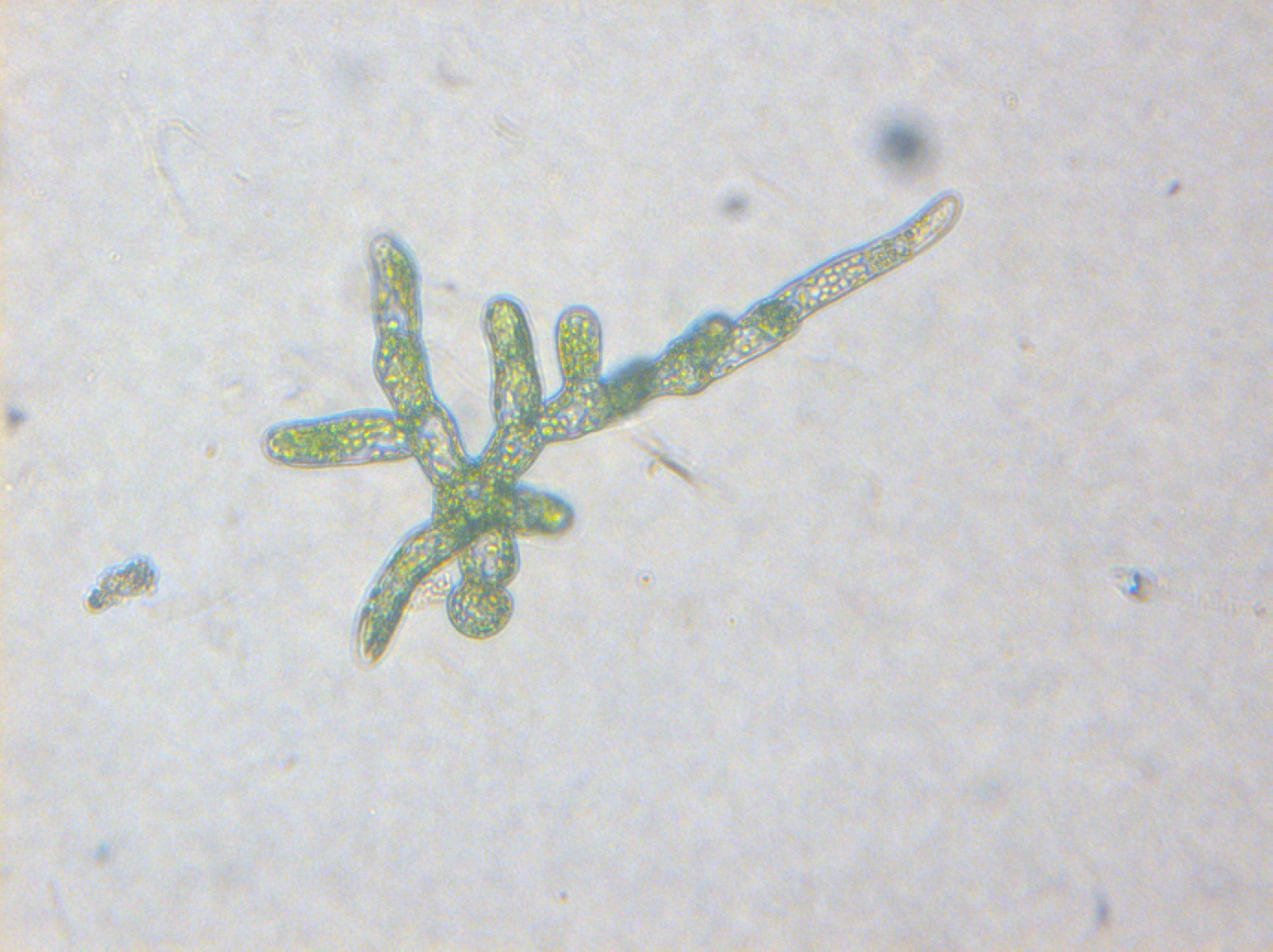

Supplement: Supplementary file 4 — Source Data [file 41467_2020_15967_MOESM4_ESM.zip › Raw data/Raw data for Figures/Figure 5a WT 10d.jpg]

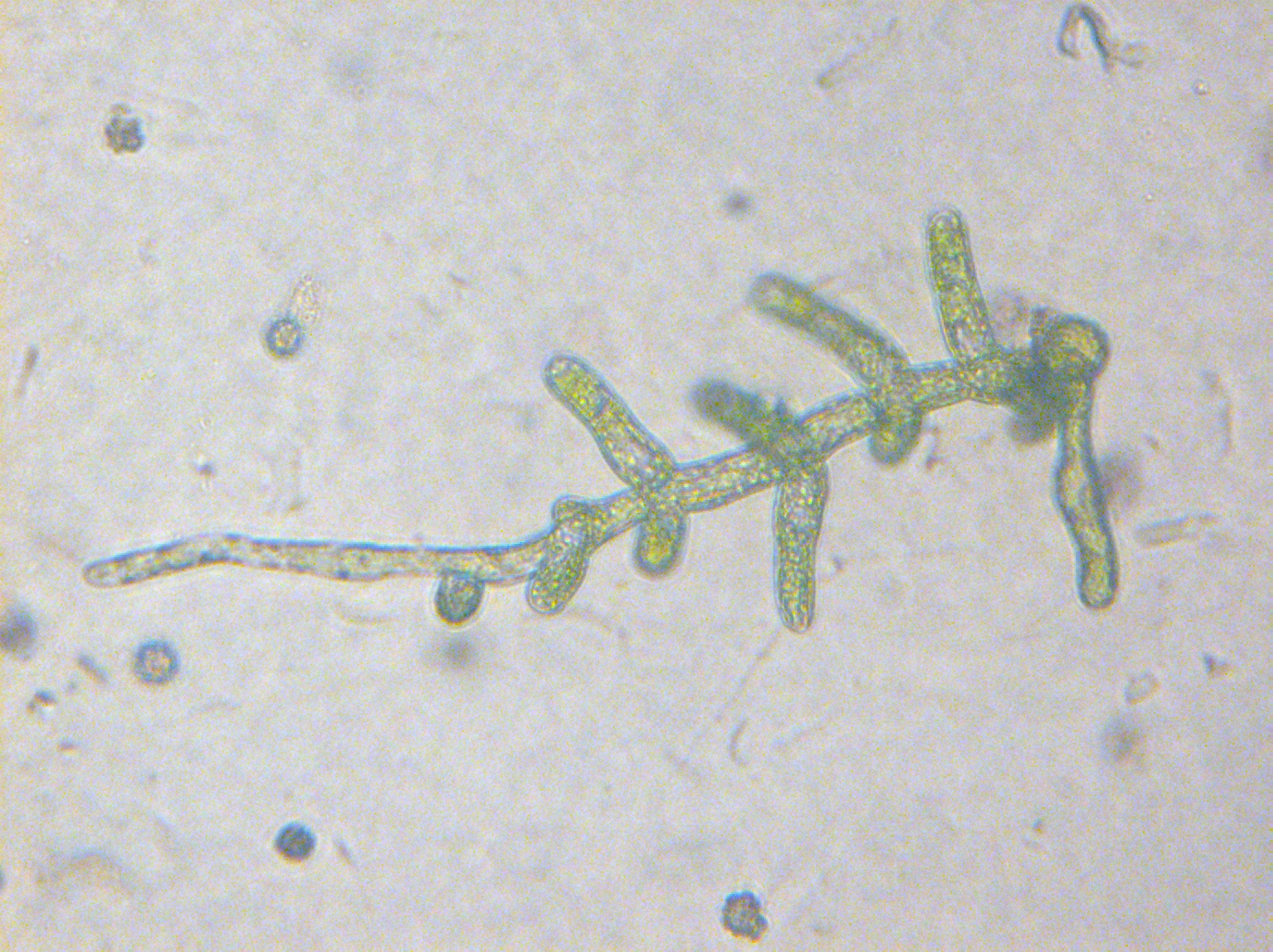

Supplement: Supplementary file 4 — Source Data [file 41467_2020_15967_MOESM4_ESM.zip › Raw data/Raw data for Figures/Figure 5a WT 13d.jpg]

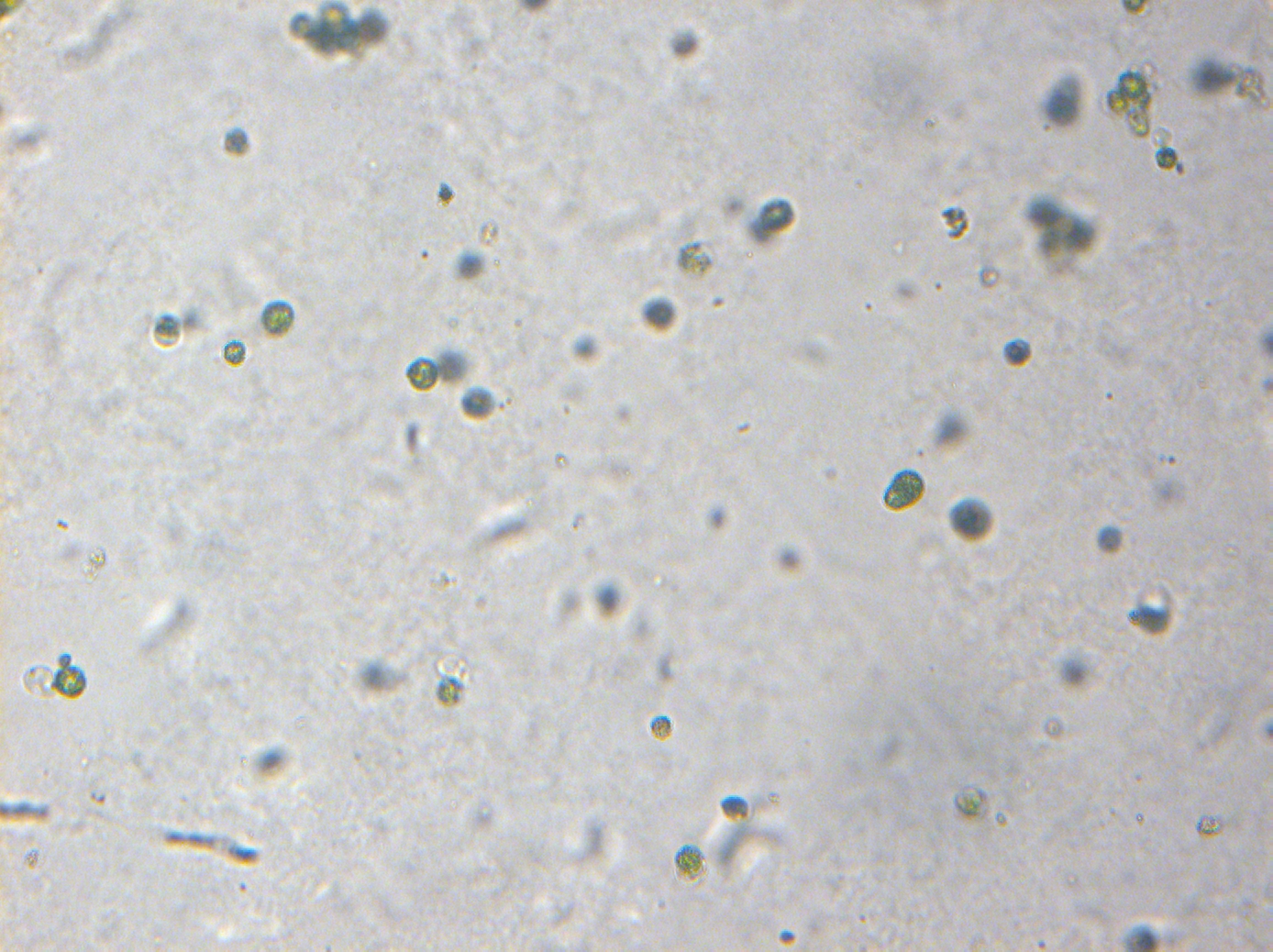

Supplement: Supplementary file 4 — Source Data [file 41467_2020_15967_MOESM4_ESM.zip › Raw data/Raw data for Figures/Figure 5a WT 2d.jpg]

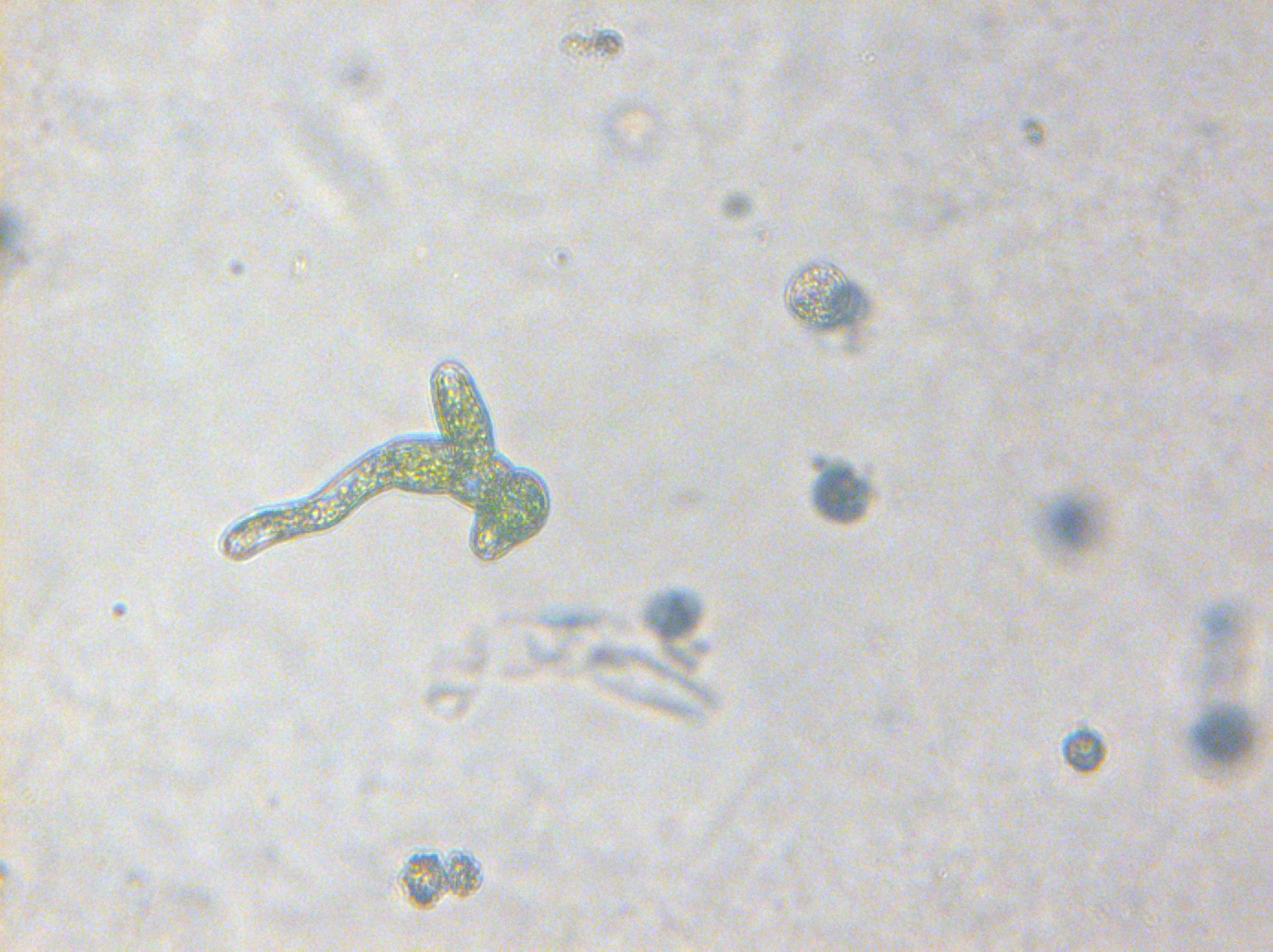

Supplement: Supplementary file 4 — Source Data [file 41467_2020_15967_MOESM4_ESM.zip › Raw data/Raw data for Figures/Figure 5a WT 5d.jpg]

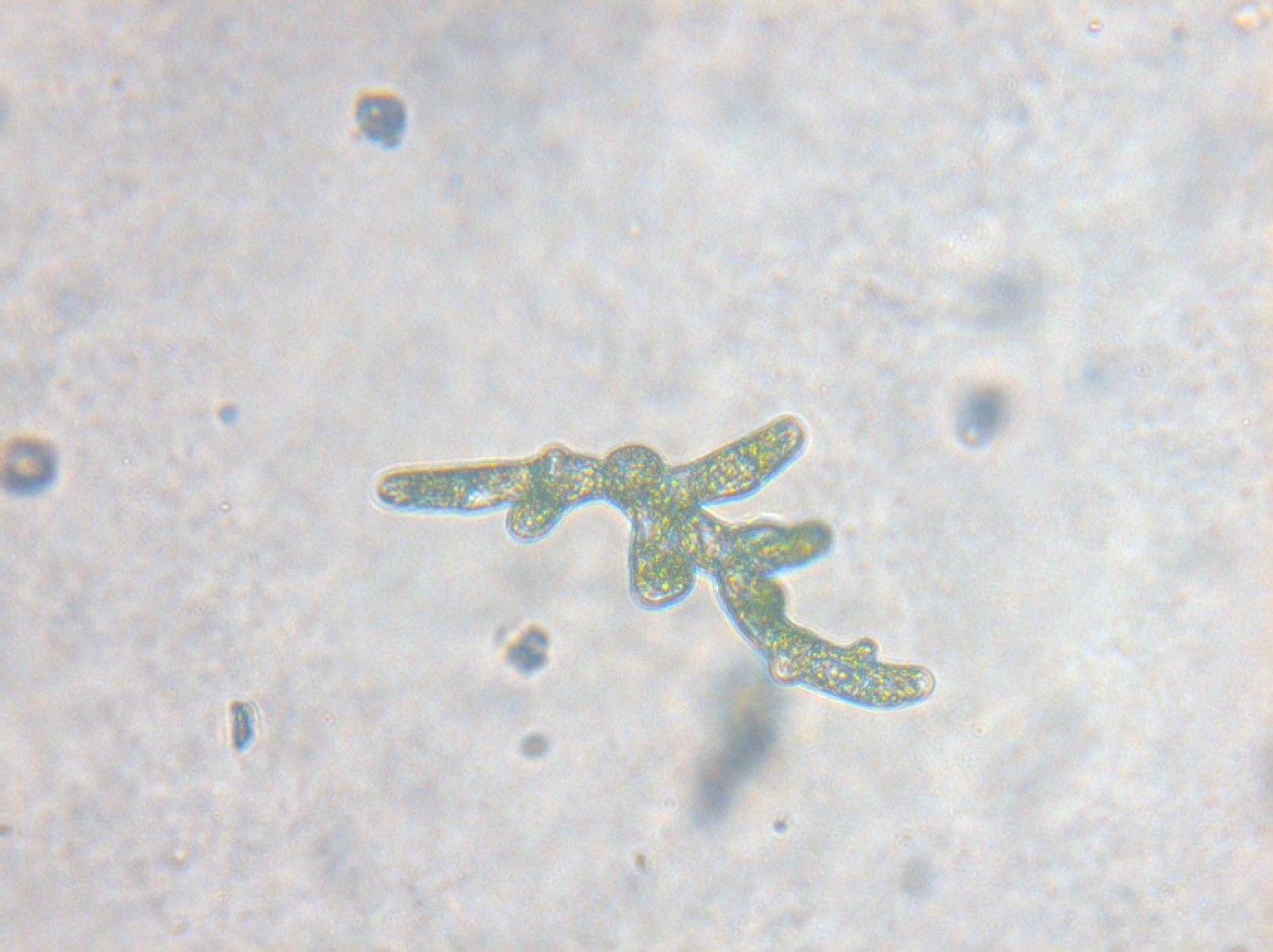

Supplement: Supplementary file 4 — Source Data [file 41467_2020_15967_MOESM4_ESM.zip › Raw data/Raw data for Figures/Figure 5a WT 7d.jpg]

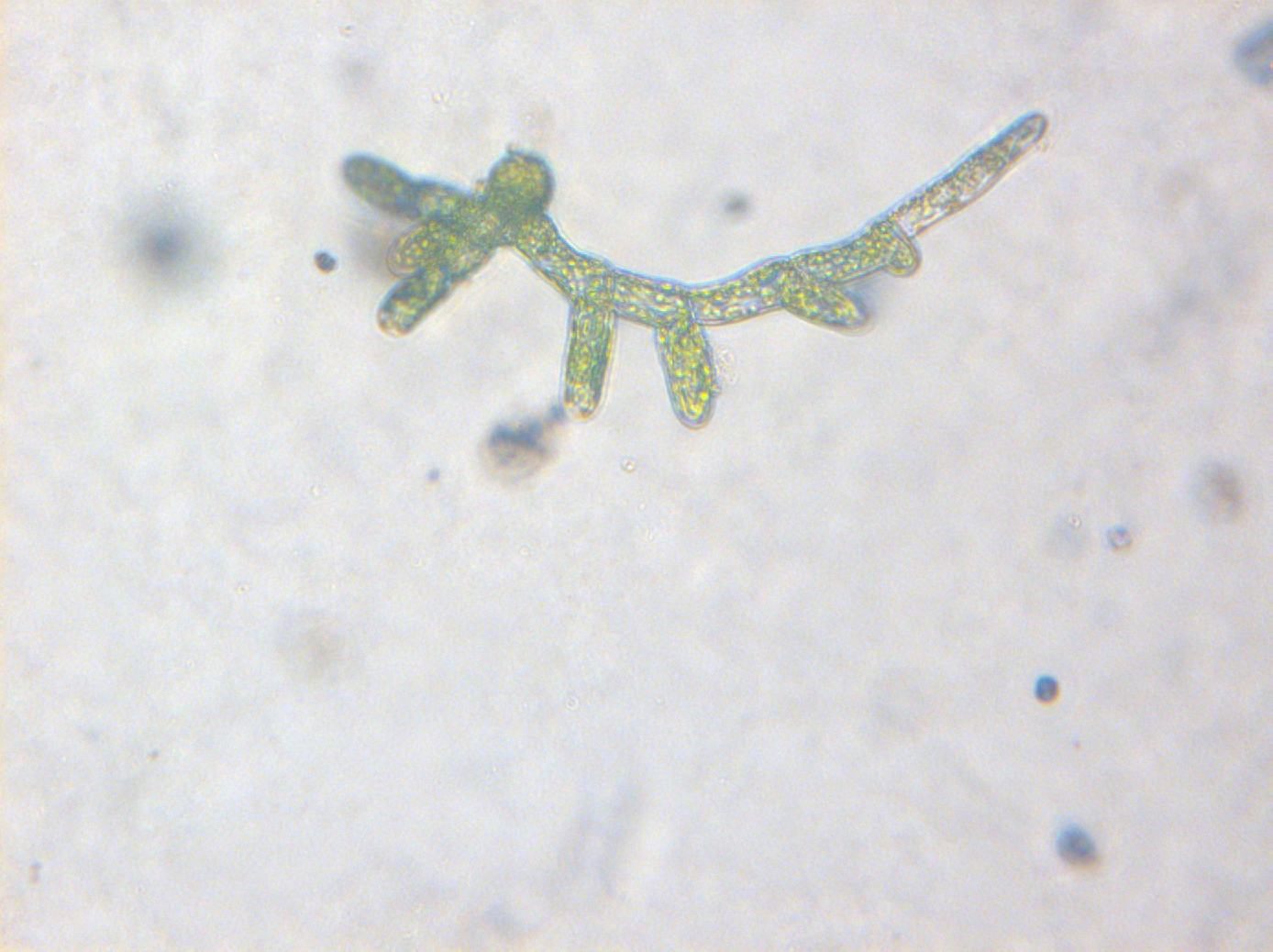

Supplement: Supplementary file 4 — Source Data [file 41467_2020_15967_MOESM4_ESM.zip › Raw data/Raw data for Figures/Figure 5a ppmacro2 #107 10d.jpg]

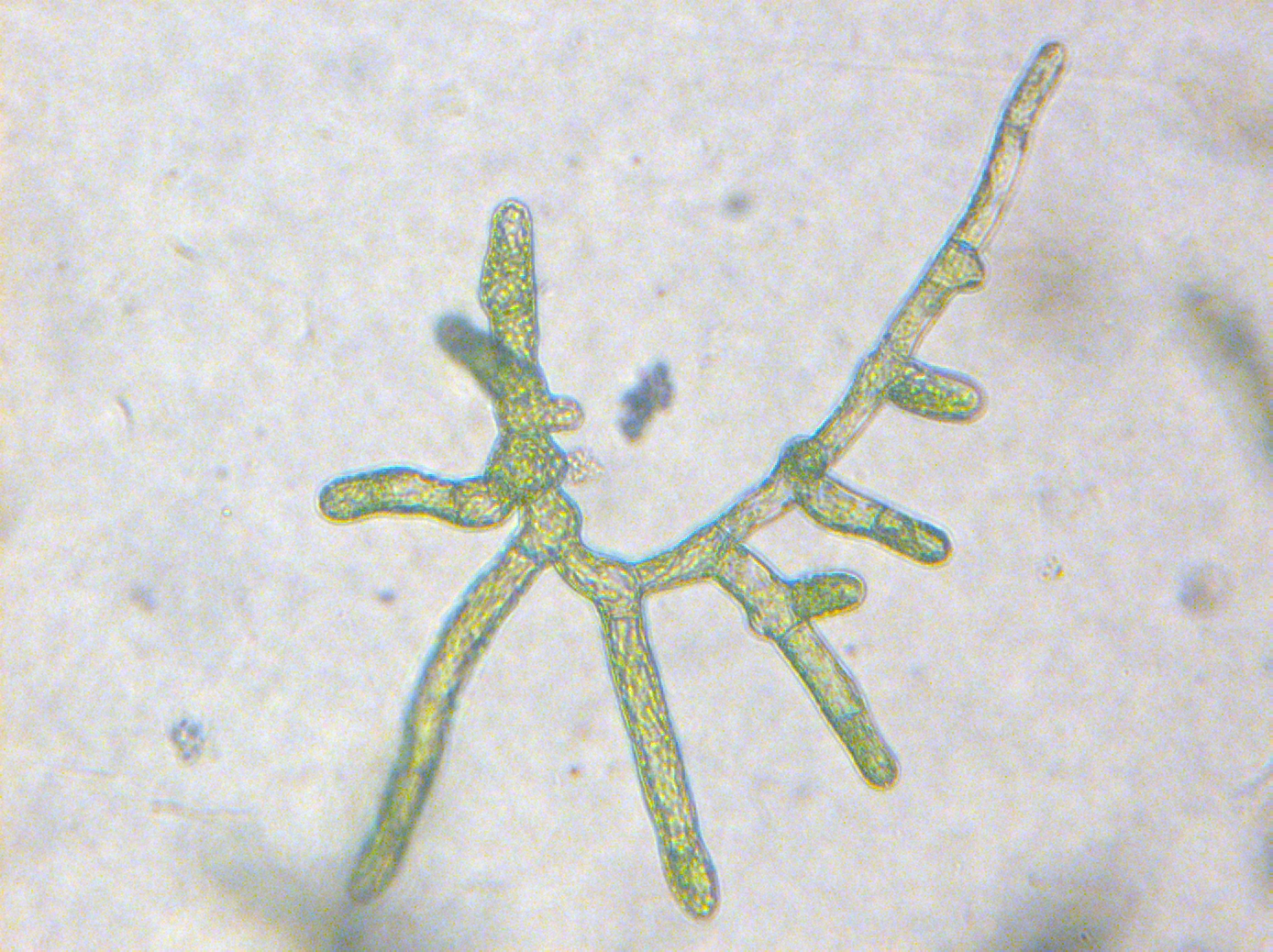

Supplement: Supplementary file 4 — Source Data [file 41467_2020_15967_MOESM4_ESM.zip › Raw data/Raw data for Figures/Figure 5a ppmacro2 #107 13d.jpg]

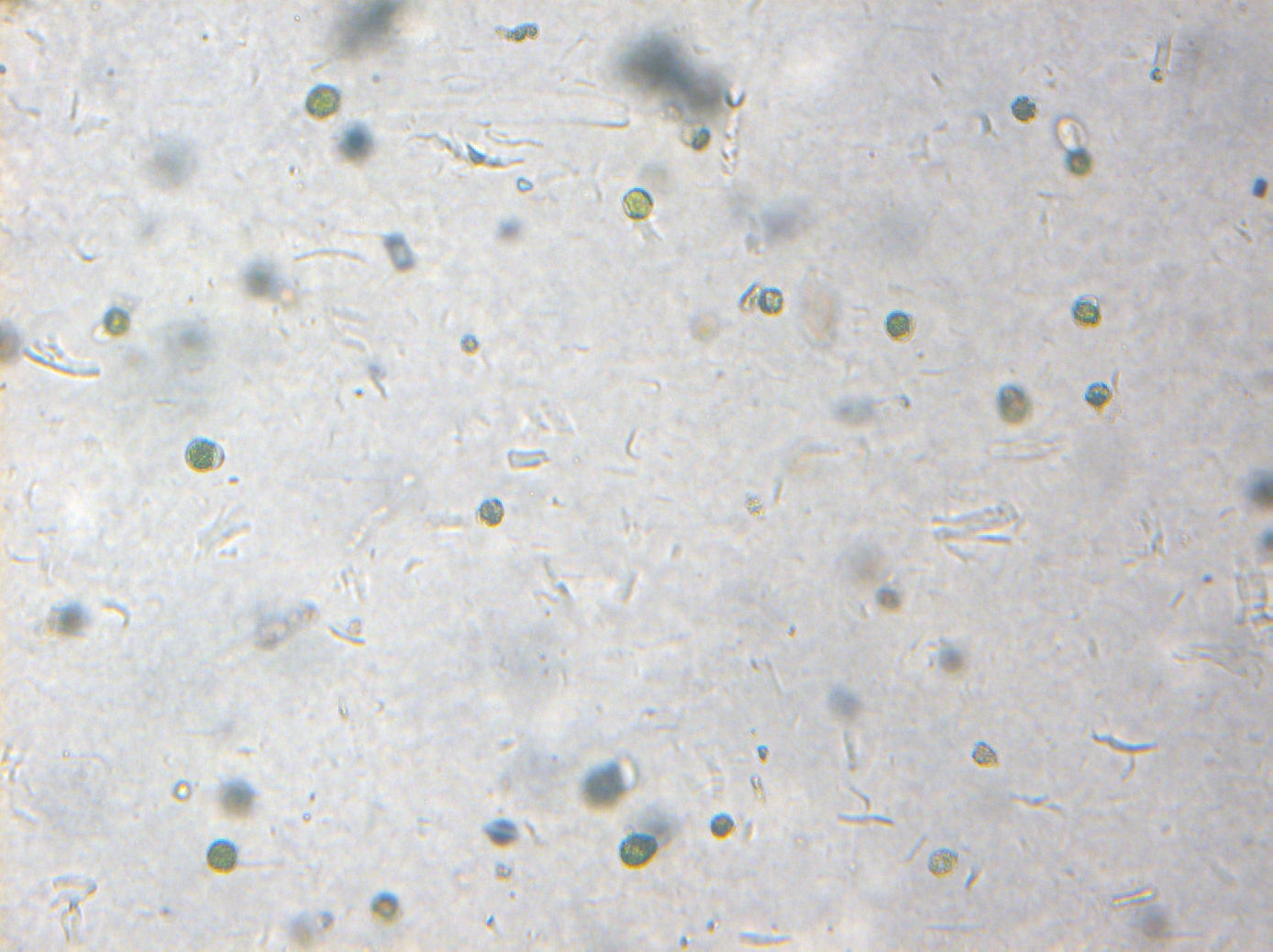

Supplement: Supplementary file 4 — Source Data [file 41467_2020_15967_MOESM4_ESM.zip › Raw data/Raw data for Figures/Figure 5a ppmacro2 #107 2d.jpg]

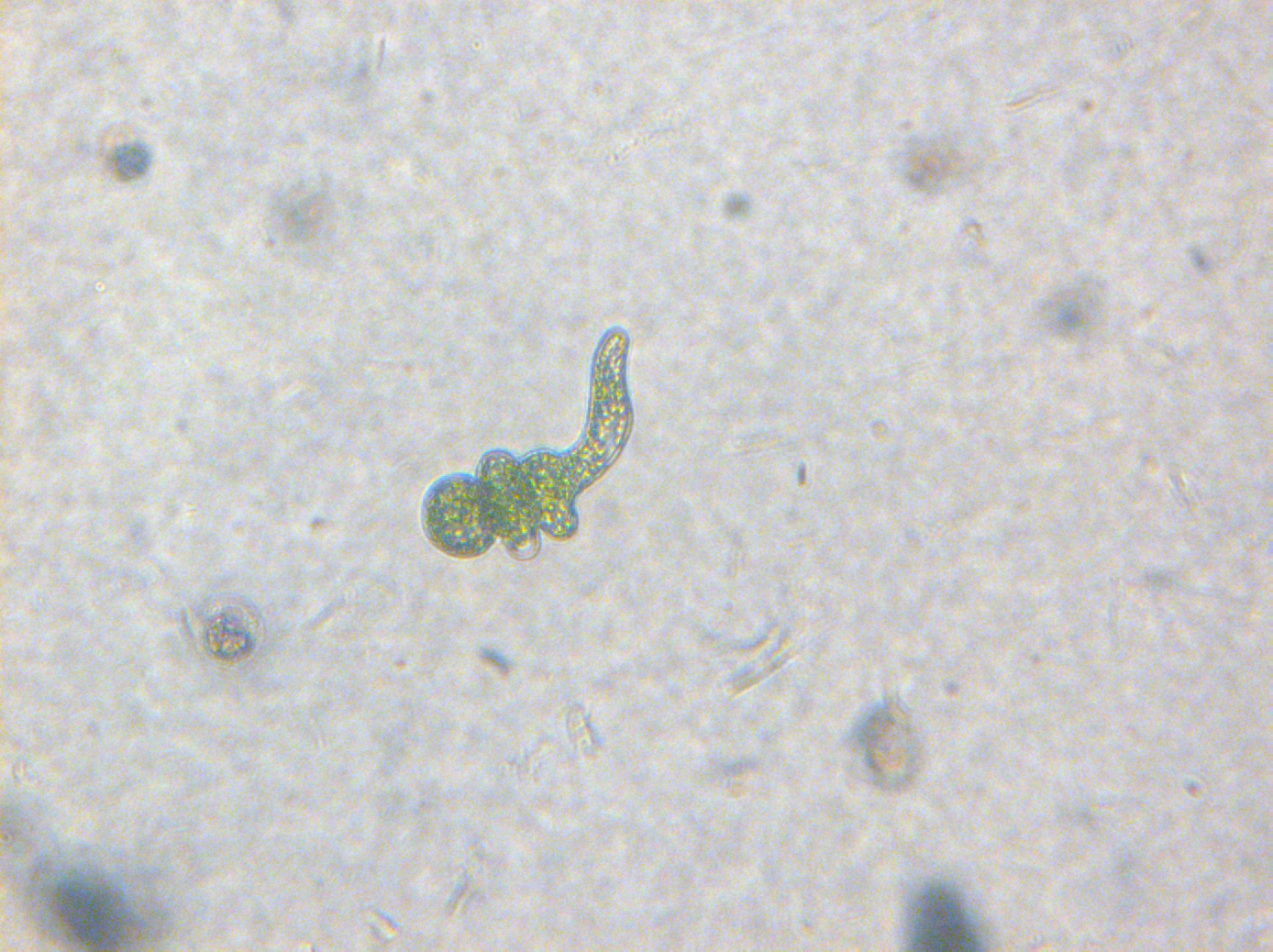

Supplement: Supplementary file 4 — Source Data [file 41467_2020_15967_MOESM4_ESM.zip › Raw data/Raw data for Figures/Figure 5a ppmacro2 #107 5d.jpg]

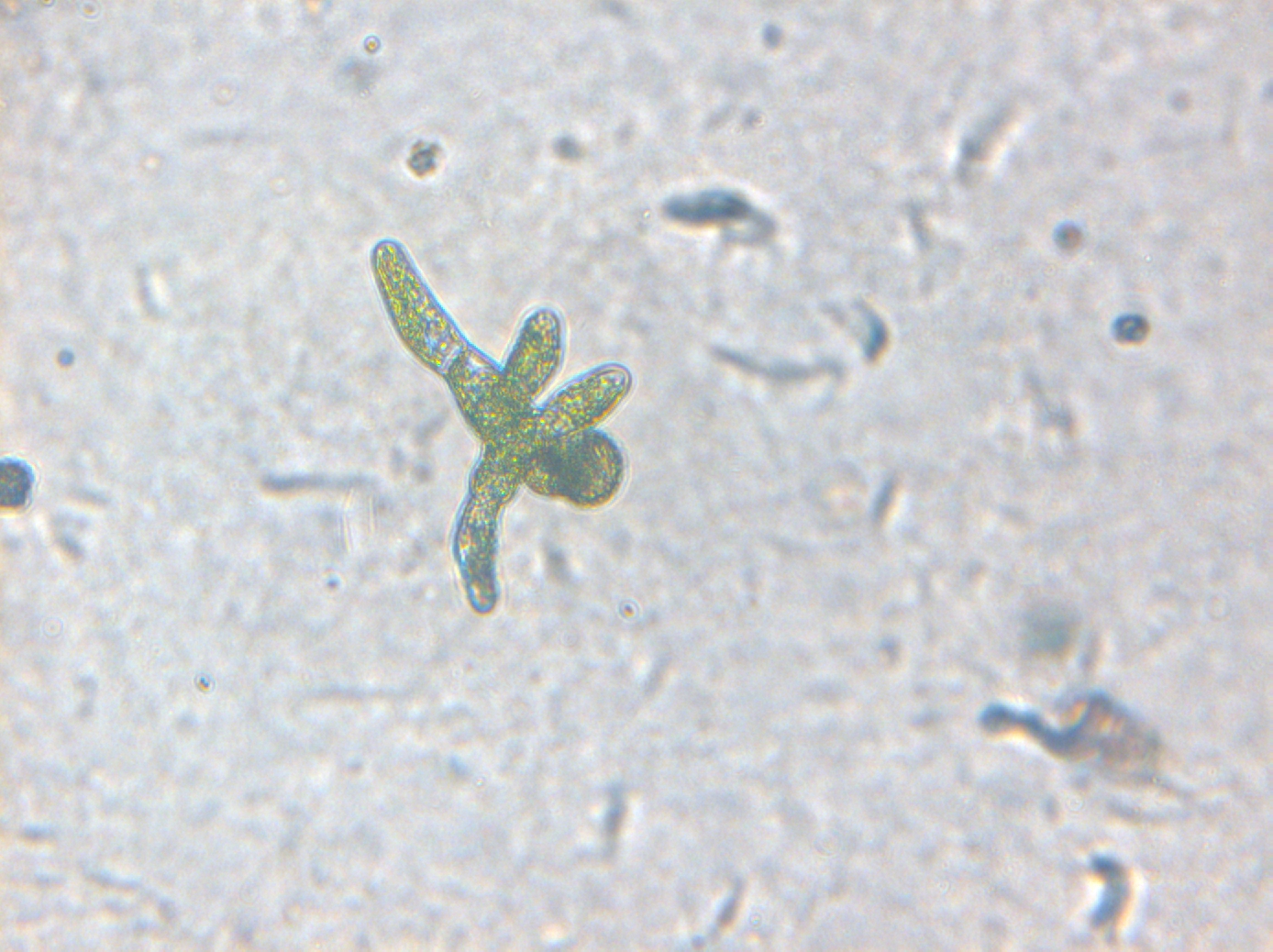

Supplement: Supplementary file 4 — Source Data [file 41467_2020_15967_MOESM4_ESM.zip › Raw data/Raw data for Figures/Figure 5a ppmacro2 #107 7d.jpg]

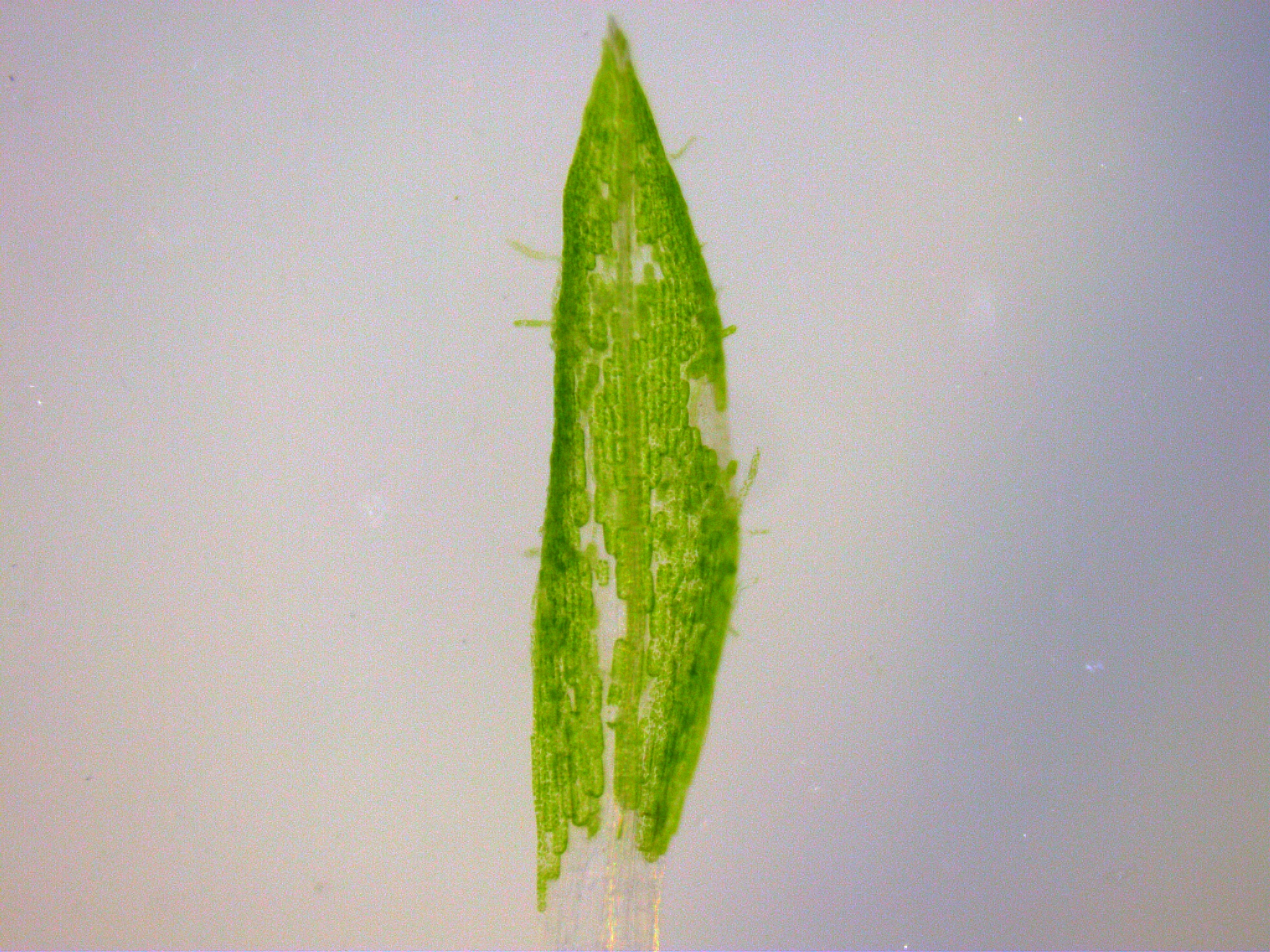

Supplement: Supplementary file 4 — Source Data [file 41467_2020_15967_MOESM4_ESM.zip › Raw data/Raw data for Figures/Figure 5b PpMACRO2-OE #48.tif]

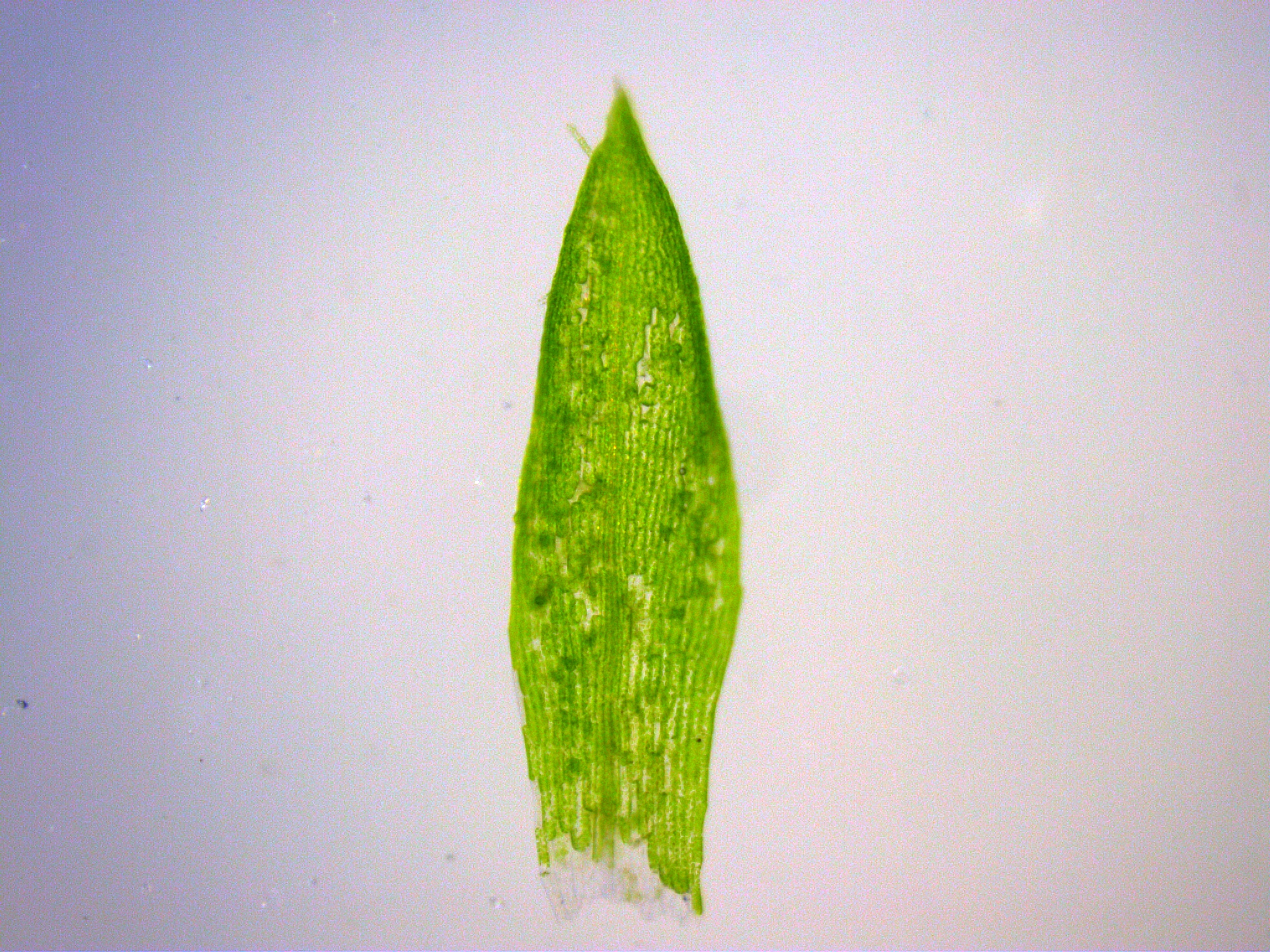

Supplement: Supplementary file 4 — Source Data [file 41467_2020_15967_MOESM4_ESM.zip › Raw data/Raw data for Figures/Figure 5b WT.tif]

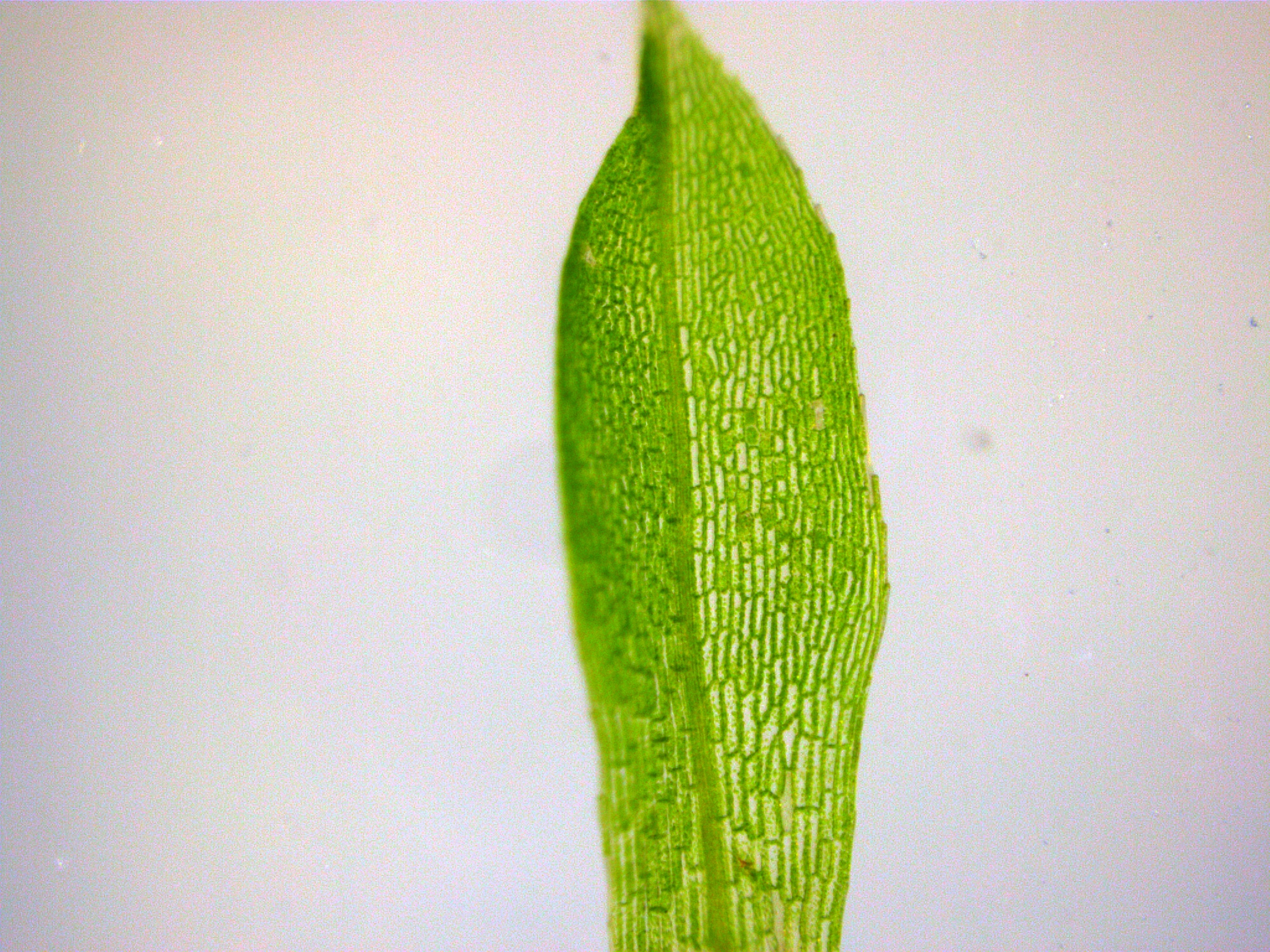

Supplement: Supplementary file 4 — Source Data [file 41467_2020_15967_MOESM4_ESM.zip › Raw data/Raw data for Figures/Figure 5b ppmacro2 #107.tif]

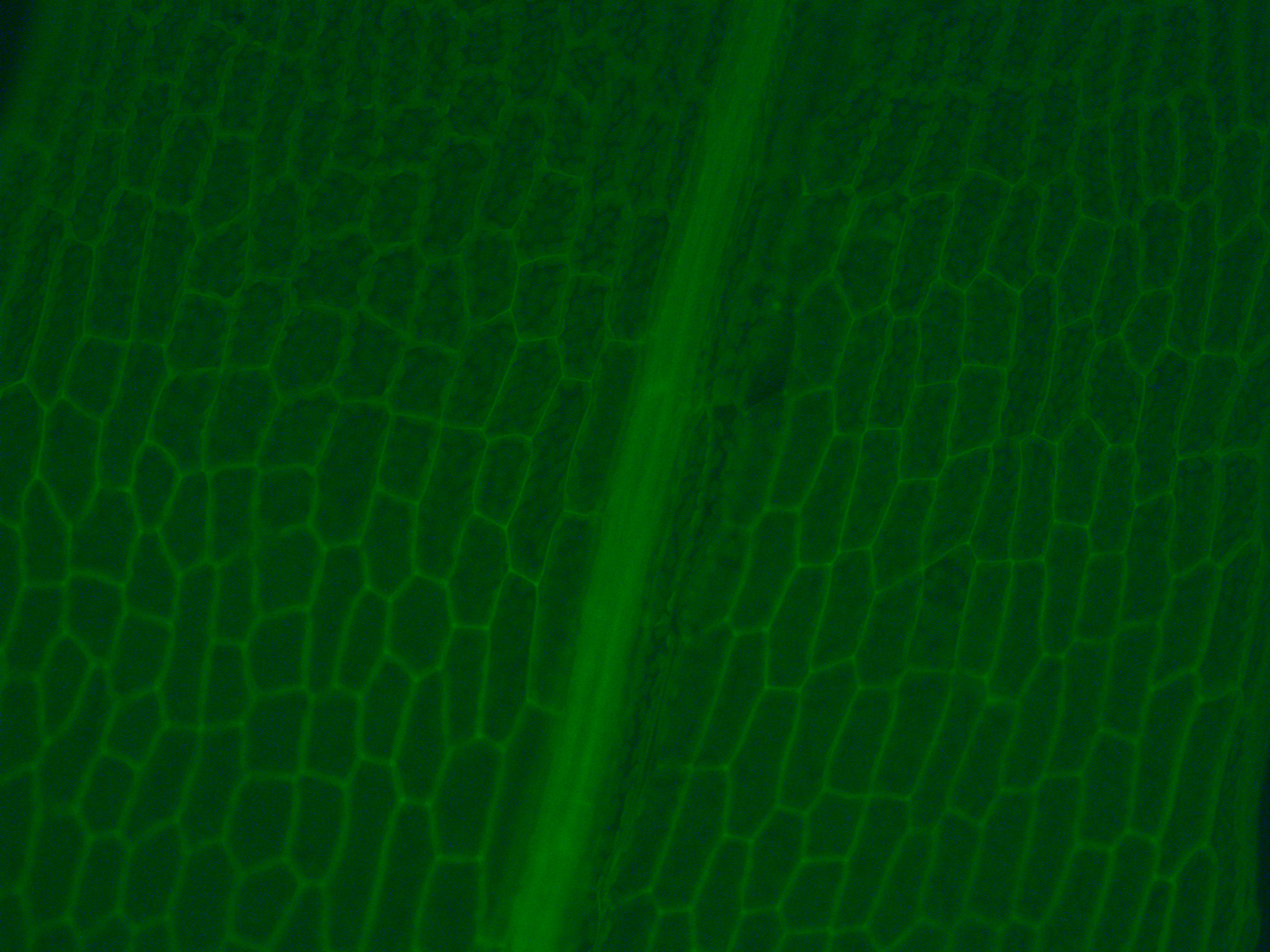

Supplement: Supplementary file 4 — Source Data [file 41467_2020_15967_MOESM4_ESM.zip › Raw data/Raw data for Figures/Figure 5c 0 h GFP.jpg]

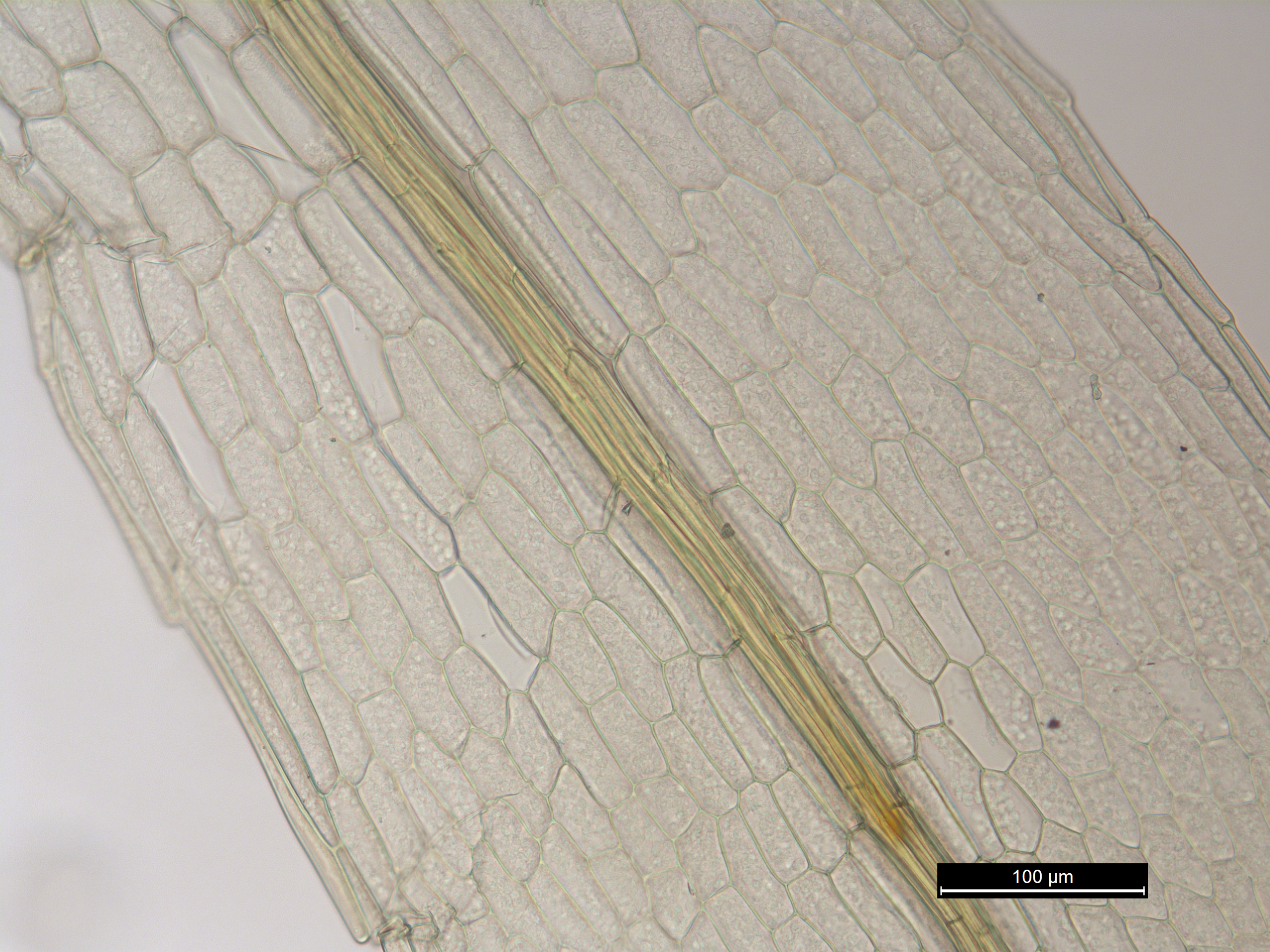

Supplement: Supplementary file 4 — Source Data [file 41467_2020_15967_MOESM4_ESM.zip › Raw data/Raw data for Figures/Figure 5c 0 h GUS.tif]

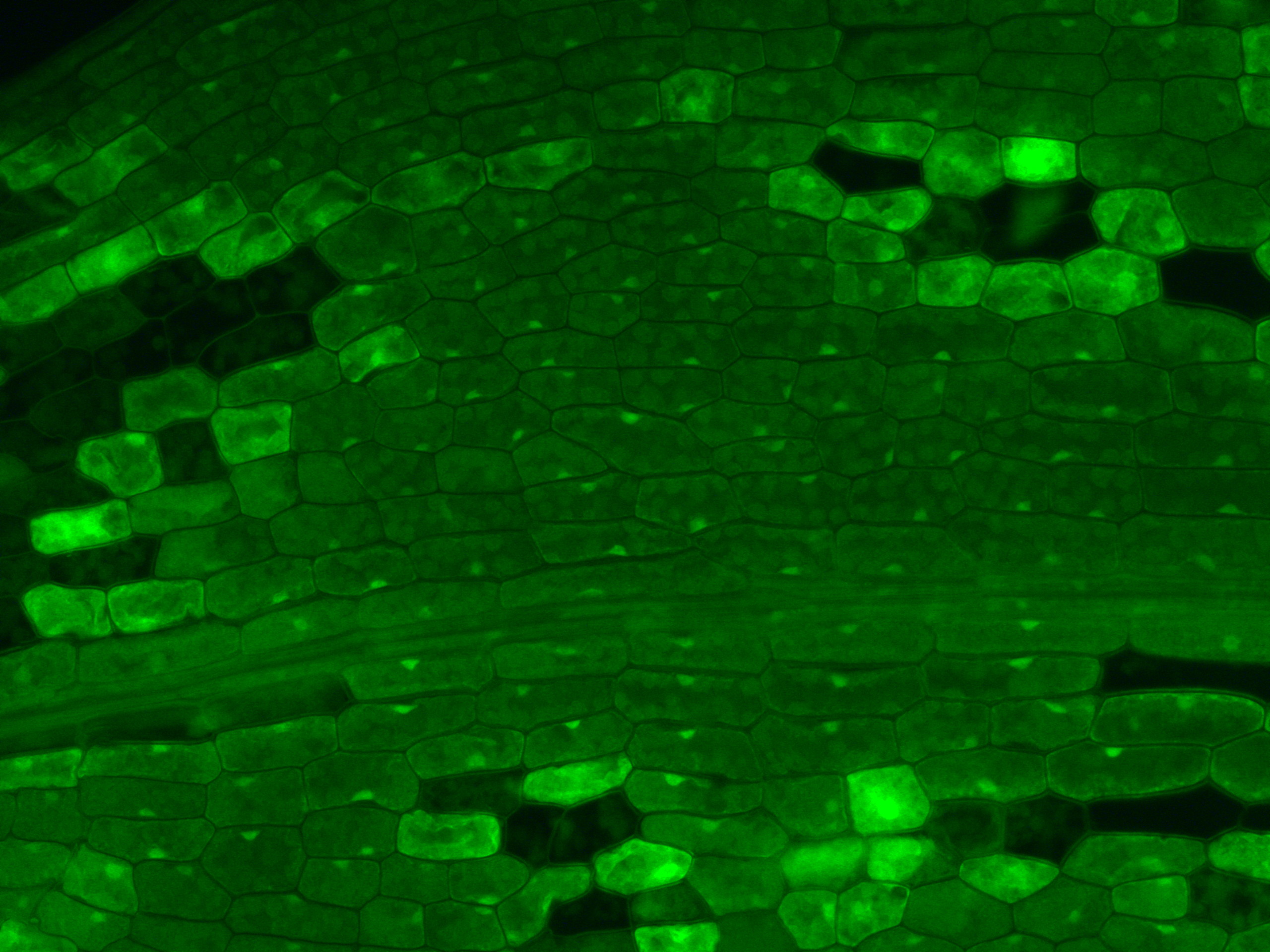

Supplement: Supplementary file 4 — Source Data [file 41467_2020_15967_MOESM4_ESM.zip › Raw data/Raw data for Figures/Figure 5c 48 h GFP.jpg]

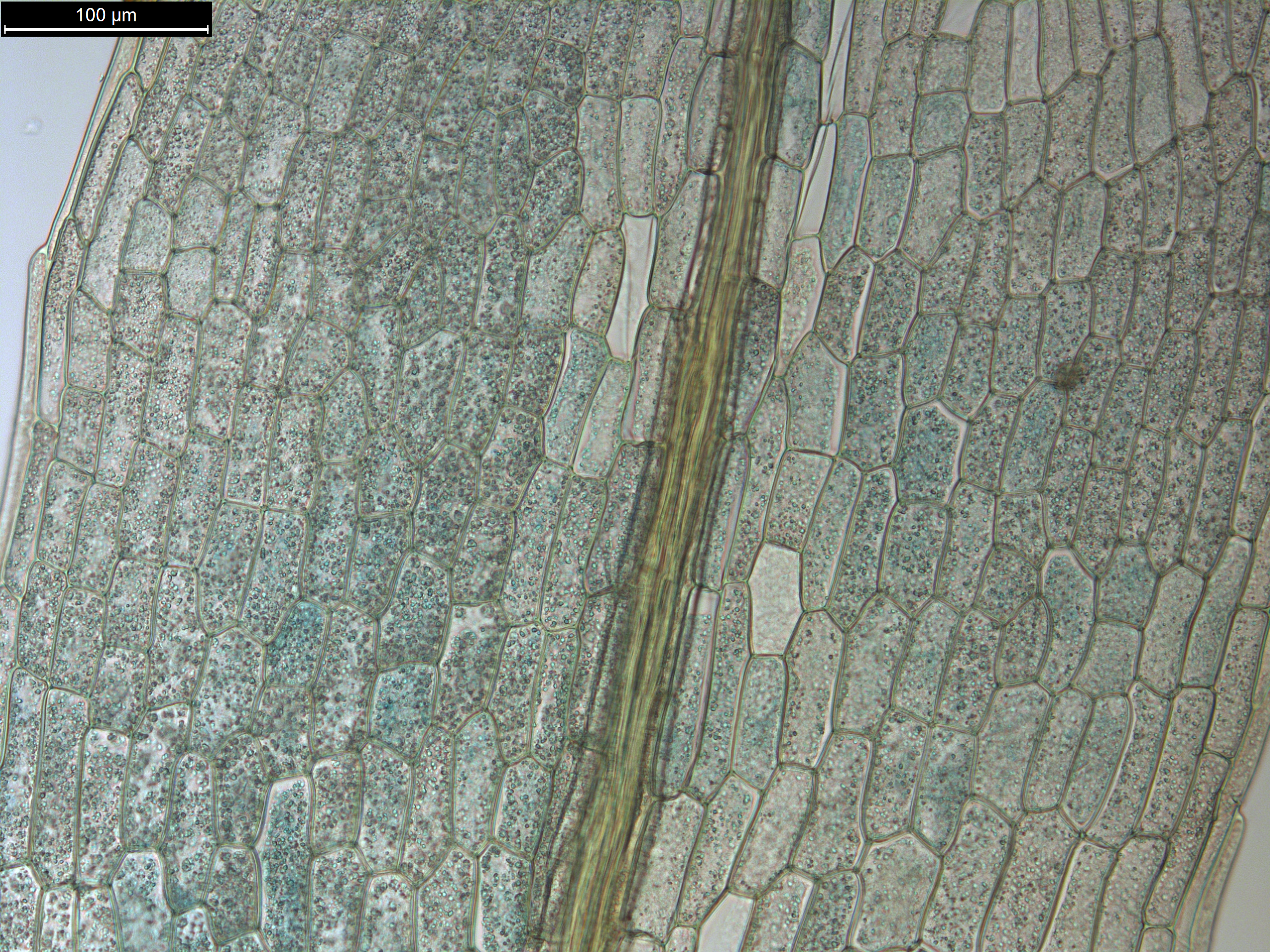

Supplement: Supplementary file 4 — Source Data [file 41467_2020_15967_MOESM4_ESM.zip › Raw data/Raw data for Figures/Figure 5c 48 h GUS.tif]

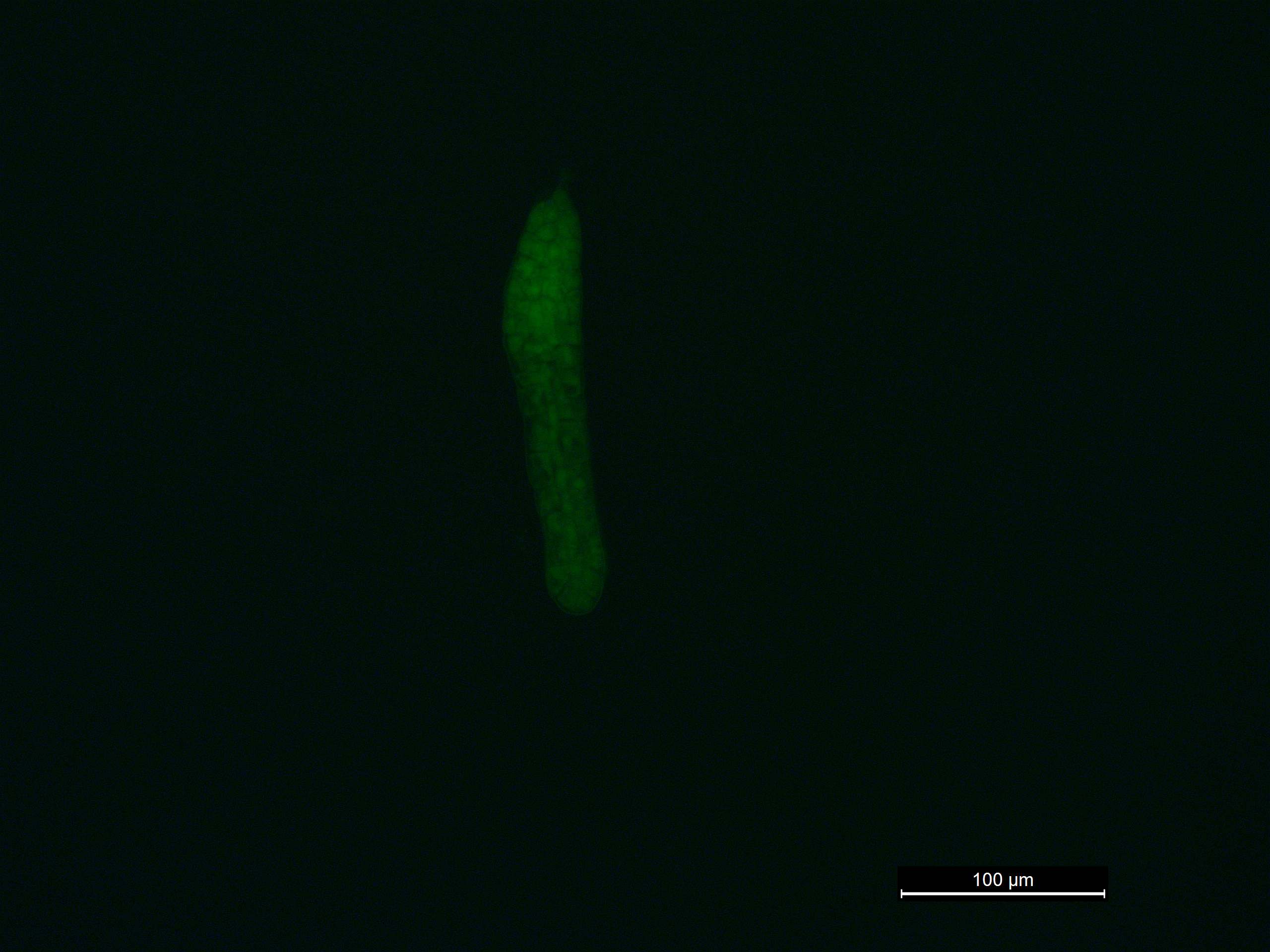

Supplement: Supplementary file 4 — Source Data [file 41467_2020_15967_MOESM4_ESM.zip › Raw data/Raw data for Supplementary Figures/Supplementary Fig 13 Archegonium.tif]

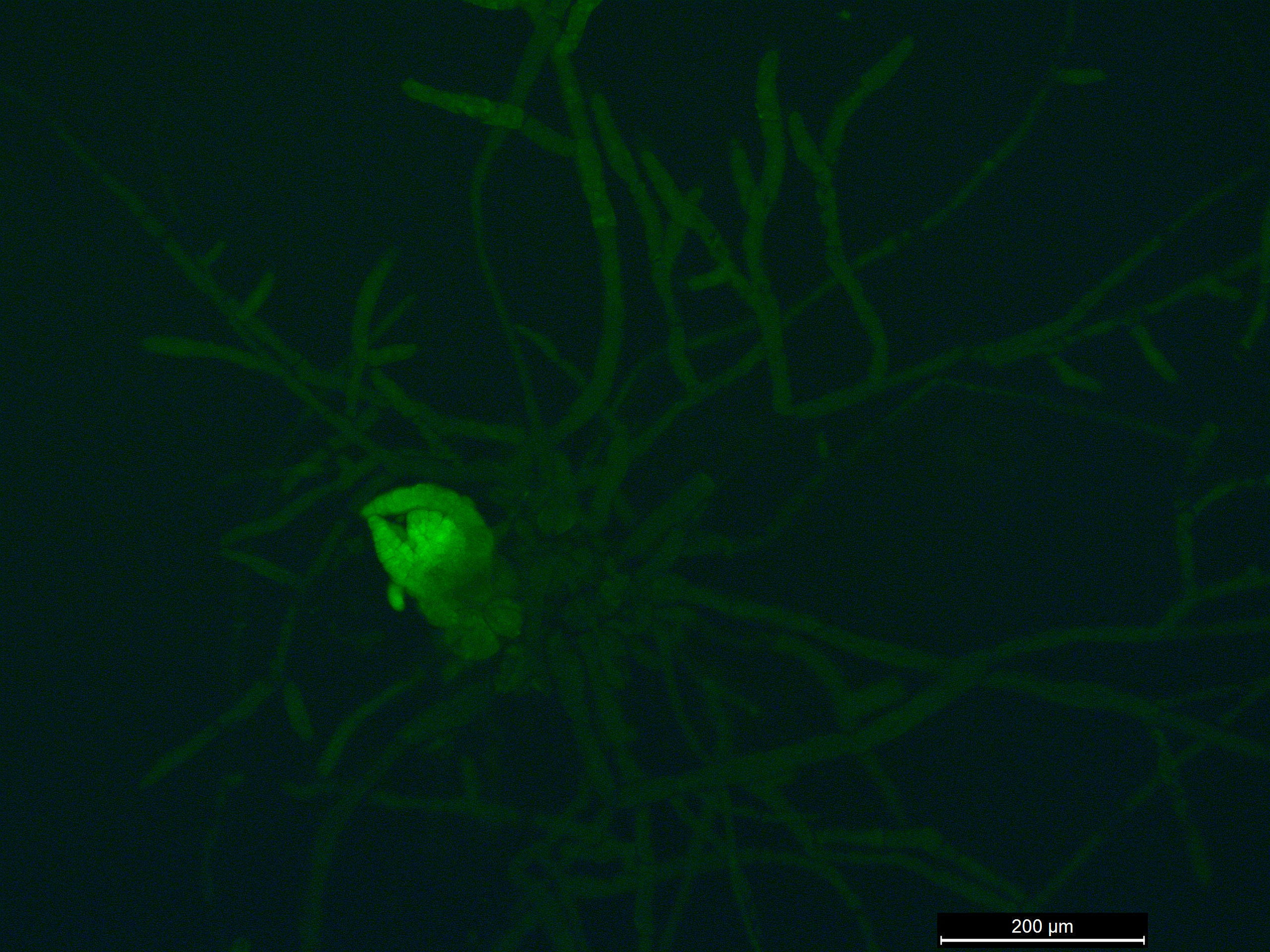

Supplement: Supplementary file 4 — Source Data [file 41467_2020_15967_MOESM4_ESM.zip › Raw data/Raw data for Supplementary Figures/Supplementary Fig 13 Gametophore.tif]

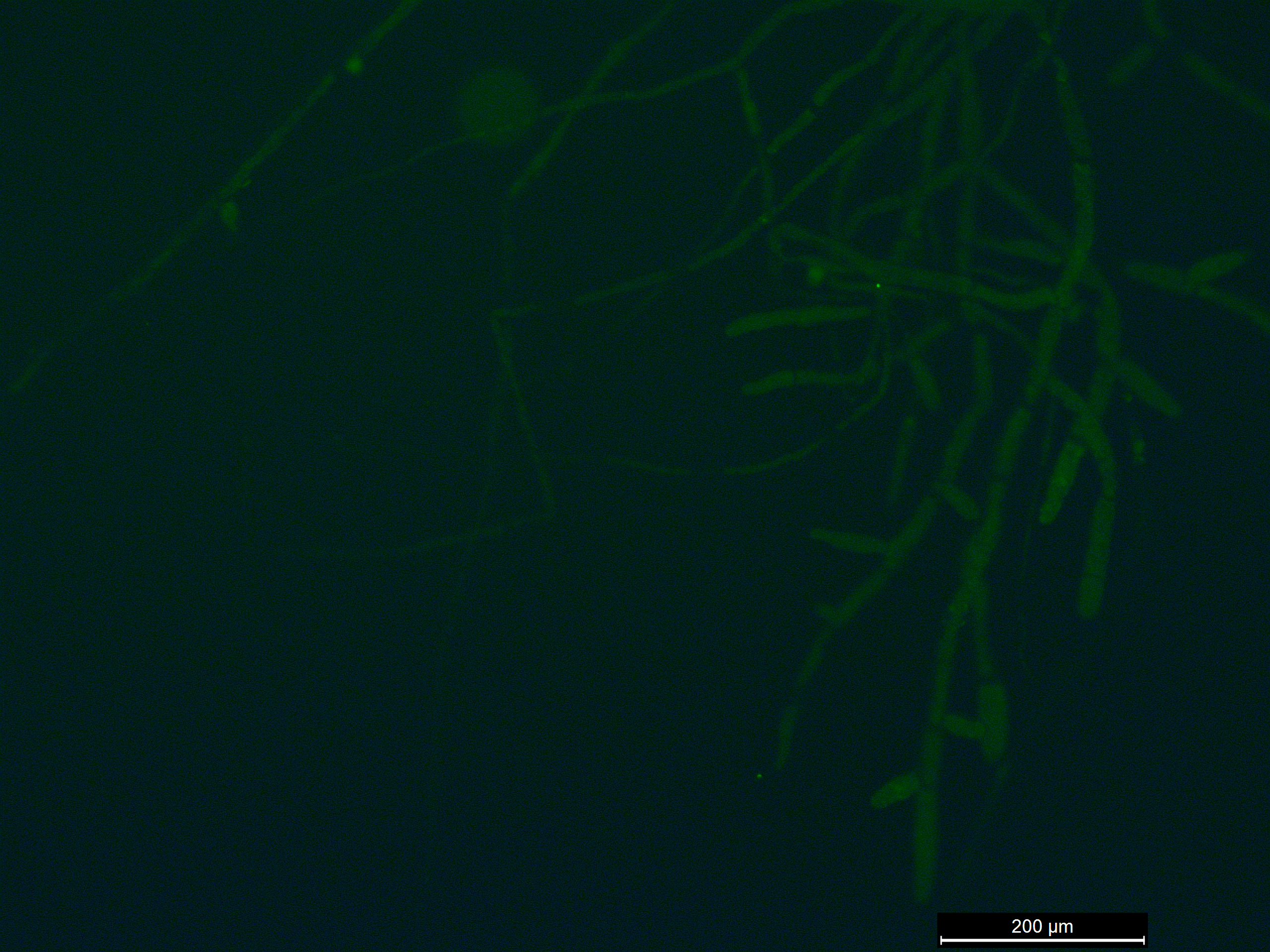

Supplement: Supplementary file 4 — Source Data [file 41467_2020_15967_MOESM4_ESM.zip › Raw data/Raw data for Supplementary Figures/Supplementary Fig 13 Protonemata.tif]

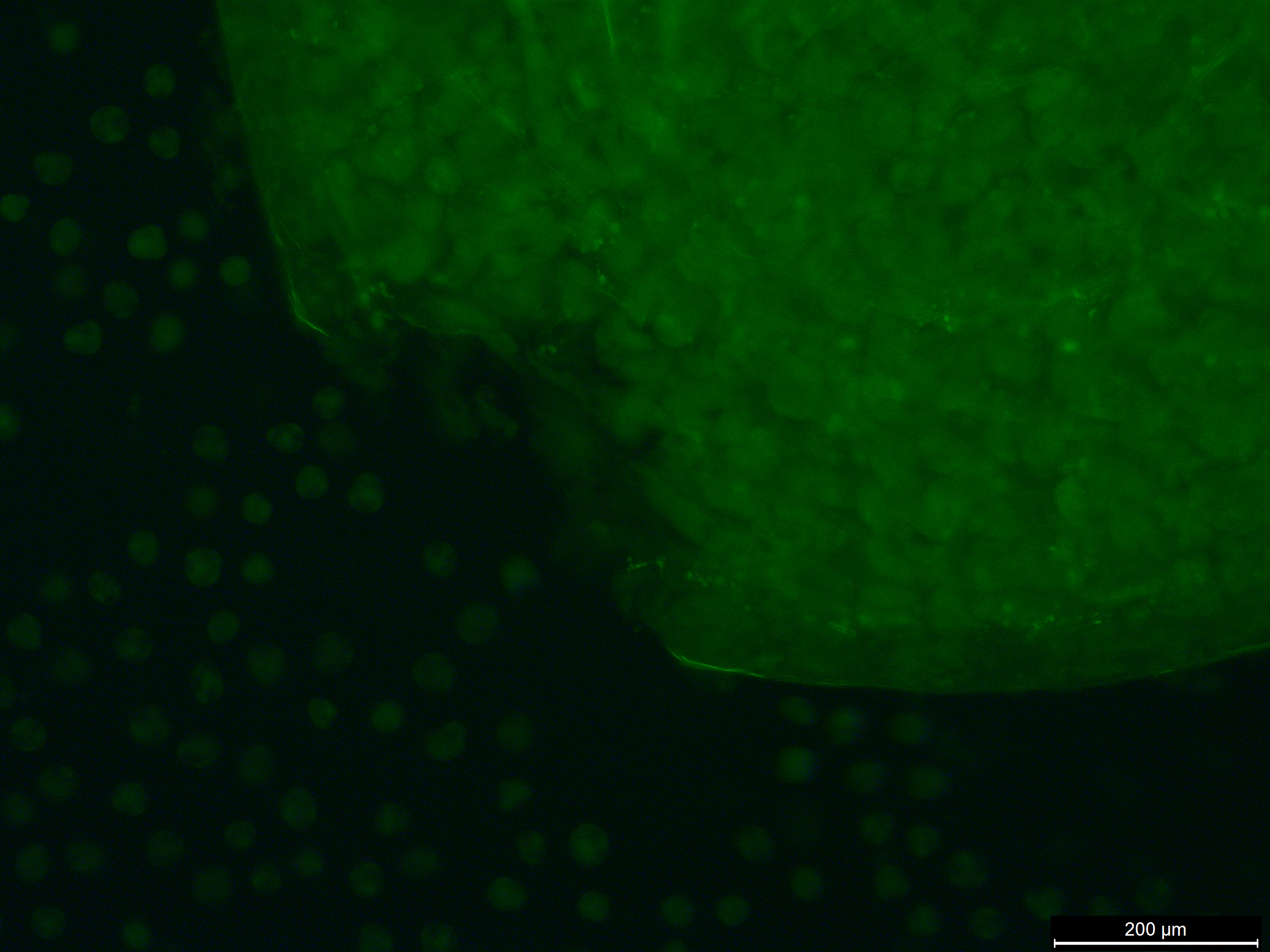

Supplement: Supplementary file 4 — Source Data [file 41467_2020_15967_MOESM4_ESM.zip › Raw data/Raw data for Supplementary Figures/Supplementary Fig 13 Spore.tif]

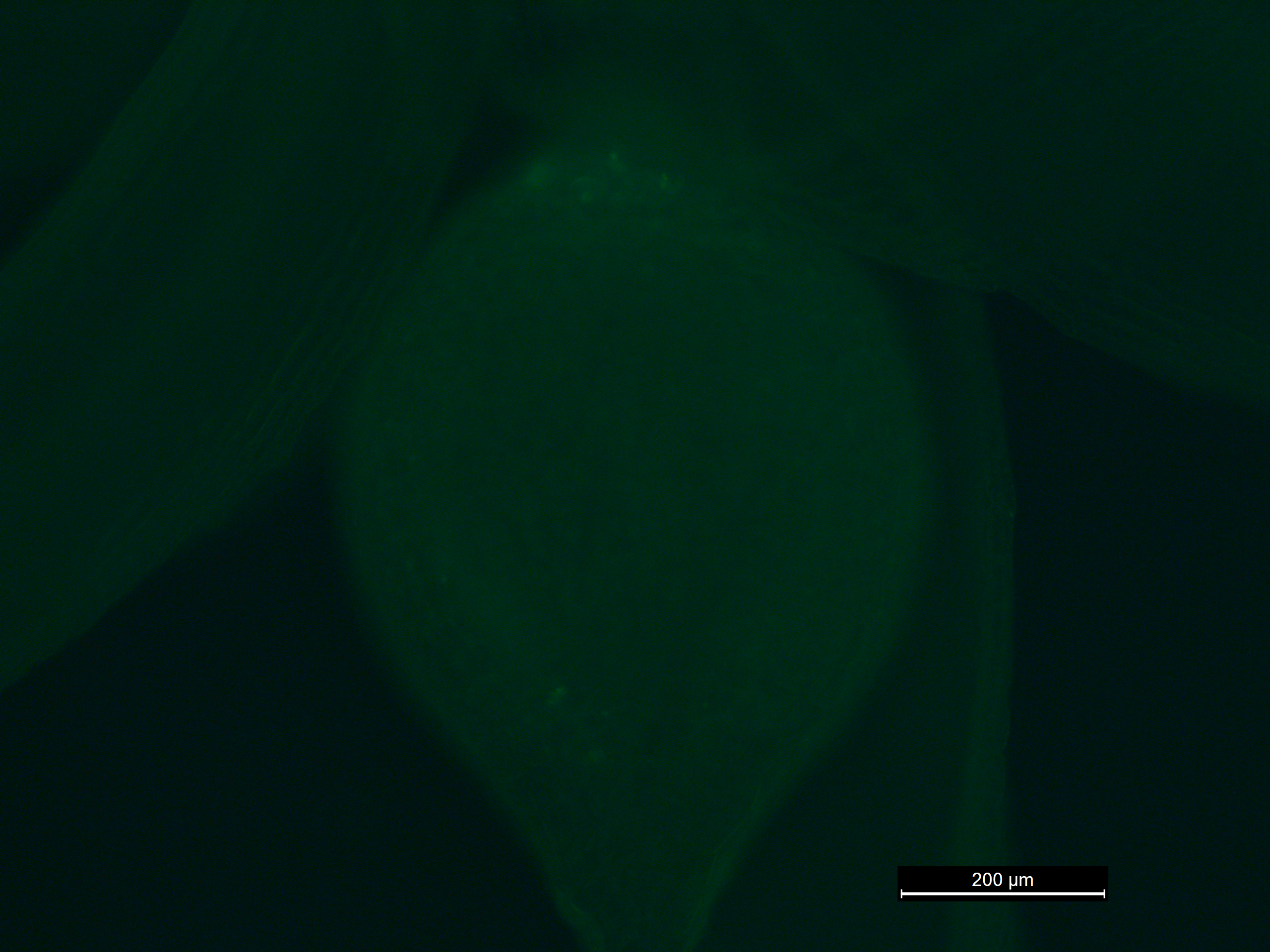

Supplement: Supplementary file 4 — Source Data [file 41467_2020_15967_MOESM4_ESM.zip › Raw data/Raw data for Supplementary Figures/Supplementary Fig 13 Sprorangium.tif]

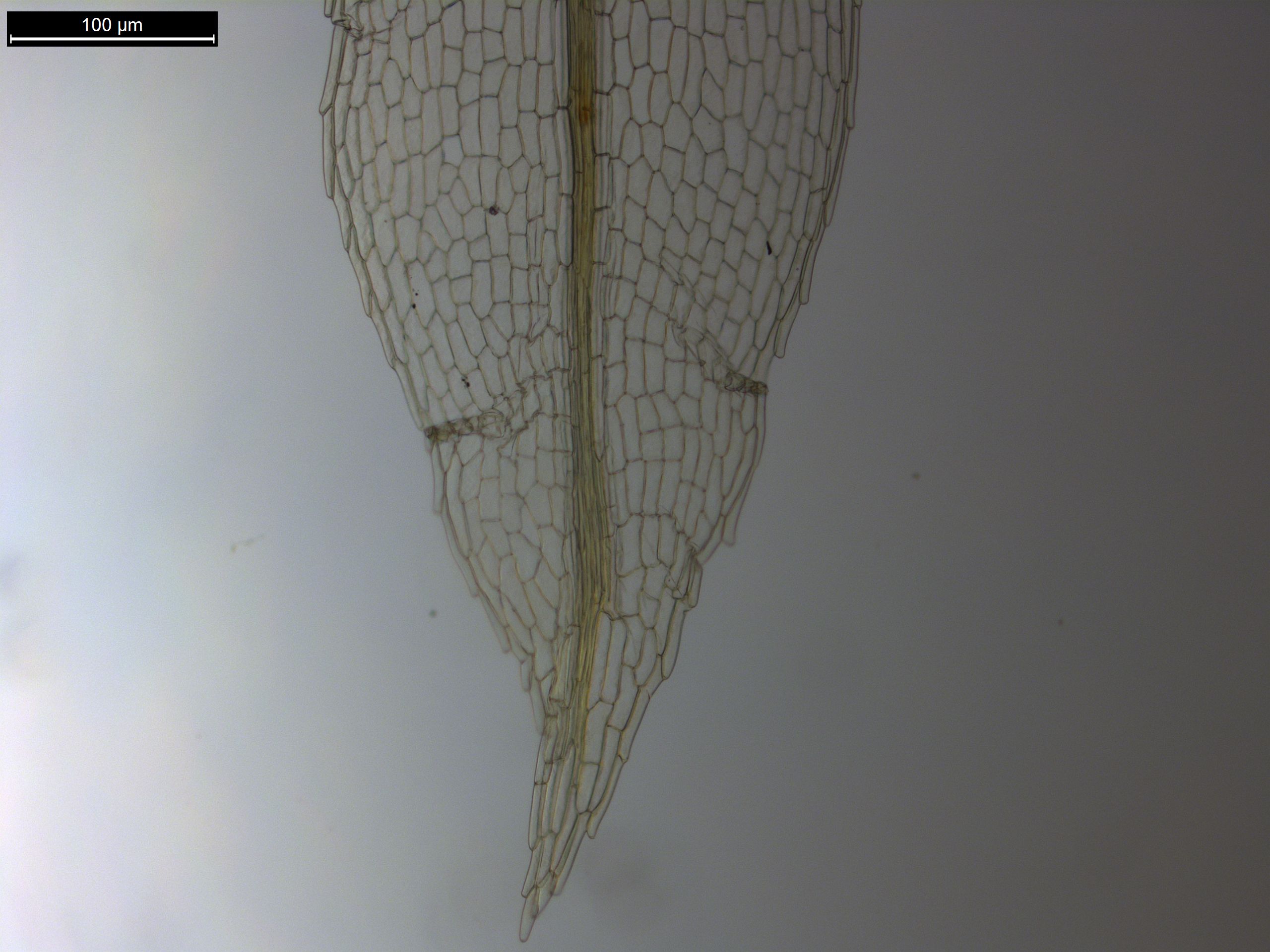

Supplement: Supplementary file 4 — Source Data [file 41467_2020_15967_MOESM4_ESM.zip › Raw data/Raw data for Supplementary Figures/Supplementary Fig 14a.tif]

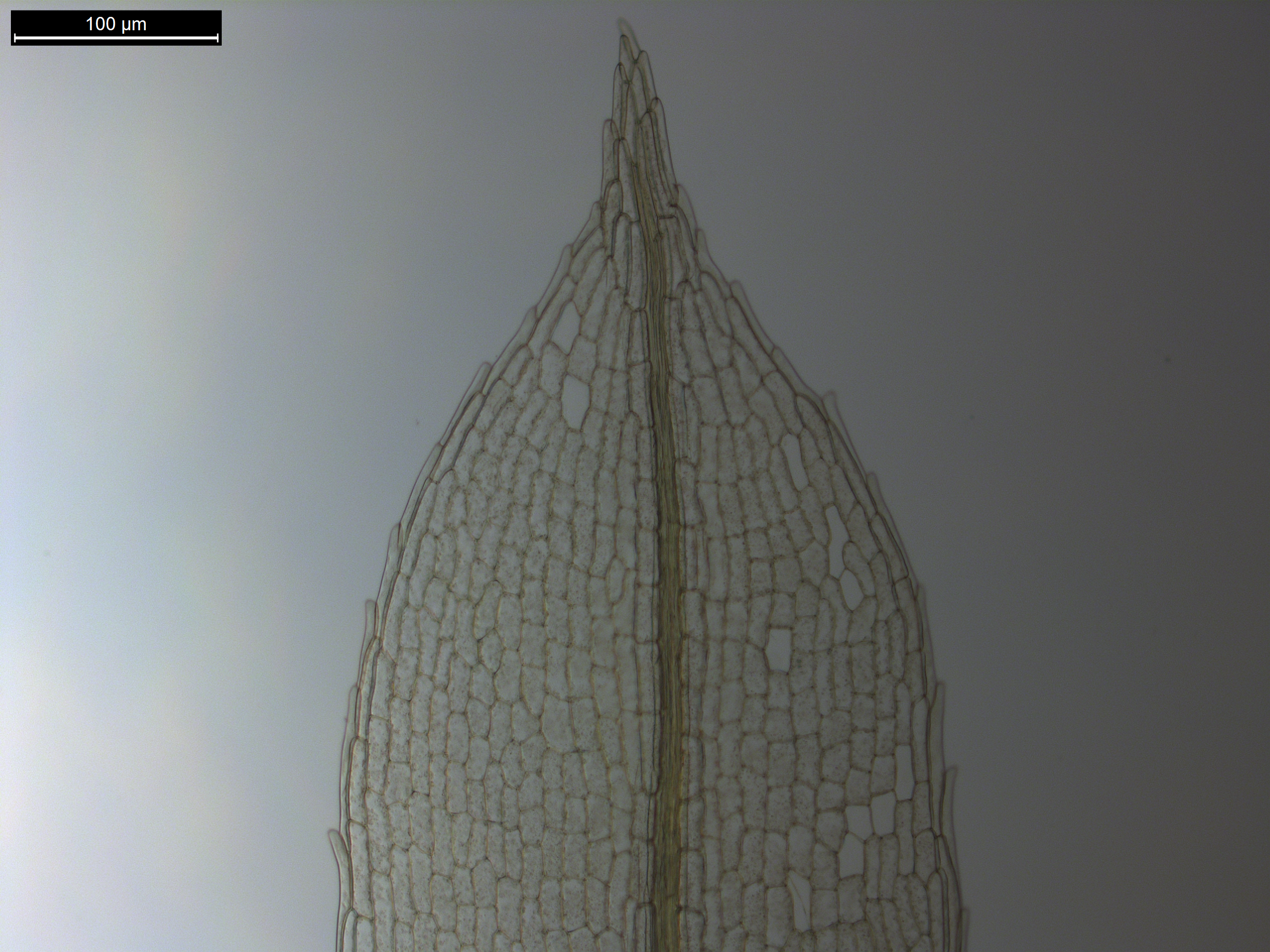

Supplement: Supplementary file 4 — Source Data [file 41467_2020_15967_MOESM4_ESM.zip › Raw data/Raw data for Supplementary Figures/Supplementary Fig 14b.tif]

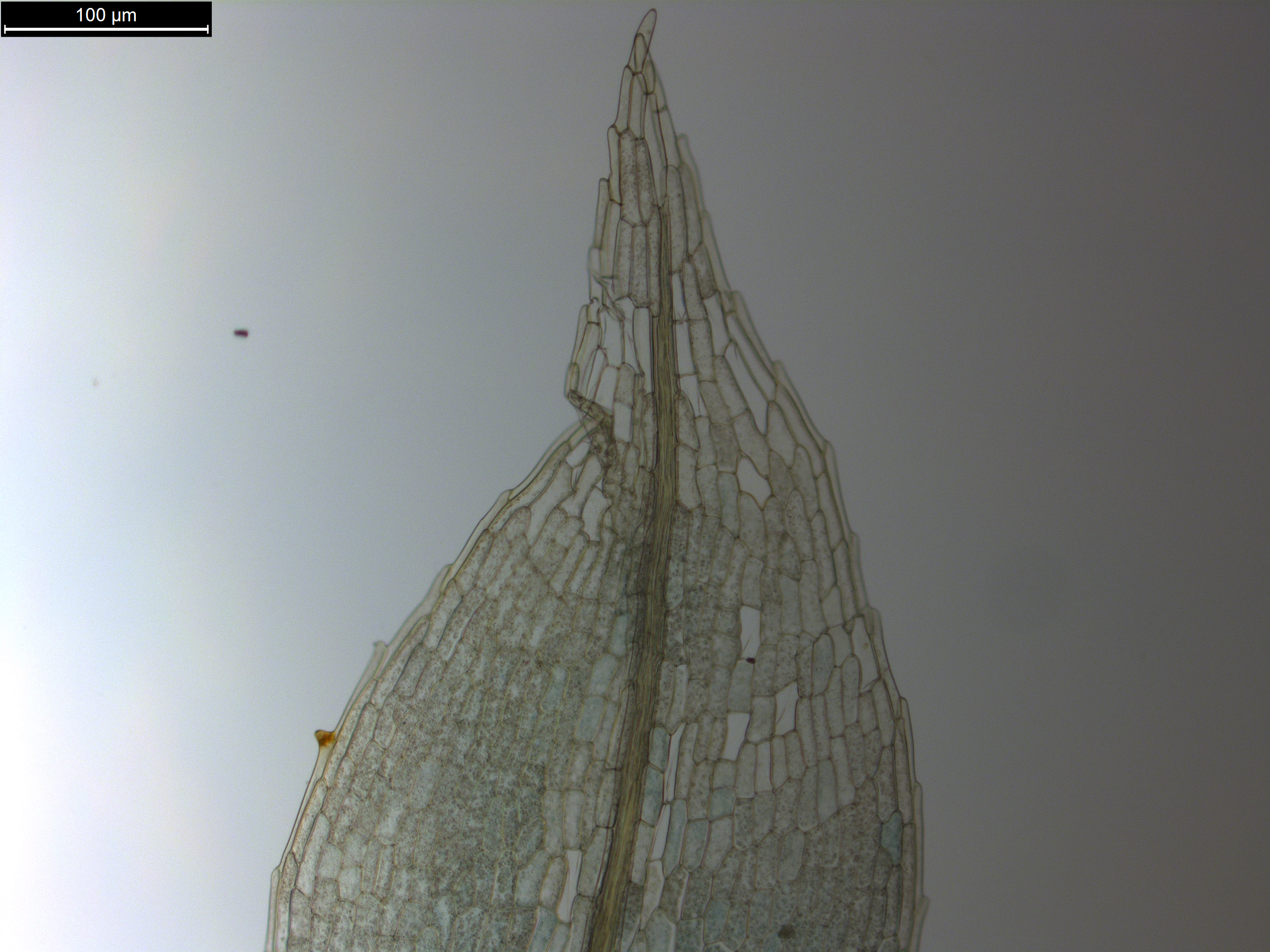

Supplement: Supplementary file 4 — Source Data [file 41467_2020_15967_MOESM4_ESM.zip › Raw data/Raw data for Supplementary Figures/Supplementary Fig 14c.tif]

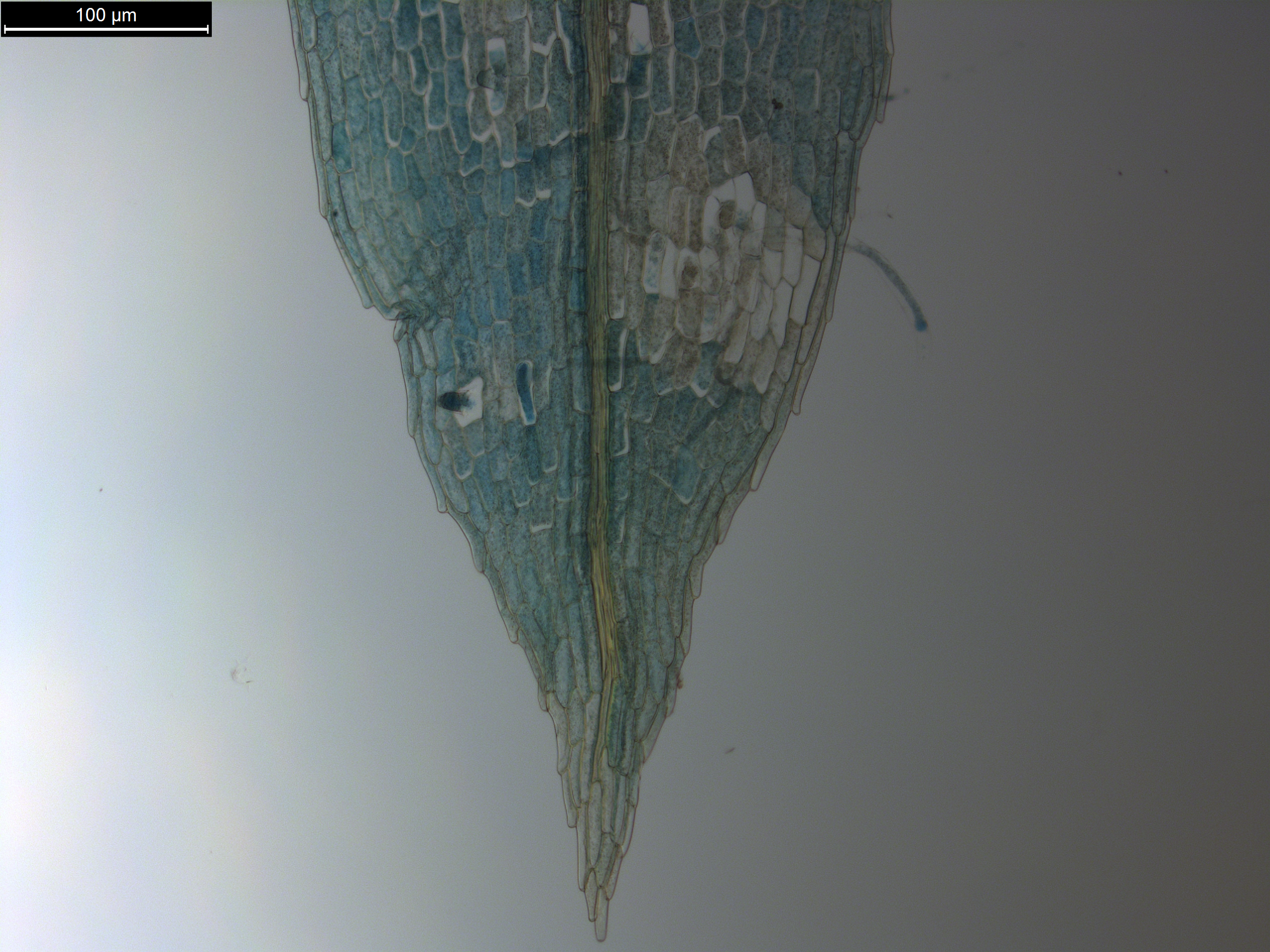

Supplement: Supplementary file 4 — Source Data [file 41467_2020_15967_MOESM4_ESM.zip › Raw data/Raw data for Supplementary Figures/Supplementary Fig 14d.tif]

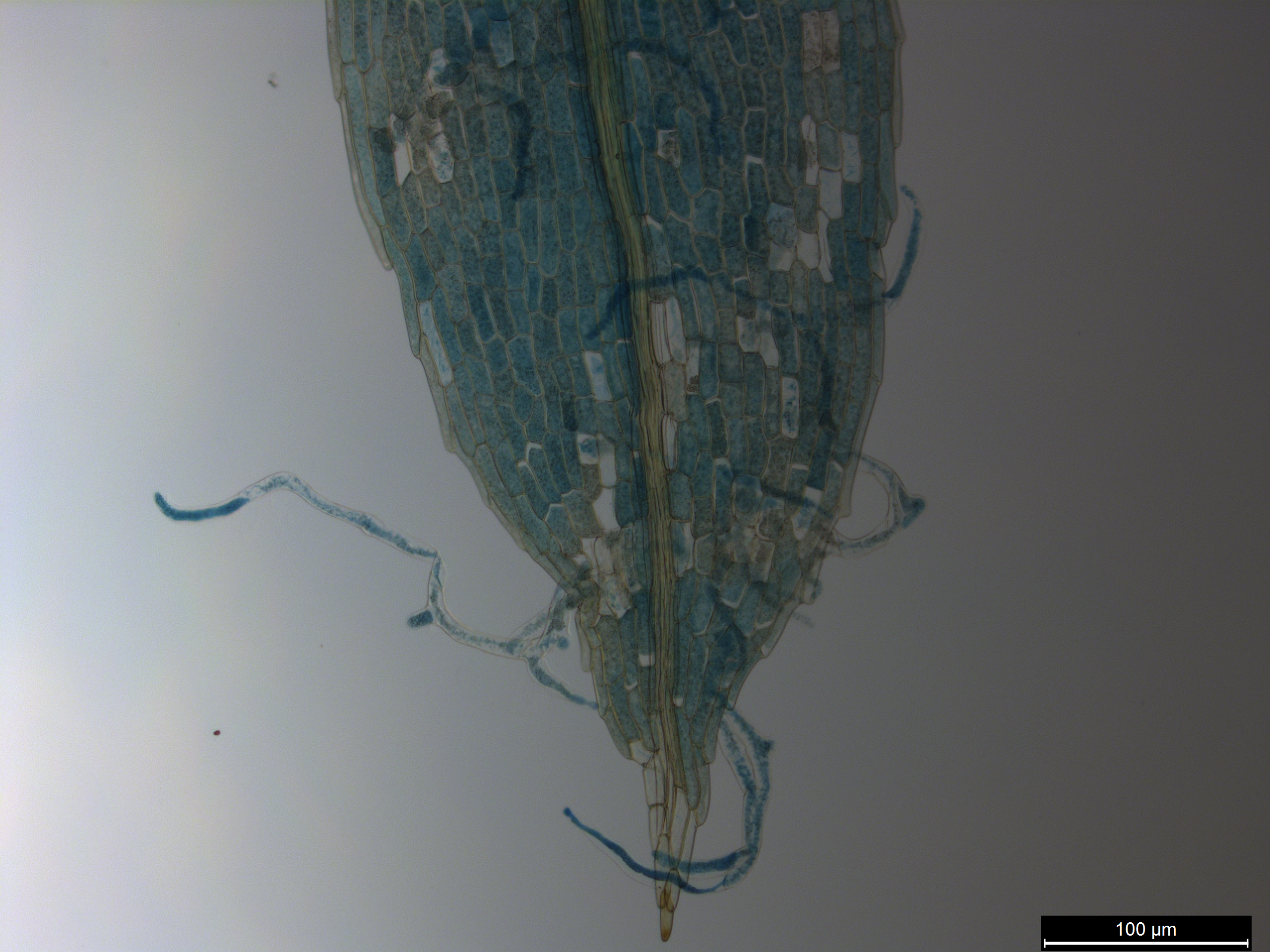

Supplement: Supplementary file 4 — Source Data [file 41467_2020_15967_MOESM4_ESM.zip › Raw data/Raw data for Supplementary Figures/Supplementary Fig 14e.tif]

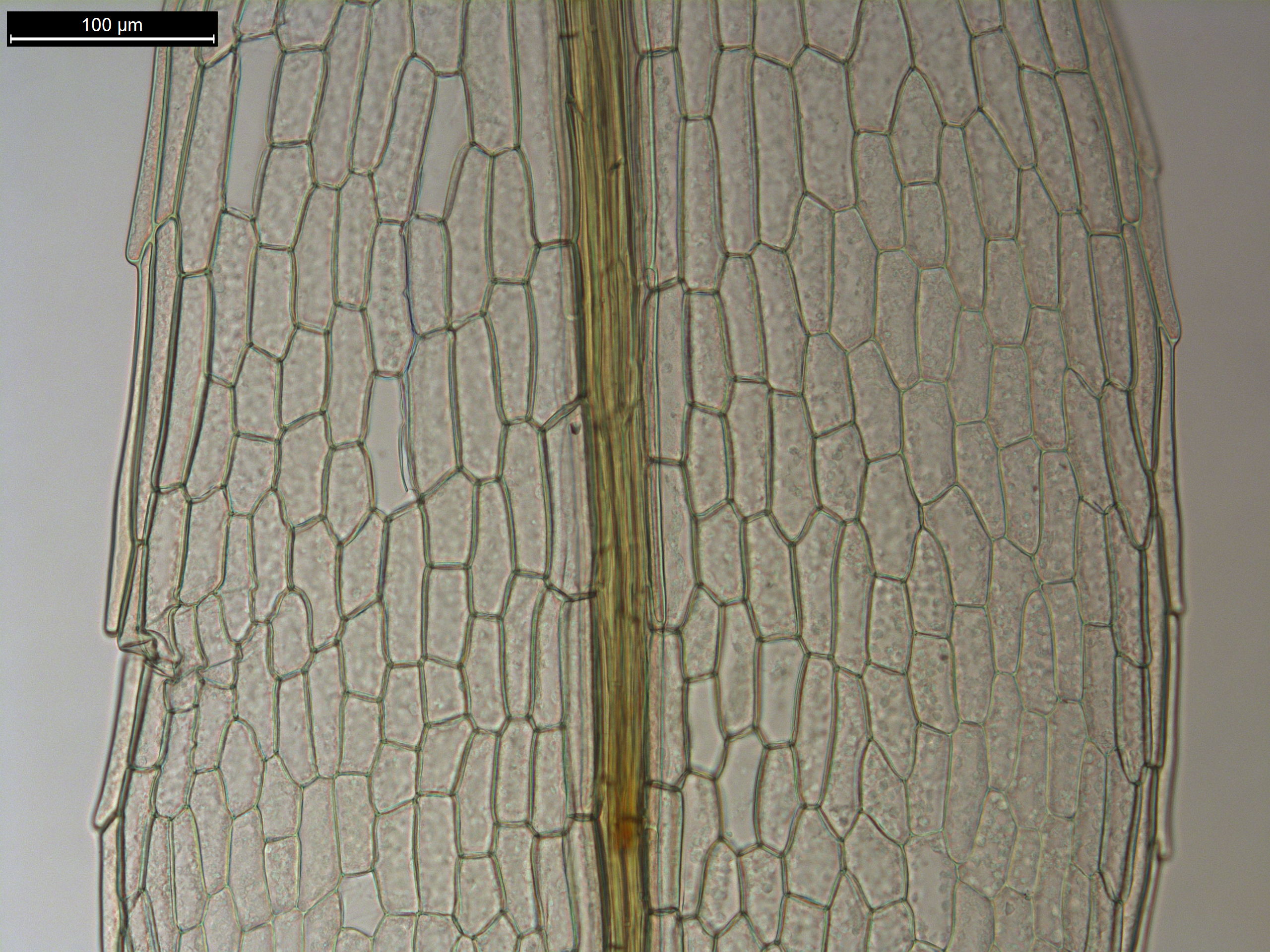

Supplement: Supplementary file 4 — Source Data [file 41467_2020_15967_MOESM4_ESM.zip › Raw data/Raw data for Supplementary Figures/Supplementary Fig 14f.tif]

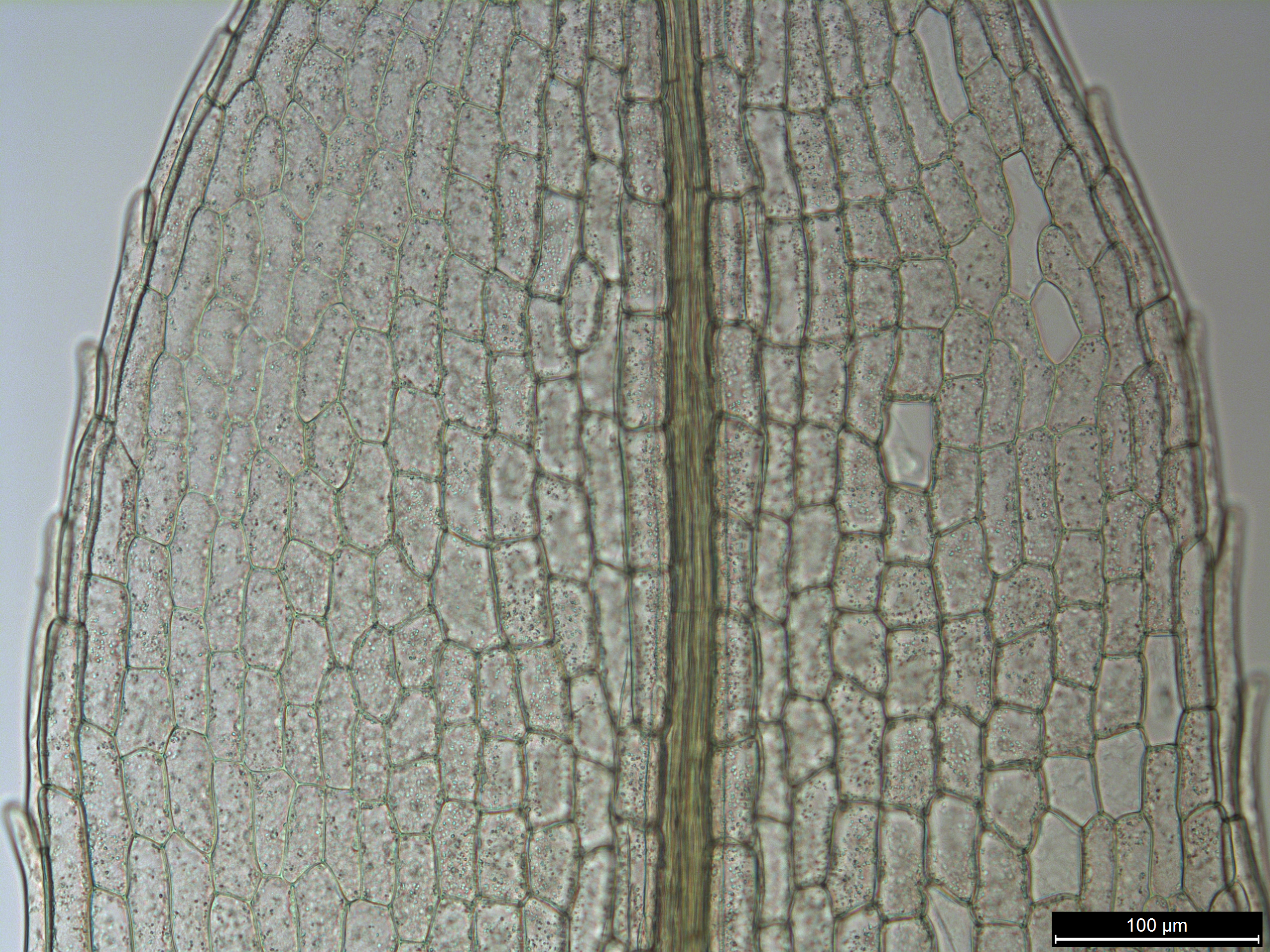

Supplement: Supplementary file 4 — Source Data [file 41467_2020_15967_MOESM4_ESM.zip › Raw data/Raw data for Supplementary Figures/Supplementary Fig 14g.tif]

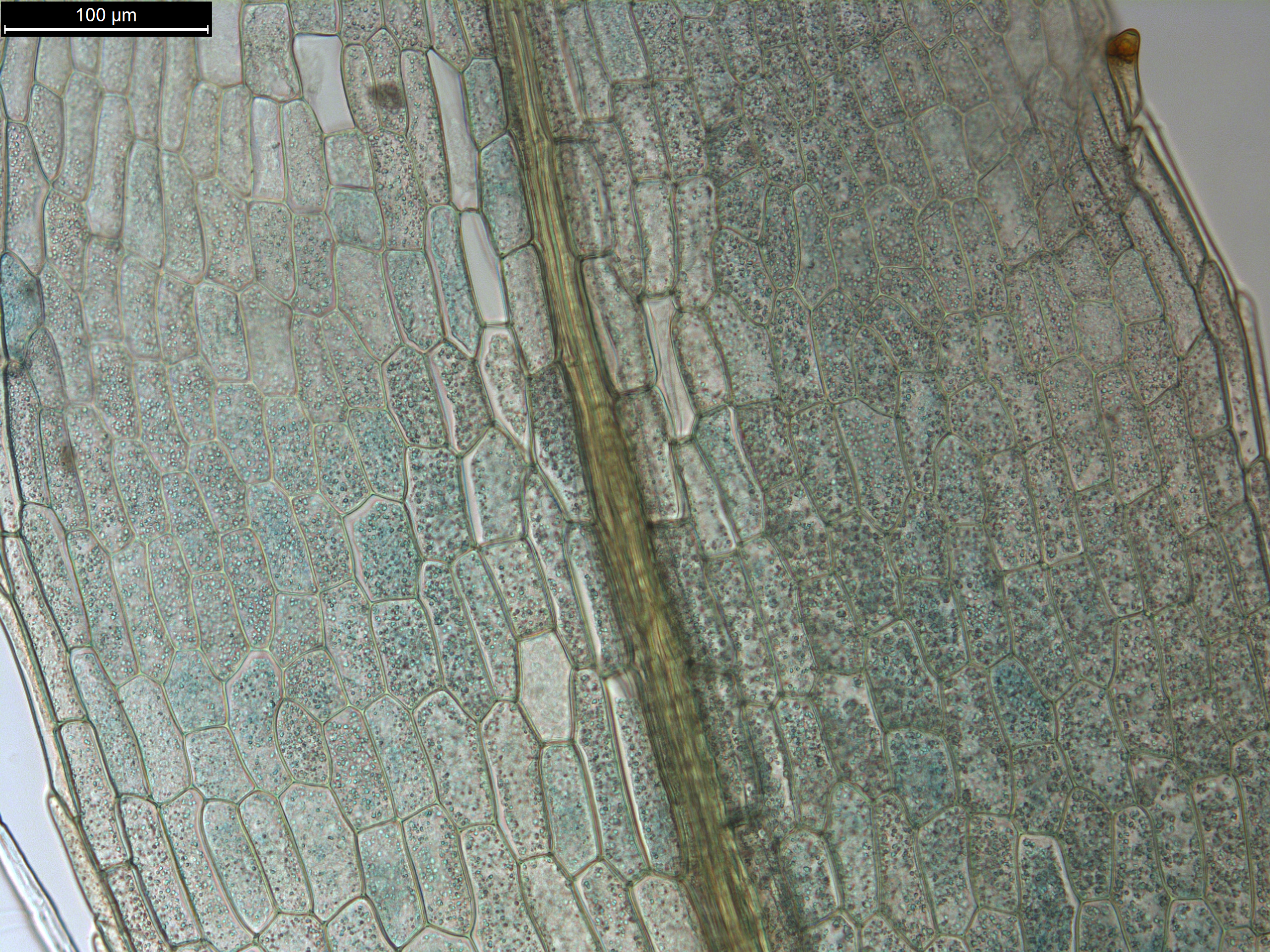

Supplement: Supplementary file 4 — Source Data [file 41467_2020_15967_MOESM4_ESM.zip › Raw data/Raw data for Supplementary Figures/Supplementary Fig 14h.tif]

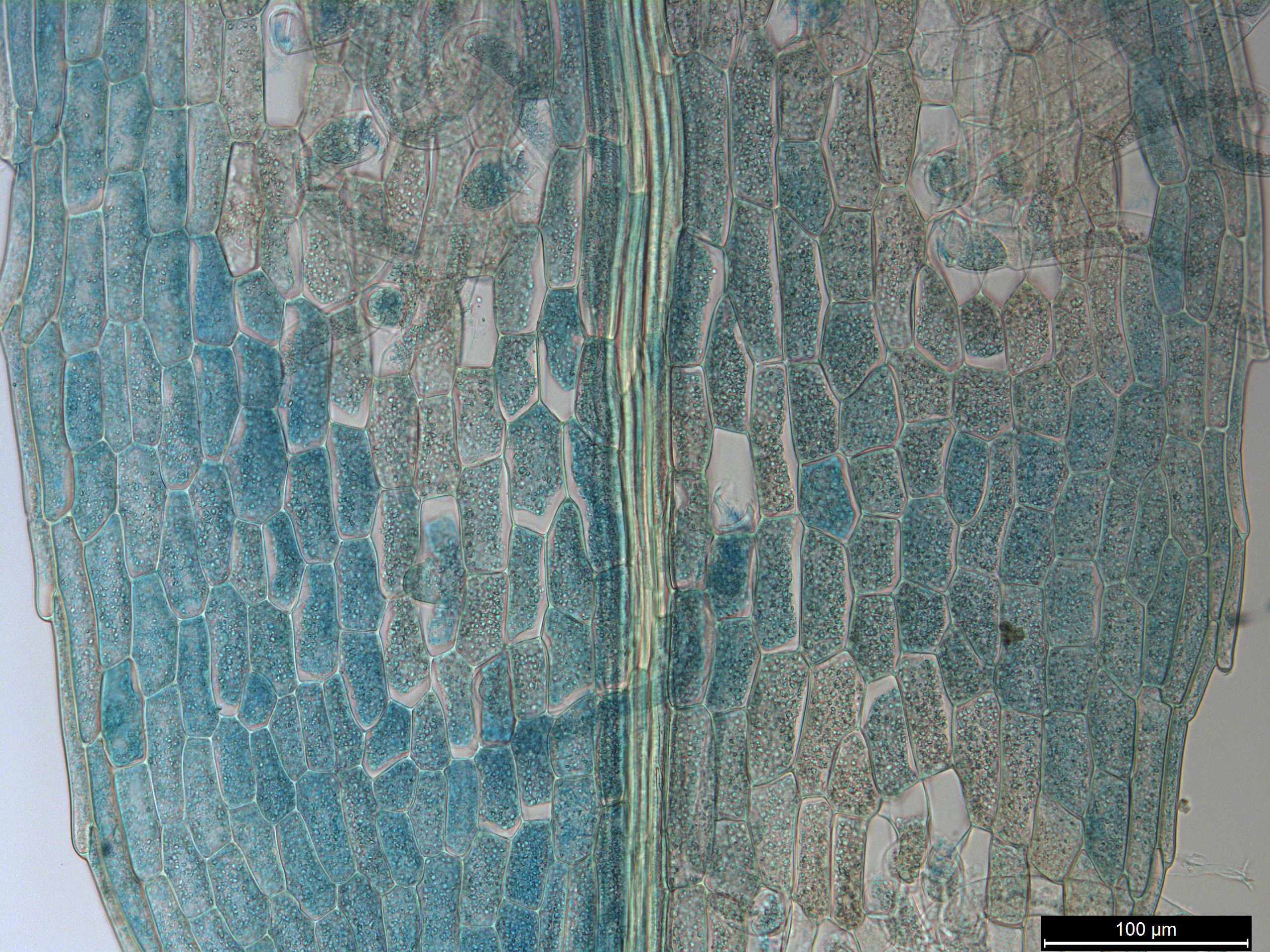

Supplement: Supplementary file 4 — Source Data [file 41467_2020_15967_MOESM4_ESM.zip › Raw data/Raw data for Supplementary Figures/Supplementary Fig 14i.tif]

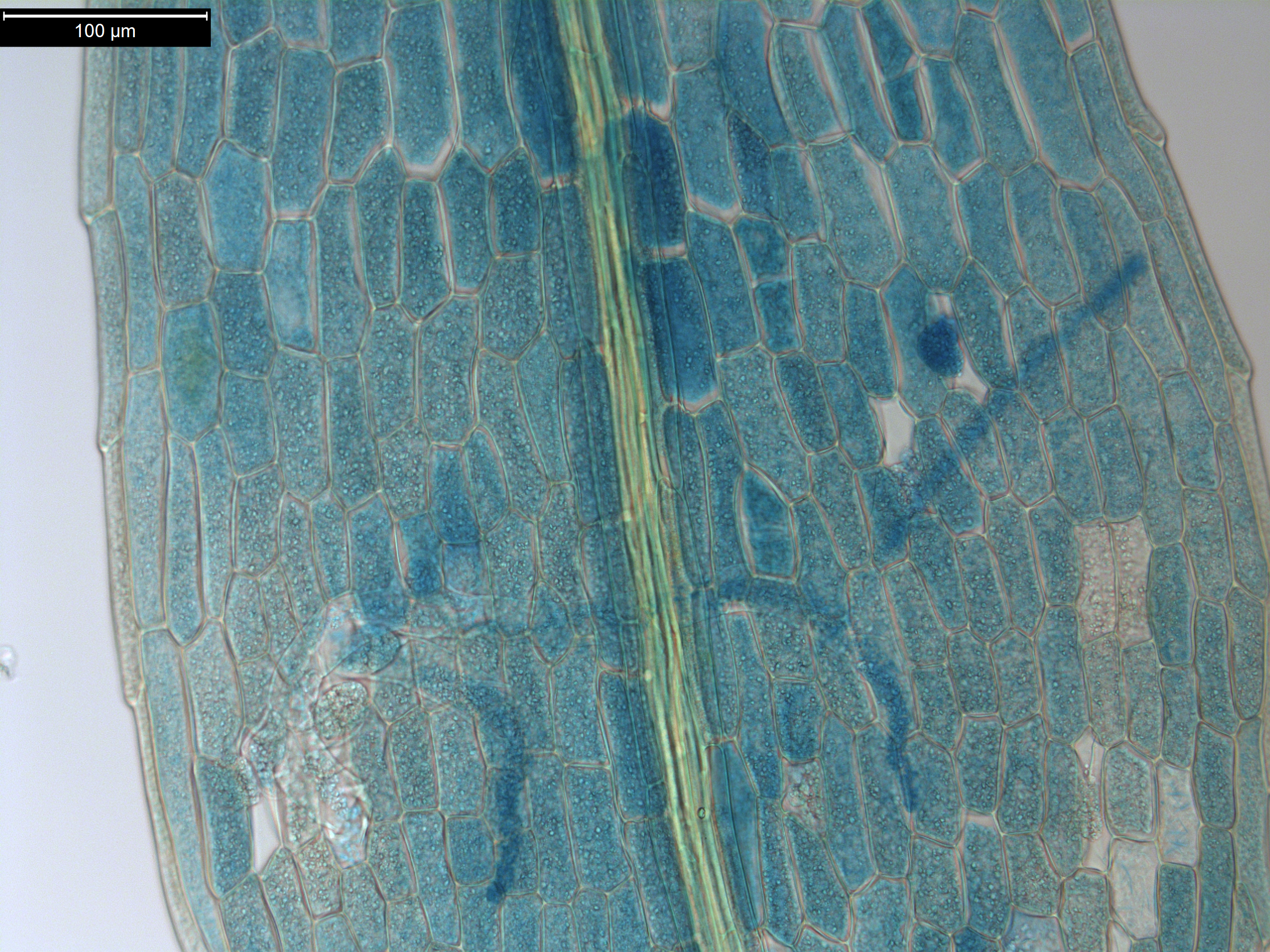

Supplement: Supplementary file 4 — Source Data [file 41467_2020_15967_MOESM4_ESM.zip › Raw data/Raw data for Supplementary Figures/Supplementary Fig 14j.tif]

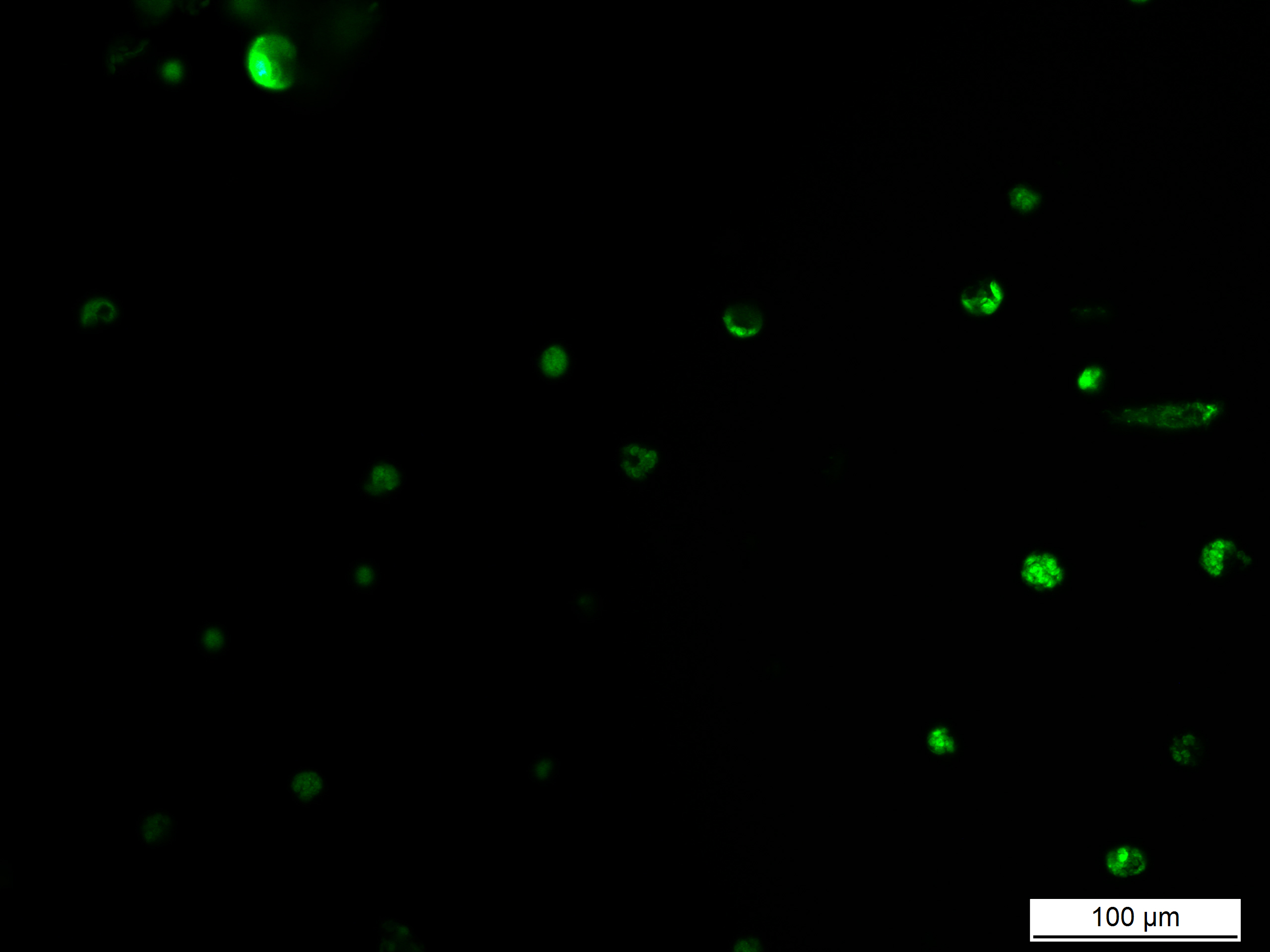

Supplement: Supplementary file 4 — Source Data [file 41467_2020_15967_MOESM4_ESM.zip › Raw data/Raw data for Supplementary Figures/Supplementary Fig 8a.jpg]

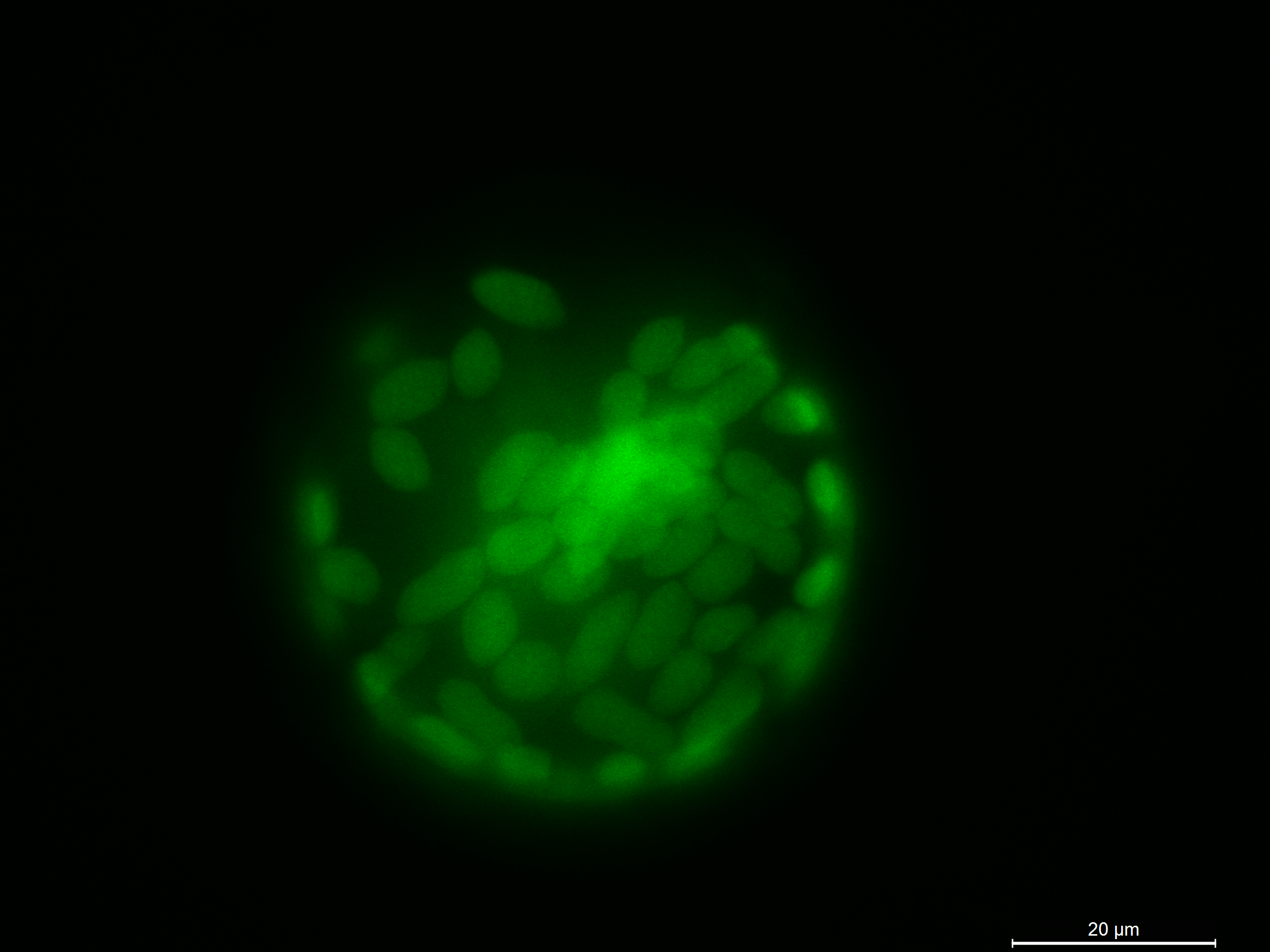

Supplement: Supplementary file 4 — Source Data [file 41467_2020_15967_MOESM4_ESM.zip › Raw data/Raw data for Supplementary Figures/Supplementary Fig 8b.tif]

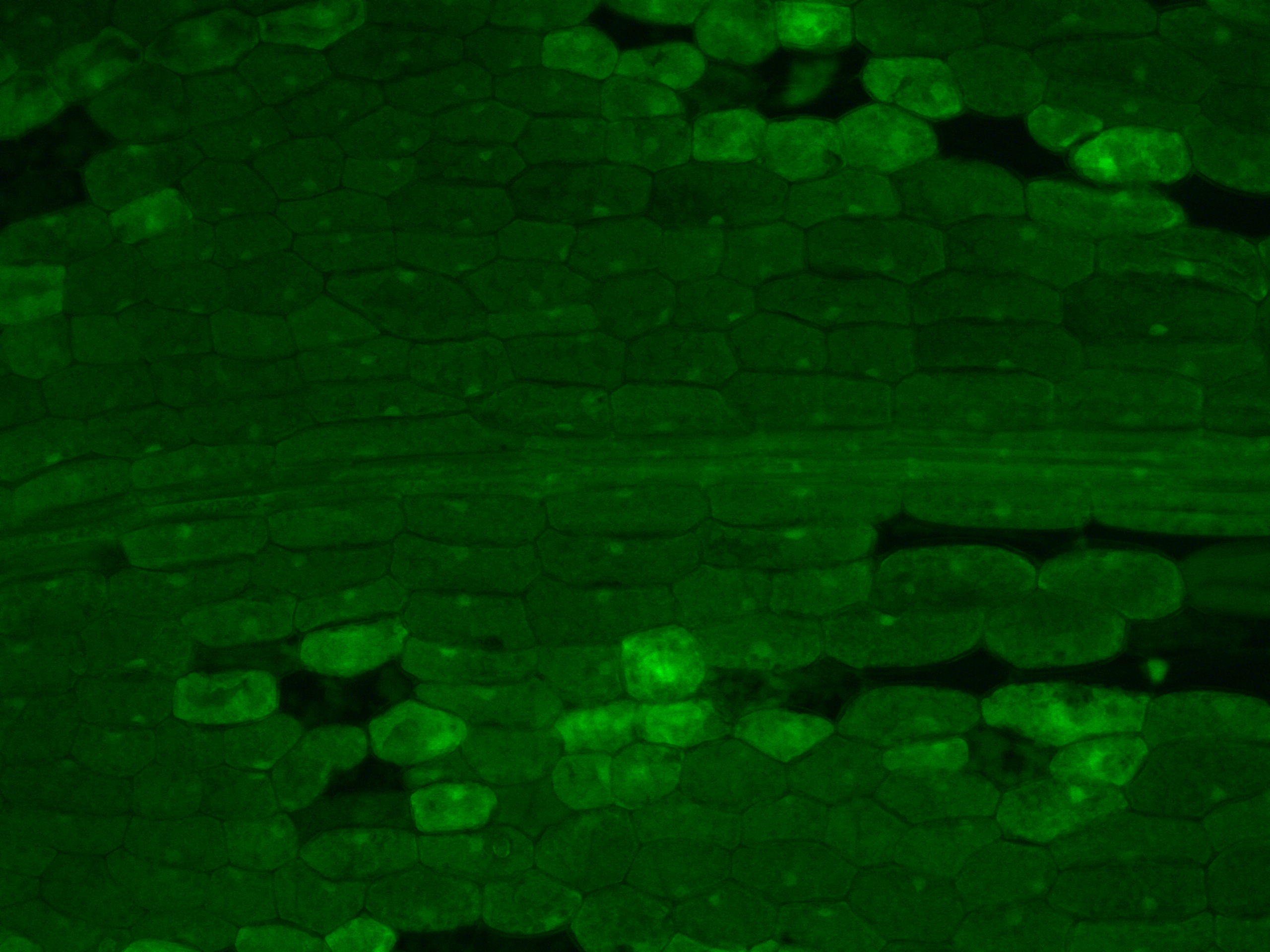

Supplement: Supplementary file 4 — Source Data [file 41467_2020_15967_MOESM4_ESM.zip › Raw data/Raw data for Supplementary Figures/Supplementary Fig 8c.jpg]
